# Supplementary material for: A realist review to explore how low-income pregnant women use food vouchers from the UK’s Healthy Start programme
Source: BMJ Open. 2017 Apr 21;7(4):e013731. doi: 10.1136/bmjopen-2016-013731 (PMC5594208; doi:10.1136/bmjopen-2016-013731)
Supplement: Supplementary data [file bmjopen-2016-013731supp001.docx]

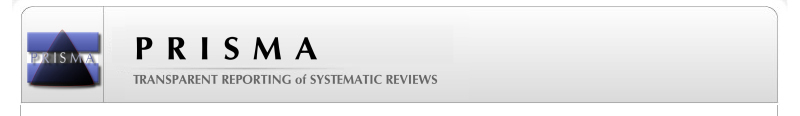
**PRISMA 2009 Flow Diagram**

**Screening**

**Included**

**Eligibility**

**Identification**

Records identified through database searching
(n = 1469)

Additional records identified through other sources
(n = 19)

Records after duplicates removed
(n = 908)

Records screened
(n = 908)

Records excluded
(n = 820)

Full-text articles assessed for eligibility
(n = 88)

Full-text articles excluded,

with reasons

(n = 50):

Relevance score 4/8 or less (n = 33)

Not primary study (n = 15)

Duplication of findings (n = 2)

Studies included in realist synthesis
(n = 38):

Quantitative only (n = 26)

Mixed methods (n = 7)

Qualitative only (n = 4)

Methods unclear (n = 1)
