# Supplementary material for: A realist review to explore how low-income pregnant women use food vouchers from the UK’s Healthy Start programme
Source: BMJ Open. 2017 Apr 21;7(4):e013731. doi: 10.1136/bmjopen-2016-013731 (PMC5594208; doi:10.1136/bmjopen-2016-013731)
Supplement: Supplementary data [file bmjopen-2016-013731supp002.pdf]

Supplementary file 1. Characteristics of studies included in review (n=38)

Full citation	Intervention (country)	Study design and data collection methods	Contribution to data extraction (C/M/O) and synthesis (programme theories 1-3)					
			C	M	O	1	2	3
Department of Health. Healthy Start: Retailer research summary. London, 2012.	Healthy Start (UK)	Methods unclear	✓	✓			✓	
Hills D, Child C, Junge K, Wilkinson E, Sullivan F. Healthy Start: Rapid evaluation of early impact on beneficiaries, health professionals, retailers and contractors. London: Symbia/Tavistock Institute, 2006.	Healthy Start (UK)	Rapid evaluation; quantitative surveys and qualitative feedback		✓	✓		✓	
Lucas PJ, Jessiman T, Cameron A, et al. Healthy Start Vouchers Study: The Views and Experiences of Parents, Professionals and Small Retailers in England. Bristol: University of Bristol, 2013.	Healthy Start (UK)	Qualitative study; in-depth interviews	✓	✓	✓	✓	✓	✓
McFadden A, Fox-Rushby J, Green JM, et al. Healthy Start: Understanding the use of vouchers and vitamins. Dundee: University of Dundee, 2013.	Healthy Start (UK)	Focus groups; online consultations; workshops and telephone interviews	✓	✓	✓	✓		
Andreyeva T, Middleton AE, Long MW, et al. Food retailer practices, attitudes and beliefs about the supply of healthy foods, *Public Health Nutr* 2011;14:1024-31.	WIC (US)	Before/after study; structured interviews to assess perceived customer demand for healthy foods (mostly quantitative)		✓	✓		✓	
Andreyeva T, Luedicke J, Henderson KE, et al. Grocery store beverage choices by participants in federal food assistance and nutrition programs, *Am J Prev Med* 2012;43:411-8.	WIC (US)	Cross-sectional study; electronic sales data from WIC retailers			✓			
Andreyeva T, Luedicke J, Tripp AS, et al. Effects of reduced juice allowances in food packages for the women, infants, and children program, *Pediatrics* 2013;131:919-27.	WIC (US)	Before/after study; electronic sales data from WIC retailers			✓			

Full citation	Intervention (country)	Study design and data collection methods	Contribution to data extraction (C/M/O) and synthesis (programme theories 1-3)					
			C	M	O	1	2	3
Andreyeva T, Luedicke J. Federal food package revisions: effects on purchases of whole-grain products, *Am J Prev Med* 2013;45:422-9.	WIC (US)	Before/after study; electronic sales data from WIC retailers			✓			
Andreyeva T, Luedicke J, Henderson KE, et al. The positive effects of the revised milk and cheese allowances in the special supplemental nutrition program for women, infants, and children, *Journal of the Academy of Nutrition & Dietetics* 2014;114:622-30.	WIC (US)	Before/after study; electronic sales data from WIC retailers			✓			
Andreyeva T, Luedicke J. Incentivizing fruit and vegetable purchases among participants in the Special Supplemental Nutrition Program for Women, Infants, and Children, *Public Health Nutr* 2015;18:33-41.	WIC (US)	Before/after study; electronic sales data from WIC retailers			✓			
Ayala GX, Laska MN, Zenk SN, et al. Stocking characteristics and perceived increases in sales among small food store managers/owners associated with the introduction of new food products approved by the Special Supplemental Nutrition Program for Women, Infants, and Children, *Public Health Nutr* 2012;15:1771-9.	WIC (US)	Cross-sectional study; in-depth interviews to assess perceived changes in sales of WIC foods (quantitative data from closed questions)			✓			
Bailey LB, O'Farrell-Ray B, Mahan CS, et al. Vitamin B6, iron and folacin status of pregnant women, *Nutr Res* 1983;3:783-93.	WIC (US)	Cross-sectional study; dietary assessment using 24 hour recalls; blood tests for nutrient status			✓			
Bertmann FM, Barroso C, Ohri-Vachaspati P, et al. Women, infants, and children cash value voucher (CVV) use in Arizona: a qualitative exploration of barriers and strategies related to fruit and vegetable purchases, *Journal of Nutrition Education & Behavior* 2014;46:S53-8.	WIC (US)	Qualitative study; focus groups to explore attitudes and behaviours related to FV voucher use	✓		✓			

**Full citation**	**Intervention (country)**	**Study design and data collection methods**	**Contribution to data extraction (C/M/O) and synthesis (programme theories 1-3)**					
			**C**	**M**	**O**	**1**	**2**	**3**
Black MM, Hurley KM, Oberlander SE, et al. Participants' comments on changes in the revised special supplemental nutrition program for women, infants, and children food packages: the Maryland food preference study, *J Am Diet Assoc* 2009;109:116-23.	WIC (US)	Cross-sectional study; questionnaire to assess current and anticipated consumption; focus groups to explore reactions to proposed changes to WIC package			✓			
Chen DY, Gazmararian JA. Impact of personal preference and motivation on fruit and vegetable consumption of WIC-participating mothers and children in Atlanta, GA, *Journal of Nutrition Education & Behavior* 2014;46:62-7.	WIC (US)	Cross-sectional study; questionnaire to assess FV consumption			✓			
Endres J, Dunning S, Poon SW, et al. Older pregnant women and adolescents: nutrition data after enrollment in WIC, *J Am Diet Assoc* 1987;87:1011.	WIC (US)	Cross-sectional study; dietary assessment using 24 hour recalls			✓			
Endres JM, Sawicki M, Casper JA. Dietary assessment of pregnant women in a supplemental food program. *J Am Diet Assoc* 1981;79:121-6.	WIC (US)	Cross-sectional study; dietary assessment using 24 hour recalls			✓			
Ettienne-Gittens R, McKyer ELJ, Odum M, et al. Rural versus Urban Texas WIC participants' fruit and vegetable consumption, *Am J Health Behav* 2013;37:130-40.	WIC (US)	Cross-sectional study; questionnaire to assess FV consumption and variety			✓			
Farrior ES, Ruwe CH. Women, infants and children program. Prenatal participation and dietary intakes, *Nutr Res* 1987;7:451-549.	WIC (US)	Before/after study; dietary assessment using 24 hour recalls			✓			
Gittelsohn J, Laska MN, Andreyeva T, et al. Small retailer perspectives of the 2009 women, infants and children program food package changes, *Am J Health Behav* 2012;36:655-65.	WIC (US)	Qualitative study; in-depth interviews with small store owners and managers	✓	✓	✓		✓	

**Full citation**	**Intervention (country)**	**Study design and data collection methods**	**Contribution to data extraction (C/M/O) and synthesis (programme theories 1-3)**					
			**C**	**M**	**O**	**1**	**2**	**3**
Herman DR, Harrison GG, Afifi AA, et al. The Effect of the WIC Program on Food Security Status of Pregnant, First-Time Participants, *Family Economics & Nutrition Review* 2004;16:21.	WIC (US)	Longitudinal study; US Food Security Survey Module; qualitative interviews on use of WIC food package as income transfer (presented as quantitative data)			✓	✓		
Herman DR, Harrison GG, Jenks E. Choices made by low-income women provided with an economic supplement for fresh fruit and vegetable purchase, *J Am Diet Assoc* 2006;106:740-4.	WIC (US)	Cross-sectional study; data from retailers on FV purchases			✓			
Herman DR, Harrison GG, Afifi AA, et al. Effect of a targeted subsidy on intake of fruits and vegetables among low-income women in the Special Supplemental Nutrition Program for Women, Infants, and Children, *Am J Public Health* 2008;98:98-105.	WIC (US)	Longitudinal study; dietary assessment using 24 hour recalls			✓			
Horswill LJ, Yap C. Consumption of foods from the WIC food packages of Chinese prenatal patients on the US west coast, *J Am Diet Assoc* 1999;99:1549-53.	WIC (US)	Cross-sectional study; dietary assessment using FFQ	✓		✓			
Kong A, Odoms-Young A, Schiffer LA, et al. Racial/ethnic differences in dietary intake among WIC families prior to food package revisions, *Journal of Nutrition Education & Behavior* 2013;45:39-46.	WIC (US)	Cross-sectional study; dietary assessment using 24 hour recalls	✓		✓			
Kong A, Odoms-Young A, Schiffer LA, et al. The 18-month impact of special supplemental nutrition program for women, infants, and children food package revisions on diets of recipient families, *Am J Prev Med* 2014;46:543-51.	WIC (US)	Longitudinal study; dietary assessment using 24 hour recalls (baseline data from Kong et al. 2013)	✓		✓			

Full citation	Intervention (country)	Study design and data collection methods	Contribution to data extraction (C/M/O) and synthesis (programme theories 1-3)					
			C	M	O	1	2	3
Meiqari L, Torre L, Gazmararian JA. Exploring the Impact of the New WIC Food Package on Low-Fat Milk Consumption Among WIC Recipients: A Pilot Study, *Journal of Health Care for the Poor & Underserved* 2015;26:712-25.	WIC (US)	Before/after study; questionnaire to assess primary consumption of low-fat milk (yes/no)			✓			
Nestor B, McKenzie J, Hasan N, et al. Client satisfaction with the nutrition education component of the California WIC program, *J Nutr Educ* 2001;33:83-94.	WIC (US)	Cross-sectional study; client satisfaction survey; focus groups to assess client experiences and satisfaction	✓	✓	✓			
Odoms-Young A, Kong A, Schiffer LA, et al. Evaluating the initial impact of the revised Special Supplemental Nutrition Program for Women, Infants, and Children (WIC) food packages on dietary intake and home food availability in African-American and Hispanic families, *Public Health Nutr* 2014;17:83-93.	WIC (US)	Longitudinal study; dietary assessment using 24 hour recalls (baseline data from Kong et al. 2013) and questionnaire to assess home food availability			✓			
Pehrsson PR, Moser-Veillon PB, Sims LS, et al. Postpartum iron status in nonlactating participants and nonparticipants in the Special Supplemental Nutrition Program for Women, Infants, and Children, *Am J Clin Nutr* 2001;73:86-92.	WIC (US)	Longitudinal study; iron status using FFQ and blood tests			✓			
Reyes NR, Klotz AA, Herring SJ. A qualitative study of motivators and barriers to healthy eating in pregnancy for low-income, overweight, African-American mothers, *Journal of the Academy of Nutrition & Dietetics* 2013;113:1175-81.	WIC (US)	Qualitative study; semi-structured interviews to explore motivators and barriers to healthy eating	✓	✓	✓			✓

Full citation	Intervention (country)	Study design and data collection methods	Contribution to data extraction (C/M/O) and synthesis (programme theories 1-3)					
			**C**	**M**	**O**	**1**	**2**	**3**
Rush D, Sloan NL, Leighton J, et al. The National WIC Evaluation: evaluation of the Special Supplemental Food Program for Women, Infants, and Children. V. Longitudinal study of pregnant women, *Am J Clin Nutr* 1988;48:439-83.	WIC (US)	Before/after study; food expenditure assessed by recall (and diary)			✓			
Rush D, Kurzon MR, Seaver WB, et al. The National WIC Evaluation: evaluation of the Special Supplemental Food Program for Women, Infants, and Children. VII. Study of food expenditures, *Am J Clin Nutr* 1988;48:512-9.	WIC (US)	Longitudinal study; dietary assessment using 24 hour recalls			✓			
Swensen AR, Harnack LJ, Ross JA. Nutritional assessment of pregnant women enrolled in the Special Supplemental Program for Women, Infants, and Children (WIC), *J Am Diet Assoc* 2001;101:903-8.	WIC (US)	Cross-sectional study; dietary assessment using FFQ; blood tests for iron status			✓			
Treiman K, Freimuth V, Damron D, et al. Attitudes and behaviors related to fruits and vegetables among low-income women in the WIC program, *J Nutr Educ Behav* 1996;28:149-56.	WIC (US)	Qualitative study; focus groups to explore attitudes and behaviours related to FV; central location intercept interviews (questionnaires)	✓	✓	✓	✓		✓
Watts V, Rockett H, Baer H, et al. Assessing diet quality in a population of low-income pregnant women: a comparison between Native Americans and whites, *Maternal & Child Health Journal* 2007;11:127-36.	WIC (US)	Cross-sectional study; FFQ to assess diet quality			✓			
Whaley SE, Ritchie LD, Spector P, et al. Revised WIC food package improves diets of WIC families, *Journal of Nutrition Education & Behavior* 2012;44:204-9.	WIC (US)	Cross-sectional study; questionnaire to assess usual dietary habits and changes compared to 6 months earlier			✓			

**Full citation**	**Intervention (country)**	**Study design and data collection methods**	**Contribution to data extraction (C/M/O) and synthesis (programme theories 1-3)**					
			**C**	**M**	**O**	**1**	**2**	**3**
Wunderlich SM, Hongu NK, Courter A, et al. Nutrient intake and nutritional status of low-income pregnant women, *Topics in Clinical Nutrition* 1996;12:66-74.	WIC (US)	Cross-sectional study; dietary assessment using 24 hour recalls			✓			

WIC = Special Supplemental Program for Women, Infants, and Children; FV = fruits and vegetables; Programme theories: 1. Prioritisation of resources; 2. Bending the rules; 3. Disempowerment
