# Supplementary material for: A realist review to explore how low-income pregnant women use food vouchers from the UK’s Healthy Start programme
Source: BMJ Open. 2017 Apr 21;7(4):e013731. doi: 10.1136/bmjopen-2016-013731 (PMC5594208; doi:10.1136/bmjopen-2016-013731)
Supplement: Author's manuscript [file bmjopen-2016-013731.draft_revisions.pdf]

BMJ Open

A realist review to explore how low-income pregnant women use food vouchers from the UK's Healthy Start programme

Journal:	*BMJ Open*
Manuscript ID	bmjopen-2016-013731
Article Type:	Research
Date Submitted by the Author:	03-Aug-2016
Complete List of Authors:	Ohly, Heather; University of Central Lancashire, College of Health and Wellbeing<br>Crossland, Nicola; University of Central Lancashire, College of Health and Wellbeing<br>Lowe, Nicola; University of Central Lancashire, College of Health and Wellbeing<br>Dykes, Fiona; University of Central Lancashire, College of Health and Wellbeing<br>Hall-Moran, Victoria; University of Central Lancashire, College of Health and Wellbeing
**Primary Subject Heading**:	Public health
Secondary Subject Heading:	Nutrition and metabolism
Keywords:	pregnant, women, food, vouchers, realist, review

SCHOLARONE™ Manuscripts

For peer review only

Title of the article

A realist review to explore how low-income pregnant women use food vouchers from the UK's Healthy Start programme

Full name, postal address, email and telephone number of corresponding author:

Heather Ohly, Brook Building 215, College of Health and Wellbeing, University of Central Lancashire, Preston, UK

HOhly@uclan.ac.uk

07866 318014

Full name, department, institution, city and country of all co-authors:

Heather Ohly, College of Health and Wellbeing, University of Central Lancashire, Preston, UK

Nicola Crossland, College of Health and Wellbeing, University of Central Lancashire, Preston, UK

Nicola Lowe, College of Health and Wellbeing, University of Central Lancashire, Preston, UK

Fiona Dykes, College of Health and Wellbeing, University of Central Lancashire, Preston, UK

Victoria Hall Moran, College of Health and Wellbeing, University of Central Lancashire, Preston, UK

Word count, excluding title page, abstract, references, figures and tables:

4076 words

For peer review only

ABSTRACT

Objectives: To explore how low-income pregnant women use Healthy Start food vouchers, the potential impacts of the programme, which women might experience these impacts and why.

Design: A realist review.

Eligibility criteria for selecting studies: Primary or empirical studies (of any design) were included if they contributed relevant evidence or insights about how low-income women use food vouchers from the Healthy Start (UK) or the Special Supplemental Nutrition Program for Women, Infants and Children (US) programmes. The assessment of 'relevance' was deliberately broad to ensure that reviewers remained open to new ideas from a variety of sources of evidence.

Analysis: A combination of evidence synthesis and realist analysis techniques was used to modify, refine and substantiate programme theories, which were constructed as explanatory 'context – mechanism – outcome' (CMO) configurations.

Results: 38 primary studies were included in this review: four studies on Healthy Start and 34 studies on WIC. Two main outcome strands were identified: nutritional benefits (intended) and financial assistance (unintended). Three 'evidence-informed' programme theories were proposed to explain how aspects of context (and mechanisms) may generate these outcomes: the 'relative value' of healthy eating (prioritisation); retailer discretion (pressure to 'bend the rules'); the influence of other family members (disempowerment).

Conclusions: This realist review suggests that some low-income pregnant women may use Healthy Start vouchers to increase their consumption of fruits and vegetables and plain cow's milk, whereas others may use them to reduce food expenditure and save money for other things. Further evidence is needed to understand which women are most (and least) likely to experience the intended outcome of nutritional benefits.

Key words: pregnant, women, food, vouchers, realist, review

For peer review only

Strengths and limitations of this study:

- First study to articulate, develop and test programme theories about Healthy Start
- Inclusion of relevant studies from a similar programme in the US (WIC)
- Some evidence not transferable due to population and programme differences
- Insufficient evidence to link CMO configurations to sociodemographic and cultural characteristics

For peer review only

BACKGROUND

Healthy Start is the United Kingdom (UK) government's food voucher programme for low-income pregnant women and young children. It provides weekly food vouchers worth £3.10 during pregnancy (£6.20 for baby under one year; £3.10 for child aged 1-4 years) which can be exchanged for fruits and vegetables, plain cow's milk or infant formula. Eligibility criteria are income-based and retailers must be registered to accept the vouchers. The scheme also offers free vitamins for eligible women and children, but there have been problems with uptake of vitamins.[1] Some areas offer free vitamins to all pregnant women and young children in their area and the option of universalising Healthy Start vitamins remains under review (at the time of writing) by the Chief Medical Officer. This review explores the food voucher component of the programme only.

Healthy Start aims to provide a "basic nutritional safety net" and to encourage "women and families to make positive nutritional choices affecting their longer term health" (page 4).[2] Therein lies an implicit programme assumption that women will use the vouchers to purchase target foods (ideally in greater quantities than they did before) leading to nutritional benefits. However, since the introduction of Healthy Start in 2006, there has been no robust evaluation of its impact on nutritional outcomes – despite recommendations published in an early scoping review.[3] This review was undertaken to explore how low-income pregnant women use Healthy Start food vouchers, the potential impacts of the programme, which women might experience these impacts and why.

The realist approach was adopted because it is based on the understanding that different individuals or groups of individuals are likely to respond to any programme (or intervention) in different ways. The purpose of realist synthesis is to "articulate underlying programme theories and then to interrogate the existing evidence to find out whether and where these theories are pertinent and productive" (page 74).[4] The underlying principle is that outcomes (O) are caused by mechanisms (M: defined as the reasoning and reactions of individuals in response to the resources offered by the programme), and mechanisms may be 'triggered' by aspects of context (C: defined as pre-existing conditions into which the programme is introduced).[4] As realist synthesis progresses, theories that originate as ideas or hypotheses transform into evidence-based explanations for how the programme works.

For peer review only

The review questions are:

1. How do low-income pregnant women use food vouchers from the Healthy Start programme?
2. What are the nutritional benefits for women, and are there any alternative outcomes or unintended consequences?
3. How do variations in context influence (enable or constrain) the mechanisms and outcomes for women?

It was clear that additional sources of empirical evidence would be needed to explore these research questions, given the limited number of studies published on Healthy Start. The most obvious source of potentially relevant evidence was the Special Supplemental Nutrition Program for Women, Infants and Children (WIC) in the United States (US). WIC is the only other national food subsidy programme for low-income women of childbearing age, including pregnant and postpartum women, and young children. It was first introduced in 1972 and revised in 2009 to reflect current dietary guidelines.[5] The WIC food package for pregnant women is different to Healthy Start, with 'maximum monthly allowances' for a range of foods (reduced-fat milk, whole grain cereals, eggs and pulses) and 'cash value vouchers' for fruits and vegetables. The other main programme difference is mandatory WIC nutrition education for all beneficiaries. There are also many contextual differences between the US and the UK, such as socio-demographic, cultural, geographical and political characteristics. Despite these differences, there are also likely to be similarities in terms of how low-income women respond to the programme (i.e. mechanisms).

METHODS

The original protocol for this realist review was registered with the International Prospective Register of Systematic Reviews (PROSPERO 2014: CRD42014015050). Ethical approval was obtained from the University of Central Lancashire Science, Technology, Engineering, Medicine and Health (STEMH) Ethics Committee in May 2015.

Programme theory development

This review is about a programme that already exists, with implicit theories and assumptions about how it works and what effects it may have on beneficiaries.[4] Therefore, we used a 'bottom up' approach to theory development in this review, as described by Shearn and

For peer review only

Allmark (Realist Research Seminar Series, University of Sheffield, 2016). Candidate theories were identified using information derived from academic and grey literature on Healthy Start, an intervention mapping exercise, existing knowledge, creative thinking and discussions among the review team. Consultations with stakeholders were conducted in person and by email. The stakeholder group included six midwives, two academics and two public health practitioners, who all shared first hand experiences and insights about how the Healthy Start programme works in practice.

Search strategy

Separate searches were conducted for Healthy Start and WIC:

Healthy Start – Studies were identified through manual, purposive, snowball and citation searches (January to March 2015). The search terms used initially were 'Healthy Start' and 'UK' because there is another programme called Healthy Start in the US, which aims to prevent infant mortality. More precise search terms were not needed due to the paucity of empirical studies and familiarity with the literature.

WIC – A broad search strategy was devised in collaboration with an Information Specialist in the Collaboration for Leadership in Applied Health Research and Care (CLAHRC) North West Coast. This strategy was adapted and run in six electronic databases in September 2015: MEDLINE, EMBASE, CINAHL, Open Grey, ETHOS and PubMed. Table 1 shows the search terms used in MEDLINE. No date or language restrictions were used. Reference lists of included studies were checked for additional studies. An online list of WIC studies was also checked for additional studies.[6]

Table 1. Search strategy used in MEDLINE to identify WIC studies

#	Search terms	Results
1	WIC.tw.	1008
2	(nutrition or food or voucher or "nutrition program").tw.	377002
3	1 and 2	599
4	(Special* adj4 Supplement* adj4 Nutrit* adj4 Program* adj4 Women* adj4 Infant* adj4 Child*).tw.	415
5	3 or 4	688

Inclusion criteria

For peer review only

Primary or empirical studies (of any design) were included if they contributed relevant evidence or insights about how low-income women use food vouchers from the Healthy Start (UK) or WIC (US) programmes. An assessment of 'relevance' is essential in realist synthesis, to ensure that all included studies contribute to theory development, refinement and testing.[4, 7] In this review, the interpretation of 'relevance' was deliberately broad to ensure that reviewers remained open to new ideas from a variety of sources of evidence. A bespoke system was used to maintain a consistent and transparent approach. Table 2 shows the questions used to assess relevance. Studies that scored 5/8 or more (based on the total number of yes answers) were included.

Table 2. Questions used to assess the relevance of primary studies

#	Question:
1	Do the research questions or study aims refer to Healthy Start or WIC?
2	Does the study focus on the food voucher (CVV or food package for WIC) component of the programme?
3	Does the study focus on beneficiaries rather than eligibility status?
4	Does the sample include pregnant women?
5	If the sample does not include pregnant women, could some of the findings be generalizable to pregnant women?
6	Does the study report women's food or nutrient intakes (measured or perceived)?
7	Does the study provide any insights about how food vouchers are used?
8	Does the study provide any insights about which women may benefit most/least and why?

Study selection

Results from the WIC database searches were uploaded into RefWorks (web version; ProQuest; Michigan, US) and screened using titles and abstracts. Studies that appeared to meet the inclusion criteria were obtained as full text articles. Studies for which insufficient information was available to determine relevance were also obtained as full text articles. The full text screening process was fully documented using Microsoft Excel 2013 (version 15.0.4815.1001; Microsoft Corporation), including assessments of relevance and reasons for exclusions. The same criteria were applied to studies of Healthy Start. Study selection was

For peer review only

completed by the lead reviewer (HO) and double checked by a second reviewer (VM). Any disagreements were resolved by discussion.

Data extraction

Quantitative data on women's nutritional outcomes were extracted using bespoke tables in Microsoft Word 2013 (version 15.0.4815.1001; Microsoft Corporation). Other non-relevant data were not extracted. Qualitative data, textual descriptions of findings, and author interpretations were extracted using MAXQDA 11 (version 11; VERBI Software GmbH; Berlin). A coding system was created with three main headings: context, mechanisms and outcomes. Subheadings were added deductively (based on early theories) and inductively (as new themes emerged from the data). Data extraction was completed by the lead reviewer (HO) and a sample was double checked by a second reviewer (NC).

Quality appraisal

Studies were not formally appraised at the data extraction stage, as would be the case in traditional systematic reviews. Instead, an assessment of 'rigour' was used to judge the credibility and trustworthiness of the evidence as it was integrated into the analysis and synthesis.[4, 7] This assessment was not scored because weaker studies were still included, but it meant that methodological limitations were acknowledged and study findings were not over-interpreted. Table 3 shows the questions used to assess rigour. Quality appraisal was completed by the lead reviewer (HO) and double checked by second reviewers (NL/VM).

Table 3. Questions used to assess the rigour of primary studies

#	Question:
1	Are the study methods clearly reported (including study design, recruitment, data collection and analysis)?
2	Are the study methods appropriate to answer the research questions?
3	Are the sample characteristics reported to enable judgements about generalisability?
4	Are the study findings and conclusions supported by raw data?
5	Are the study limitations acknowledged and clearly reported?

Analysis and synthesis

For peer review only

The analysis and synthesis 'stage' of the review involved gradual and iterative theory development, whereby evidence from primary studies was used to modify, refine and substantiate programme theories about how low-income pregnant women use Healthy Start vouchers, in what circumstances, and why. Theories were constructed as explanatory 'context – mechanism – outcome' (CMO) configurations, usually by starting with the outcome and working backwards.[8] The main focus of the analysis was searching for evidence to support the proposed causal linkages between context, mechanisms and outcomes. A combination of evidence synthesis and realist analysis techniques was used:

1. Narrative synthesis of quantitative data on women's nutritional outcomes; meta-analysis was not appropriate due to heterogeneity of study designs and data collection methods (and was beyond the scope of this review).
2. Thematic synthesis of qualitative data, by creating codes and themes (as described under data extraction) and then 'going beyond' the interpretations of the original studies to generate new understandings or hypotheses.[9]
3. Creative theorising or 'retroduction' by the lead reviewer (HO) in collaboration with the review team and the stakeholder group, such that resulting programme theories were to varying degrees 'evidence-informed' and 'evidence-inspired'. Where individual extracts of data only supported part of the CMO configuration, it was necessary to make logical inferences about the complete causal pathways and explanations.[10]

RESULTS

Search results and study characteristics

A total of 908 records were identified through the two separate searches. After screening titles and abstracts, 88 records were obtained in full text format. Fifty full text articles were excluded based on the assessment of relevance (n=33) or because they were not primary studies (n=15) or the findings were duplicated (n=2). Therefore, 38 primary studies were included in this review: four studies on Healthy Start[11-14] and 34 studies on WIC[15-48] (see PRISMA Flow Diagram).

Outcomes: intended and unintended

This section describes the outcomes that were identified, prioritised and evidenced in this realist review. During the theory development stage, the decision was made to focus on the final outcomes of low-income pregnant women as the first beneficiaries of the programme. In

For peer review only

other words, once an eligible woman has received the food vouchers, how does she use them? This decision reflected the research priorities identified from the literature, the interests of the research team, and the time and resources available to conduct this review. Equally important questions relating to earlier stages of the implementation pathway (such as access to the programme and challenges within the application process) and the outcomes of children who subsequently receive food vouchers were not explored in this review.

Two main outcome strands emerged during the theory development stage and were further substantiated using evidence from primary studies:

1. Women use vouchers to increase consumption of target foods – nutritional benefits.
2. Women use vouchers to reduce food expenditure – financial assistance.

For the purposes of this review, we have assumed that strand 1 is the intended outcome of the programme and strand 2 is an unintended outcome, although this was not explicit in policy documentation. The included studies provided support for both outcome strands.

A total of 25 studies reported women's nutritional outcomes: three studies on Healthy Start and 22 studies on WIC. The Healthy Start studies reported perceived outcomes only; some women said they consumed more cow's milk, fruits and vegetables after receiving Healthy Start vouchers,[11, 12, 14] whereas other women said the vouchers "freed up money to do other things" and "helped them to manage better financially" (page 59).[12] The WIC studies were published between 1981 and 2015, but the most useful data was extracted from two studies comparing women's diets before and after the 2009 WIC revisions, when the 'cash value vouchers' for fruits and vegetables were introduced (there was no allowance for fresh fruits and vegetables before 2009). A longitudinal study of African American and Hispanic women from WIC clinics in Chicago (n=273) found significant dietary improvements for Hispanic mothers (more fruit and reduced fat milk; less saturated fat), but no dietary improvements for African American mothers at six months;[39] there were no dietary improvements in either group compared to baseline at 18 months.[36] A cross-sectional study comparing two random samples of WIC participants in California (both 80% Hispanic) found that women assessed six months after the changes (n=2996) consumed significantly more whole grains, reduced-fat milk and vegetables, and less whole milk, compared to women assessed before the changes (n=3004).[47]

For peer review only

Five studies reported electronic sales data from WIC retailers (one large supermarket chain) in New England, which showed that women's purchasing patterns shifted towards items provided in the WIC package after the 2009 revisions – fruits and vegetables, reduced-fat milk, brown rice, whole grain cereals and bread replaced less nutritious options.[16-20] One study (n=2137) showed that, while total spending on fruits and vegetables increased after 2009, up to 13% fewer purchases were made using non-WIC funds.[20] This implies that some women 'substituted' the method of payment, rather than using WIC to increase the amount of fruits and vegetables purchased. None of these studies reported sample characteristics such as ethnicity. Finally, a mixed methods study of Hispanic and African American pregnant women (n=313) found that two thirds of women reported using WIC vouchers to reduce food expenditure.[31] The money they saved was used to purchase items for the unborn baby, other foods, and for bills and emergencies.

These findings suggest that food vouchers may lead to dietary improvements for some, but not all women. This may be because some women use the vouchers to pay for foods they would previously have bought using cash. The WIC studies described above were not representative of ethnic groups in the UK, and the samples included postpartum as well as pregnant women. However, these studies provide much needed evidence on the potential impact of food vouchers for low-income women, which was not available from the Healthy Start literature alone.

CMO configurations: explanations for outcomes

This section presents three 'evidence-informed' programme theories, which help to explain why different women receive the same Healthy Start vouchers and yet experience different outcomes, due to variations in context and mechanisms. Figure 1 illustrates the key aspects of context, mechanisms and outcomes identified and the proposed causal pathways linking them together. Low income is not included as context because it applies to all women who receive Healthy Start vouchers (to varying extents). As the programme theories explain, some women may achieve nutritional benefits despite low income, and other women may not – this divergence depends on other aspects of context.

1. Prioritisation of resources

Women living on low incomes must constantly prioritise how they spend their money. Food vouchers may be considered as one part of the household resources and decisions must be

For peer review only

made about how best to use the vouchers. A key aspect of context is the ‘relative value’ of healthy eating (compared to other things women value), which can lead women to prioritise in different ways. These contrasting theories suggest that ‘relative value’ operates through the mechanism of ‘prioritisation’ and may generate either of the two outcome strands.

Women who value healthy eating and aspire to eat well during pregnancy [context][45] are more likely to perceive Healthy Start vouchers as an opportunity to achieve health benefits for themselves and their unborn baby [mechanism].[11] The vouchers alleviate the financial barrier associated with healthy eating and make healthy foods seem more affordable [mechanism].[11] Therefore, women who value healthy eating are more likely to prioritise healthy eating [mechanism] and use Healthy Start vouchers to increase consumption of target foods – fruits and vegetables or cow’s milk [outcome].[11]

On the other hand, women may value healthy eating less than other things they want or need to spend money on, which are considered more important or urgent [context].[11, 12] They are more likely to perceive Healthy Start vouchers as a way to save money, which can be redirected and prioritised in other ways [mechanism].[11, 12] These women are more likely to use Healthy Start vouchers to deduct money from the shopping bill, with no increase in consumption of target foods [outcome].[11, 12, 31]

2. Bending the rules

The Healthy Start voucher exchange system relies on registered retailers to verify (visually) at the checkout that women have selected appropriate amounts of appropriate items – fruits and vegetables, plain cow’s milk or infant formula. The vouchers are processed by swiping a barcode that subtracts the voucher value (£3.10) from the total. They are not electronically matched to specific food items. There is a reminder printed on each voucher about which foods may be purchased, along with a warning about prosecution, but some customers (and retailers) appear to disregard this information.

Retailers who are registered to accept Healthy Start vouchers have some discretion over how vigilantly they check what vouchers are spent on [context].[11, 13] Women may put pressure on retailers to ‘bend the rules’ or make exceptions [mechanism].[11, 13-15, 30] Some retailers may decide to ‘turn a blind eye’ because they feel duty bound to help families in whatever ways they can[11] or because they prefer to avoid conflict [mechanisms].[14] This

For peer review only

enables women to exchange the vouchers for alternative food or non-food items [outcomes].[11, 14]

3. Disempowerment

Pregnant women (and later their young children) are the intended beneficiaries of Healthy Start, and the vouchers are addressed to them, but some women may not be empowered to use the vouchers themselves. The name of the beneficiary is not printed on the vouchers and there is nothing to stop other people from using them. Regardless of what is bought with the vouchers, and who benefits, this would surely be considered an unintended outcome of the programme.

Women may not be empowered to make decisions about household resources or food shopping, such as pregnant teenagers who live with their parents [context],[11, 45] or women who live in large, multi-generational households [context].[41] Women who are disempowered are more likely to hand over their Healthy Start vouchers to other family members [mechanism][41] who then decide how they are used [outcome].[41]

DISCUSSION

Statement of principal findings

Low-income pregnant women may use food vouchers from the Healthy Start programme in different ways, for different reasons, leading to different outcomes. The two main outcome strands identified in this review were nutritional benefits (intended) and financial assistance (unintended). Three 'evidence-informed' programme theories were proposed to explain how aspects of context (and mechanisms) may generate these outcomes: the 'relative value' of healthy eating (prioritisation); retailer discretion (pressure to 'bend the rules'); the influence of other family members (disempowerment).

Strengths of this review

This was the first study to use a realist, theory-driven approach to investigate how low-income pregnant women use Healthy Start vouchers, in what circumstances, and why. The inclusion of relevant studies from a similar programme in the US (WIC) provided insights and explanations beyond what was available from the Healthy Start literature. This was the first time that researchers have attempted to articulate, develop and test programme theories

For peer review only

about how Healthy Start works, and we hope this will stimulate discussions about future evaluation needs and programme development.

Limitations of this review

We were aware from the outset that some of the evidence from WIC studies would not be transferable to Healthy Start due to population and programme differences. Only theories that were adequately supported by the existing evidence were presented. Further evidence from the UK is needed to develop some of the other candidate theories we identified. This review focused on women's outcomes from the programme, and the aspects of context explored in our theories were 'individual' (women's values and perceptions) and 'interpersonal' (interactions with retailers and other family members).[4] We did not find sufficient evidence to link our CMO configurations with sociodemographic and cultural characteristics – such as which groups of women are more/less likely to value healthy eating. Finally, we did not explore theories relating to infant feeding (Healthy Start vouchers can be exchanged for infant formula) or children's outcomes.

Comparisons with existing literature and theories

Previous systematic reviews have shown that financial incentives (including taxes, subsidies, vouchers and cash payments) are associated with short term dietary improvements.[49-52] No studies of Healthy Start met the inclusion criteria for these reviews and the impact of the programme on women's nutritional outcomes remains unclear. There is also some uncertainty around the definition and function of an incentive in relation to behaviour change. Incentive Theory states that behaviour is motivated by a desire for rewards or positive reinforcements.[53] Skinner's Operant Conditioning Theory states that behaviours that are reinforced tend to be repeated.[54] A recent taxonomy of behaviour change techniques defined 'incentives' as rewarding and contingent on behaviour change.[55] Healthy Start does not fit this definition, since the vouchers are provided based on income-based eligibility criteria, regardless of how they are subsequently used. However, the dictionary definition of an incentive is broader: "a thing that motivates or encourages someone to do something",[56] which encompasses opportunities for behavior change as well as rewards for behavior change.

Our programme theories suggest that some women may perceive Healthy Start vouchers as an opportunity to improve their diets, but only if they value healthy eating. According to the

For peer review only

Behaviour Change Wheel, ‘opportunity’ is one of the key ingredients for behaviour change.[57] The concept of ‘value’ is also important in Incentive Theory in terms of how effective an incentive might be for different individuals in different situations.[53] Our programme theories also suggest that ‘competing values’ may determine whether women perceive, and take advantage of, the opportunity to improve their diets. Hoddinott et al. used the constructs of ‘idealism’ and ‘realism’ to explain how competing values influence women’s infant feeding decisions and practices.[58] They found that immediate concerns (such as pain, anxiety and lack of sleep) often outweighed the long term health benefits associated with breastfeeding. These competing values were said to be more complex for disadvantaged families who may be dealing with greater adversity. Similarly, low-income women may use their Healthy Start vouchers to save money because they consider other things to be more urgent or important. For these women, it might be unrealistic to anticipate that Healthy Start would lead to nutritional benefits.

CONCLUSION

This realist review suggests that some low-income pregnant women use Healthy Start vouchers to increase their consumption of fruits and vegetables and plain cow’s milk (intended outcome: nutritional benefits) whereas other women use them to reduce food expenditure and save money for other things (unintended outcome: financial assistance). We have identified some aspects of context (the ‘relative value’ of healthy eating, retailer discretion and the influence of other family members) and mechanisms (prioritisation, pressure to ‘bend the rules’ and disempowerment) that are likely to be important in determining these outcomes. Further evidence is needed to understand which women are most (and least) likely to experience the intended outcome of nutritional benefits.

For peer review only

STATEMENTS

Acknowledgements: The authors would like to thank the stakeholder group for their contributions to theory development, and Eleanor Kotas (Information Specialist) for her assistance with the electronic database searches.

Contributors: HO was the lead reviewer, while other authors completed the second reviewer tasks (as indicated in the Methods). All authors contributed to the development of programme theories through regular discussions. HO drafted the initial manuscript and all authors contributed to the final manuscript.

Funding: This work was supported by a PhD Studentship (2014-2017) from the Collaboration for Leadership in Applied Health Research and Care North West Coast (CLAHRC NWC).

Declaration: The views expressed are those of the authors and not necessarily those of the DWP, NHS, NIHR or the Department of Health.

Competing interests: None declared.

Data sharing statement: No additional data are available.

For peer review only

REFERENCES

1 McFadden A, Green JM, McLeish J, et al. Healthy Start vitamins – a missed opportunity: findings of a multimethod study, *BMJ Open* 2015;5.

2 Department of Health. Healthy Start: Equality impact assessment. 2010.

3 Dyson L, Renfrew MJ, Jenkins R, et al. Approaches to evaluating Healthy Start – a scoping review. 2007.

4 Pawson R. Evidence-based policy: a realist perspective. London: Sage 2006:196.

5 Institute of Medicine of the National Academies. WIC Food Packages: Time for a Change. 2005.

6 United States Department of Agriculture: Food and Nutrition Service. WIC Studies: http://www.fns.usda.gov/ops/wic-studies. Date accessed: February 2015.

7 The RAMESES Project. Quality Standards for Realist Synthesis (for researchers and peer-reviewers): http://www.ramesesproject.org/media/RS_qual_standards_researchers.pdf. Date accessed: November 2014.

8 Wong G. Special Invited Editorial: Getting Started With Realist Research, *International Journal of Qualitative Methods* 2015;14.

9 Thomas J, Harden A. Methods for the thematic synthesis of qualitative research in systematic reviews, *BMC Medical Research Methodology* 2008;8:45.

10 Ford JA, Wong G, Jones AP, et al. Access to primary care for socioeconomically disadvantaged older people in rural areas: a realist review, *BMJ Open* 2016;6.

11 Lucas PJ, Jessiman T, Cameron A, et al. Healthy Start Vouchers Study: The Views and Experiences of Parents, Professionals and Small Retailers in England. 2013.

12 McFadden A, Fox-Rushby J, Green JM, et al. Healthy Start: Understanding the use of vouchers and vitamins. 2013.

13 Department of Health. Retailer research summary. 2012.

14 Hills D. Healthy Start: Rapid evaluation of early impact on beneficiaries, health professionals, retailers and contractors. 2006.

15 Andreyeva T, Middleton AE, Long MW, et al. Food retailer practices, attitudes and beliefs about the supply of healthy foods, *Public Health Nutr* 2011;14:1024-31.

16 Andreyeva T, Luedicke J, Henderson KE, et al. Grocery store beverage choices by participants in federal food assistance and nutrition programs, *Am J Prev Med* 2012;43:411-8.

17 Andreyeva T, Luedicke J, Tripp AS, et al. Effects of reduced juice allowances in food packages for the women, infants, and children program, *Pediatrics* 2013;131:919-27.

For peer review only

18 Andreyeva T, Luedicke J. Federal food package revisions: effects on purchases of whole-grain products, *Am J Prev Med* 2013;45:422-9.

19 Andreyeva T, Luedicke J, Henderson KE, et al. The positive effects of the revised milk and cheese allowances in the special supplemental nutrition program for women, infants, and children, *Journal of the Academy of Nutrition & Dietetics* 2014;114:622-30.

20 Andreyeva T, Luedicke J. Incentivizing fruit and vegetable purchases among participants in the Special Supplemental Nutrition Program for Women, Infants, and Children, *Public Health Nutr* 2015;18:33-41.

21 Ayala GX, Laska MN, Zenk SN, et al. Stocking characteristics and perceived increases in sales among small food store managers/owners associated with the introduction of new food products approved by the Special Supplemental Nutrition Program for Women, Infants, and Children, *Public Health Nutr* 2012;15:1771-9.

22 Bailey LB, O'Farrell-Ray B, Mahan CS, et al. Vitamin B6, iron and folacin status of pregnant women, *Nutr Res* 1983;3:783-93.

23 Bertmann FM, Barroso C, Ohri-Vachaspati P, et al. Women, infants, and children cash value voucher (CVV) use in Arizona: a qualitative exploration of barriers and strategies related to fruit and vegetable purchases, *Journal of Nutrition Education & Behavior* 2014;46:S53-8.

24 Black MM, Hurley KM, Oberlander SE, et al. Participants' comments on changes in the revised special supplemental nutrition program for women, infants, and children food packages: the Maryland food preference study, *J Am Diet Assoc* 2009;109:116-23.

25 Chen DY, Gazmararian JA. Impact of personal preference and motivation on fruit and vegetable consumption of WIC-participating mothers and children in Atlanta, GA, *Journal of Nutrition Education & Behavior* 2014;46:62-7.

26 Endres J, Dunning S, Poon SW, et al. Older pregnant women and adolescents: nutrition data after enrollment in WIC, *J Am Diet Assoc* 1987;87:1011.

27 Endres JM, Sawicki M, Casper JA. Dietary assessment of pregnant women in a supplemental food program. *J Am Diet Assoc* 1981;79:121-6.

28 Ettienne-Gittens R, McKyer ELJ, Odum M, et al. Rural versus Urban Texas WIC participants' fruit and vegetable consumption, *Am J Health Behav* 2013;37:130-40.

29 Farrior ES, Ruwe CH. Women, infants and children program. Prenatal participation and dietary intakes, *Nutr Res* 1987;7:451-549.

30 Gittelsohn J, Laska MN, Andreyeva T, et al. Small retailer perspectives of the 2009 women, infants and children program food package changes, *Am J Health Behav* 2012;36:655-65.

For peer review only

31 Herman DR, Harrison GG, Afifi AA, et al. The Effect of the WIC Program on Food Security Status of Pregnant, First-Time Participants, *Family Economics & Nutrition Review* 2004;16:21.

32 Herman DR, Harrison GG, Jenks E. Choices made by low-income women provided with an economic supplement for fresh fruit and vegetable purchase, *J Am Diet Assoc* 2006;106:740-4.

33 Herman DR, Harrison GG, Afifi AA, et al. Effect of a targeted subsidy on intake of fruits and vegetables among low-income women in the Special Supplemental Nutrition Program for Women, Infants, and Children, *Am J Public Health* 2008;98:98-105.

34 Horswill LJ, Yap C. Consumption of foods from the WIC food packages of Chinese prenatal patients on the US west coast, *J Am Diet Assoc* 1999;99:1549-53.

35 Kong A, Odoms-Young A, Schiffer LA, et al. Racial/ethnic differences in dietary intake among WIC families prior to food package revisions, *Journal of Nutrition Education & Behavior* 2013;45:39-46.

36 Kong A, Odoms-Young A, Schiffer LA, et al. The 18-month impact of special supplemental nutrition program for women, infants, and children food package revisions on diets of recipient families, *Am J Prev Med* 2014;46:543-51.

37 Meiqari L, Torre L, Gazmararian JA. Exploring the Impact of the New WIC Food Package on Low-Fat Milk Consumption Among WIC Recipients: A Pilot Study, *Journal of Health Care for the Poor & Underserved* 2015;26:712-25.

38 Nestor B, McKenzie J, Hasan N, et al. Client satisfaction with the nutrition education component of the California WIC program, *J Nutr Educ* 2001;33:83-94.

39 Odoms-Young A, Kong A, Schiffer LA, et al. Evaluating the initial impact of the revised Special Supplemental Nutrition Program for Women, Infants, and Children (WIC) food packages on dietary intake and home food availability in African-American and Hispanic families, *Public Health Nutr* 2014;17:83-93.

40 Pehrsson PR, Moser-Veillon PB, Sims LS, et al. Postpartum iron status in nonlactating participants and nonparticipants in the Special Supplemental Nutrition Program for Women, Infants, and Children, *Am J Clin Nutr* 2001;73:86-92.

41 Reyes NR, Klotz AA, Herring SJ. A qualitative study of motivators and barriers to healthy eating in pregnancy for low-income, overweight, African-American mothers, *Journal of the Academy of Nutrition & Dietetics* 2013;113:1175-81.

42 Rush D, Sloan NL, Leighton J, et al. The National WIC Evaluation: evaluation of the Special Supplemental Food Program for Women, Infants, and Children. V. Longitudinal study of pregnant women, *Am J Clin Nutr* 1988;48:439-83.

43 Rush D, Kurzon MR, Seaver WB, et al. The National WIC Evaluation: evaluation of the Special Supplemental Food Program for Women, Infants, and Children. VII. Study of food expenditures, *Am J Clin Nutr* 1988;48:512-9.

For peer review only

44 Swensen AR, Harnack LJ, Ross JA. Nutritional assessment of pregnant women enrolled in the Special Supplemental Program for Women, Infants, and Children (WIC), *J Am Diet Assoc* 2001;101:903-8.

45 Treiman K, Freimuth V, Damron D, et al. Attitudes and behaviors related to fruits and vegetables among low-income women in the WIC program, *J Nutr Educ Behav* 1996;28:149-56.

46 Watts V, Rockett H, Baer H, et al. Assessing diet quality in a population of low-income pregnant women: a comparison between Native Americans and whites, *Maternal & Child Health Journal* 2007;11:127-36.

47 Whaley SE, Ritchie LD, Spector P, et al. Revised WIC food package improves diets of WIC families, *Journal of Nutrition Education & Behavior* 2012;44:204-9.

48 Wunderlich SM, Hongu NK, Courter A, et al. Nutrient intake and nutritional status of low-income pregnant women, *Topics in Clinical Nutrition* 1996;12:66-74.

49 Thow AM, Downs S, Jan S. A systematic review of the effectiveness of food taxes and subsidies to improve diets: Understanding the recent evidence, *Nutr Rev* 2014;72:551-65.

50 Purnell JQ, Gernes R, Stein R, et al. A systematic review of financial incentives for dietary behavior change, *Journal of the Academy of Nutrition and Dietetics* 2014;114:1023-35.

51 An R. Effectiveness of subsidies in promoting healthy food purchases and consumption: a review of field experiments, *Public Health Nutr* 2013;16:1215-28.

52 Black AP, Brimblecombe J, Eyles H, et al. Food subsidy programs and the health and nutritional status of disadvantaged families in high income countries: a systematic review, *BMC Public Health* 2012;12:1099-122.

53 Bernstein DA. Essentials of psychology. Belmont, CA: Wadsworth 2011.

54 McLeod SA. Skinner - Operant Conditioning: www.simplypsychology.org/operant-conditioning.html. Date accessed: June 2016.

55 Abraham C, Michie S. A Taxonomy of Behavior Change Techniques Used in Interventions, *Health Psychol* 2008;27:379-87.

56 Oxford Dictionaries: http://www.oxforddictionaries.com/. Date accessed: June 2016.

57 UCL Centre for Behaviour Change. The Behaviour Change Wheel: http://www.behaviourchangewheel.com/. Date accessed: June 2016.

58 Hoddinott P, Craig LCA, Britten J, et al. A serial qualitative interview study of infant feeding experiences: Idealism meets realism, *BMJ Open* 2012;2.

For peer review only

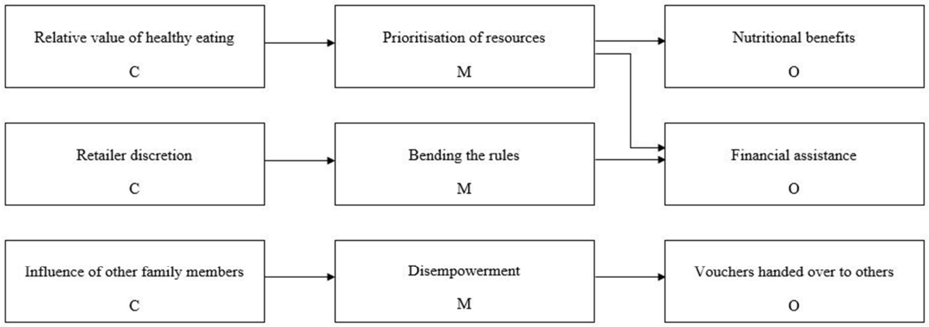

Figure 1. Summary of programme theories about how low-income pregnant women use Healthy Start vouchers

269x101mm (96 x 96 DPI)

For peer review only - http://bmjopen.bmj.com/site/about/guidelines.xhtml

List of items to be included when reporting a realist synthesis

**TITLE**			**Page**
1		In the title, identify the document as a realist synthesis or review	1
ABSTRACT			
2		While acknowledging publication requirements and house style, abstracts should ideally contain brief details of: the study's background, review question or objectives; search strategy; methods of selection, appraisal, analysis and synthesis of sources; main results; and implications for practice.	2
INTRODUCTION			
3	Rationale for review	Explain why the review is needed and what it is likely to contribute to existing understanding of the topic area.	4
4	Objectives and focus of review	State the objective(s) of the review and/or the review question(s). Define and provide a rationale for the focus of the review.	4-5
METHODS			
5	Changes in the review process	Any changes made to the review process that was initially planned should be briefly described and justified.	NA
6	Rationale for using realist synthesis	Explain why realist synthesis was considered the most appropriate method to use.	4
7	Scoping the literature	Describe and justify the initial process of exploratory scoping of the literature.	5-6
8	Searching processes	While considering specific requirements of the journal or other publication outlet, state and provide a rationale for how the iterative searching was done. Provide details on all the sources accessed for information in the review. Where searching in electronic databases has taken place, the details should include, for example, name of database, search terms, dates of coverage and date last searched. If individuals familiar with the relevant literature and/or topic area were contacted, indicate how they were identified and selected.	6
9	Selection and appraisal of documents	Explain how judgements were made about including and excluding data from documents, and justify these.	7-8
10	Data extraction	Describe and explain which data or information were extracted from the included documents and justify this selection.	8
11	Analysis and synthesis processes	Describe the analysis and synthesis processes in detail. This section should include information on the constructs analyzed and describe the analytic process.	9
RESULTS			
12	Document flow diagram	Provide details on the number of documents assessed for eligibility and included in the review with reasons for exclusion at each stage as well as an indication of their source of origin (for example, from searching databases, reference lists and so on).	9 and 21

For peer review only

TITLE			Page
		You may consider using the example templates (which are likely to need modification to suit the data) that are provided.	
13	Document characteristics	Provide information on the characteristics of the documents included in the review.	9-11
14	Main findings	Present the key findings with a specific focus on theory building and testing.	11-13
DISCUSSION			
15	Summary of findings	Summarize the main findings, taking into account the review's objective(s), research question(s), focus and intended audience(s).	13
16	Strengths, limitations and future research directions	Discuss both the strengths of the review and its limitations. These should include (but need not be restricted to) (a) consideration of all the steps in the review process and (b) comment on the overall strength of evidence supporting the explanatory insights which emerged.<br><br>The limitations identified may point to areas where further work is needed.	13-14
17	Comparison with existing literature	Where applicable, compare and contrast the review's findings with the existing literature (for example, other reviews) on the same topic.	14-15
18	Conclusion and recommendations	List the main implications of the findings and place these in the context of other relevant literature. If appropriate, offer recommendations for policy and practice.	15
19	Funding	Provide details of funding source (if any) for the review, the role played by the funder (if any) and any conflicts of interests of the reviewers.	16

Source of this checklist:

http://www.equator-network.org/reporting-guidelines/rameses-publication-standards-realist-syntheses/

http://www.ramesesproject.org/Standards_and_Training_materials.php

http://bmcmedicine.biomedcentral.com/articles/10.1186/1741-7015-11-21#Tab1

Page numbers refer to pdf document created during submission process.

For peer review only

PRISMA 2009 Flow Diagram

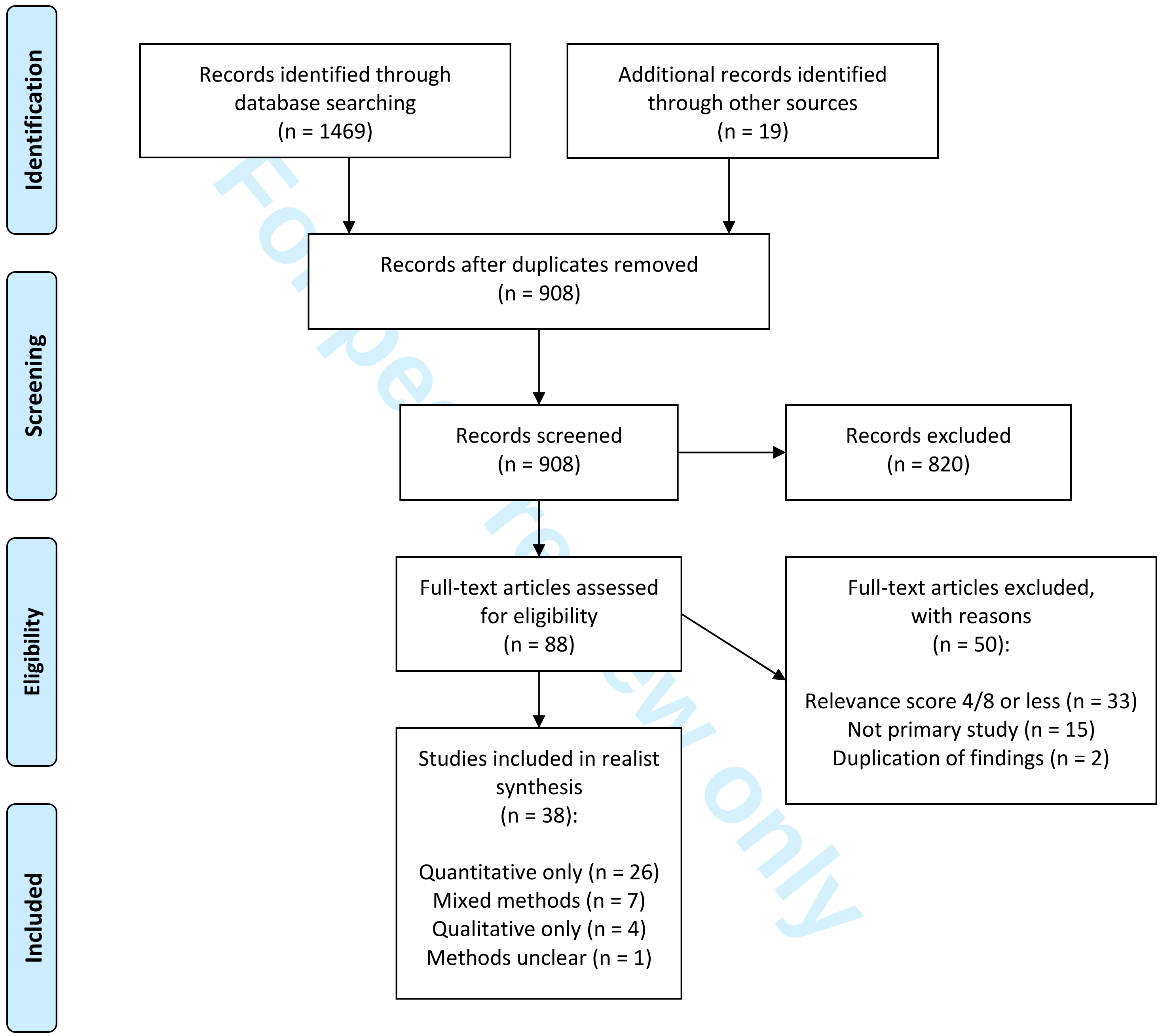

From: Moher D, Liberati A, Tetzlaff J, Altman DG, The PRISMA Group (2009). *P*referred *R*eporting *I*tems for *S*ystematic Reviews and *M*eta-*A*nalyses: The PRISMA Statement. PLoS Med 6(6): e1000097. doi:10.1371/journal.pmed1000097

For more information, visit www.prisma-statement.org.

For peer review only - http://bmjopen.bmj.com/site/about/guidelines.xhtml

BMJ Open

A realist review to explore how low-income pregnant women use food vouchers from the UK's Healthy Start programme

Journal:	*BMJ Open*
Manuscript ID	bmjopen-2016-013731.R1
Article Type:	Research
Date Submitted by the Author:	12-Oct-2016
Complete List of Authors:	Ohly, Heather; University of Central Lancashire, College of Health and Wellbeing<br>Crossland, Nicola; University of Central Lancashire, College of Health and Wellbeing<br>Lowe, Nicola; University of Central Lancashire, College of Health and Wellbeing<br>Dykes, Fiona; University of Central Lancashire, College of Health and Wellbeing<br>Hall-Moran, Victoria; University of Central Lancashire, College of Health and Wellbeing
**Primary Subject Heading**:	Public health
Secondary Subject Heading:	Nutrition and metabolism
Keywords:	pregnant, women, food, vouchers, realist, review

SCHOLARONE™
Manuscripts

For peer review only

Title of the article

A realist review to explore how low-income pregnant women use food vouchers from the UK's Healthy Start programme

Full name, postal address, email and telephone number of corresponding author:

Heather Ohly, Brook Building 215, College of Health and Wellbeing, University of Central Lancashire, Preston, UK

HOhly@uclan.ac.uk

07866 318014

Full name, department, institution, city and country of all co-authors:

Heather Ohly, College of Health and Wellbeing, University of Central Lancashire, Preston, UK

Nicola Crossland, College of Health and Wellbeing, University of Central Lancashire, Preston, UK

Nicola Lowe, College of Health and Wellbeing, University of Central Lancashire, Preston, UK

Fiona Dykes, College of Health and Wellbeing, University of Central Lancashire, Preston, UK

Victoria Hall Moran, College of Health and Wellbeing, University of Central Lancashire, Preston, UK

Word count, excluding title page, abstract, references, figures and tables:

5092 words

For peer review only

ABSTRACT

Objectives: To explore how low-income pregnant women use Healthy Start food vouchers, the potential impacts of the programme, which women might experience these impacts and why.

Design: A realist review.

Eligibility criteria for selecting studies: Primary or empirical studies (of any design) were included if they contributed relevant evidence or insights about how low-income women use food vouchers from the Healthy Start (UK) or the Special Supplemental Nutrition Program for Women, Infants and Children (US) programmes. The assessment of 'relevance' was deliberately broad to ensure that reviewers remained open to new ideas from a variety of sources of evidence.

Analysis: A combination of evidence synthesis and realist analysis techniques was used to modify, refine and substantiate programme theories, which were constructed as explanatory 'context – mechanism – outcome' (CMO) configurations.

Results: 38 primary studies were included in this review: four studies on Healthy Start and 34 studies on WIC. Two main outcome strands were identified: dietary improvements (intended) and financial assistance (unintended). Three evidence-informed programme theories were proposed to explain how aspects of context (and mechanisms) may generate these outcomes: the 'relative value' of healthy eating (prioritisation of resources); retailer discretion (pressure to 'bend the rules'); the influence of other family members (disempowerment).

Conclusions: This realist review suggests that some low-income pregnant women may use Healthy Start vouchers to increase their consumption of fruits and vegetables and plain cow's milk, whereas others may use them to reduce food expenditure and save money for other things. Further evidence is needed to understand which women are most (and least) likely to experience the intended outcome of dietary improvements.

Key words: pregnant, women, food, vouchers, realist, review

For peer review only

Strengths and limitations of this study:

- First study to articulate, develop and test programme theories about Healthy Start
- Inclusion of relevant studies from a similar programme in the US (WIC)
- Some evidence not transferable due to population and programme differences
- Insufficient evidence to link CMO configurations to sociodemographic and cultural characteristics

For peer review only

BACKGROUND

Healthy Start is the United Kingdom (UK) government's food voucher programme for low-income pregnant women and young children. Women are eligible for Healthy Start if they receive income-related benefits or child tax credit and an annual household income of £16,190 or less. Pregnant women aged under 18 are eligible regardless of their income. The weekly voucher values are: one voucher per week during pregnancy (£3.10); two vouchers per week for each baby under one year (£6.20) and one voucher per week for each child aged 1-4 years (£3.10). The vouchers can be exchanged for fruits and vegetables, plain cow's milk or infant formula. Retailers must be registered with the scheme to accept and claim payment for the vouchers. Healthy Start also offers free vitamins for eligible women and children, but there have been problems with uptake of vitamins.[1] Some areas offer free vitamins to all pregnant women and young children and the option of universalising Healthy Start vitamins remains under review (at the time of writing) by the Chief Medical Officer. Therefore, this review focused on the food voucher component of the programme and low-income pregnant women as the first beneficiaries.

Healthy Start aims to provide a "basic nutritional safety net" and to encourage "women and families to make positive nutritional choices affecting their longer term health" (page 4).[2] Therein lies an implicit programme assumption that women will use the vouchers to purchase target foods (ideally in greater quantities than they did before) leading to dietary improvements. The vouchers may have been conceived as a financial incentive for dietary improvements, although this was not explicit in policy documentation. Since the introduction of Healthy Start in 2006, there has been no robust evaluation of its impact on nutritional outcomes – despite recommendations published in an early scoping review.[3] This review was undertaken to explore how low-income pregnant women use Healthy Start food vouchers, the potential impacts of the programme, which women might experience these impacts and why.

The realist approach was adopted because it is based on the understanding that different individuals or groups of individuals are likely to respond to any programme (or intervention) in different ways. It is a theory-driven approach that seeks to explore outcome patterns (or demi-regularities) and offer plausible explanations for how and why they occur. The purpose of realist synthesis is to "articulate underlying programme theories and then to interrogate the existing evidence to find out whether and where these theories are pertinent and productive"

For peer review only

(page 74).[4] The stages of conducting a realist review tend to be iterative and overlapping – a gradual process of developing, testing and refining programme theories. Evidence may be obtained from studies of the programme itself, or more widely from similar programmes that are thought to work in similar ways. Reviewers may adapt and modify existing theoretical frameworks or 'middle-range' theories to help develop their own explanations. The unit of analysis is the programme theory (rather than the specific programme) and this can be considered at different levels of abstraction.

Programme theories are often constructed as 'context – mechanism – outcome' (CMO) configurations, and evidence is sought to substantiate the causal linkages. Context refers to the pre-existing conditions into which the programme is introduced, and there are four layers of context: individual, interpersonal, institutional and infrastructural.[4] In certain contexts, the programme may activate mechanisms, defined as the reasoning and reactions of individuals in response to the resources offered by the programme. A core principle of realism is that mechanisms generate outcomes – they are not a direct result of the programme.

For peer review only

This realist review aimed to explore the following questions:

1. Do low-income pregnant women use Healthy Start vouchers to improve their diets?
2. What are the alternative or unintended outcomes of the programme?
3. What are the underlying mechanisms and how do variations in context influence (enable or constrain) these mechanisms?

A preliminary search confirmed the paucity of empirical studies on Healthy Start and we felt that additional sources of empirical evidence would be needed to explore these research questions. The most obvious source of potentially relevant evidence was the Special Supplemental Nutrition Program for Women, Infants and Children (WIC) in the United States (US). WIC is the only other national food subsidy programme for low-income women of childbearing age, including pregnant and postpartum women, and young children. It was first introduced in 1972 and revised in 2009 to reflect current dietary guidelines.[5] The WIC food package for pregnant women is different to Healthy Start, with 'maximum monthly allowances' for a range of foods (reduced-fat milk, whole grain cereals, eggs and pulses) and 'cash value vouchers' for fruits and vegetables. The other main programme difference is mandatory WIC nutrition education for all beneficiaries. There are also many contextual

differences between the US and the UK, such as socio-demographic, cultural, geographical and political characteristics. Despite these differences, there are also likely to be similarities in terms of how low-income women respond to the programme (i.e. mechanisms).

METHODS

Study protocol and ethical approval

The protocol for this realist review was registered with the International Prospective Register of Systematic Reviews (PROSPERO 2014: CRD42014015050). There were no changes to the review process since this protocol was published. Ethical approval was obtained from the University of Central Lancashire Science, Technology, Engineering, Medicine and Health (STEMH) Ethics Committee in May 2015.

Programme theory development

This review is about a programme that already exists, with implicit theories and assumptions about how it works and what effects it may have on beneficiaries.[4] Therefore, we used a 'bottom up' approach to theory development in this review, as described by Shearn and Allmark (Realist Research Seminar Series, Sheffield Hallam University, 2016). In other words, we developed theories using information about the Healthy Start food voucher programme, rather than theorising at a more abstract level. Candidate theories (or initial, untested theories) were identified and prioritised using information derived from academic and grey literature on Healthy Start, an intervention mapping exercise, existing knowledge, creative thinking, consultations with stakeholders (in person and by email) and discussions among the review team. The stakeholder group included six midwives, two academics and two public health practitioners, who all shared first hand experiences and insights about how the Healthy Start programme works in practice.

Search strategy

Separate searches were conducted for Healthy Start and WIC:

Healthy Start – Studies were identified through manual, purposive, snowball and citation searches (January to March 2015). The search terms used initially were 'Healthy Start' and 'UK' because there is another programme called Healthy Start in the US, which aims to prevent infant mortality. More precise search terms were not needed due to the paucity of empirical studies and familiarity with the literature.

For peer review only

WIC – A broad search strategy was devised in collaboration with an Information Specialist in the Collaboration for Leadership in Applied Health Research and Care (CLAHRC) North West Coast. This strategy was adapted and run in six electronic databases in September 2015: MEDLINE, EMBASE, CINAHL, Open Grey, ETHOS and PubMed. Table 1 shows the search terms used in MEDLINE. No date or language restrictions were used. Reference lists of included studies were checked for additional studies. An online list of WIC studies was also checked for additional studies.[6]

Table 1. Search strategy used in MEDLINE to identify WIC studies

#	Search terms	Results
1	WIC.tw.	1008
2	(nutrition or food or voucher or "nutrition program").tw.	377002
3	1 and 2	599
4	(Special* adj4 Supplement* adj4 Nutrit* adj4 Program* adj4 Women* adj4 Infant* adj4 Child*).tw.	415
5	3 or 4	688

Inclusion criteria

Primary or empirical studies (of any design) were included if they contributed relevant evidence or insights about how low-income women use food vouchers from the Healthy Start (UK) or WIC (US) programmes. An assessment of 'relevance' is essential in realist synthesis, to ensure that all included studies contribute to theory development, refinement and testing.[4, 7] In this review, the interpretation of 'relevance' was deliberately broad to ensure that reviewers remained open to new ideas from a variety of sources of evidence. A bespoke system was used to maintain a consistent and transparent approach. Table 2 shows the questions used to assess relevance. These questions were developed by the review team and finalised towards the end of the theory development stage, to ensure they reflected the candidate theories we wanted to test. Studies that scored 5/8 or more (based on the total number of yes answers) were included.

Table 2. Questions used to assess the relevance of primary studies

#	Question:
1	Do the research questions or study aims refer to Healthy Start or WIC?

For peer review only

2	Does the study focus on the food voucher (CVV or food package for WIC) component of the programme?
3	Does the study focus on beneficiaries (women who were receiving the vouchers) rather than eligibility status (women who were eligible to receive the vouchers)?
4	Does the sample include pregnant women?
5	If the sample does not include pregnant women, could some of the findings be generalizable to pregnant women?
6	Does the study report women's food or nutrient intakes (measured or perceived)?
7	Does the study provide any insights about how food vouchers are used?
8	Does the study provide any insights about which women may benefit most/least and why?

Study selection

Results from the WIC database searches were uploaded into RefWorks (web version; ProQuest; Michigan, US) and screened using titles and abstracts. Studies that appeared to meet the inclusion criteria were obtained as full text articles. Studies for which insufficient information was available to determine relevance were also obtained as full text articles. The full text screening process was fully documented using Microsoft Excel 2013 (version 15.0.4815.1001; Microsoft Corporation), including assessments of relevance and reasons for exclusions. The same criteria were applied to studies of Healthy Start. Study selection was completed by the lead reviewer (HO) and double checked by a second reviewer (VM). Any disagreements were resolved by discussion.

Data extraction

Quantitative data on women's nutritional outcomes were extracted using bespoke tables in Microsoft Word 2013 (version 15.0.4815.1001; Microsoft Corporation). Other non-relevant data were not extracted. Qualitative data, textual descriptions of findings, and author interpretations were extracted using MAXQDA 11 (version 11; VERBI Software GmbH; Berlin). A coding system was created with three main headings: context, mechanisms and outcomes. Subheadings were added deductively (based on candidate theories) and inductively (as new themes emerged from the data). Data extraction was completed by the lead reviewer (HO) and a sample was double checked by a second reviewer (NC).

For peer review only

Quality appraisal

Studies were not formally appraised at the data extraction stage, as would be the case in traditional systematic reviews. Instead, an assessment of 'rigour' was used to judge the credibility and trustworthiness of the evidence as it was integrated into the analysis and synthesis.[4, 7] This assessment was not scored because weaker studies were still included, but it meant that methodological limitations were acknowledged and study findings were not over-interpreted. Table 3 shows the questions used to assess rigour. Quality appraisal was completed by the lead reviewer (HO) and double checked by second reviewers (NL/VM).

Table 3. Questions used to assess the rigour of primary studies

#	Question:
1	Are the study methods clearly reported (including study design, recruitment, data collection and analysis)?
2	Are the study methods appropriate to answer the research questions?
3	Are the sample characteristics reported to enable judgements about generalisability?
4	Are the study findings and conclusions supported by raw data?
5	Are the study limitations acknowledged and clearly reported?

Analysis and synthesis

This process involved gradual and iterative theory development, whereby evidence from primary studies was used to modify, refine and substantiate programme theories about how low-income pregnant women use Healthy Start vouchers, in what circumstances, and why. Theories were constructed as explanatory 'context – mechanism – outcome' (CMO) configurations, usually by starting with the outcome and working backwards to determine "what caused it (the mechanism) and under what contexts was the mechanism triggered".[8] The main focus of the analysis was searching for evidence to support and refute the proposed causal linkages between context, mechanisms and outcomes. A combination of evidence synthesis and realist analysis techniques was used:

1. Narrative synthesis of quantitative data on women's nutritional outcomes; meta-analysis was not appropriate due to heterogeneity of study designs and data collection methods (and was beyond the scope of this review).

For peer review only

2. Thematic synthesis of qualitative data, by creating codes and themes (as described under data extraction) and then 'going beyond' the interpretations of the original studies to generate new understandings or hypotheses.[9]
3. Creative theorising or 'retroduction' by the lead reviewer (HO) in collaboration with the review team and the stakeholder group. This involved in-depth reflection and discussions (throughout the review) about the underlying causes of outcome patterns, at the level of generative mechanisms and explanatory context. The data from included studies did not always provide such in-depth insights and explanations. Where individual extracts of data only supported part of the CMO configuration, it was necessary to make logical inferences about the complete causal pathways and explanations.[10]

RESULTS

Search results and study characteristics

A total of 908 records were identified through the two separate searches. After screening titles and abstracts, 88 records were obtained in full text format. Fifty full text articles were excluded based on the assessment of relevance (n=33) or because they were not primary studies (n=15) or the findings were duplicated (n=2). Therefore, 38 primary studies were included in this review: four UK studies on Healthy Start[11-14] and 34 US studies on WIC[15-48] (see PRISMA Flow Diagram and online supplementary file 1).

Candidate programme theories

We developed candidate theories about how low-income pregnant women might experience the Healthy Start programme. These theories were not always fully formed as CMO configurations, but they were initial ideas and hypotheses about how the programme works and what factors might influence women's experiences and outcomes at each stage. The stages we considered were: access to the programme starting at the first trimester antenatal appointment (e.g. issues around clear communication and understanding of eligibility criteria and entitlement); the application process (e.g. barriers and facilitators to successfully receiving the vouchers); whether and where women use the food vouchers (e.g. issues around convenience and stigmatisation); and how women use the food vouchers (e.g. to buy more of the target foods or to save money). We decided to prioritise the last stage of the programme pathway: once an eligible pregnant woman has received the food vouchers, how does she use them and why? This decision reflected the research priorities identified from the literature,

For peer review only

the interests of the research team, and the time and resources available to conduct this review. The following sections describe how we tested and refined our candidate programme theories.

Identification of outcomes and supporting evidence

Two main outcome strands emerged during the theory development stage and were further substantiated using evidence from primary studies:

1. Women use vouchers to increase consumption of target foods – dietary improvements.
2. Women use vouchers to reduce food expenditure – financial assistance.

For the purposes of this review, we have assumed that strand 1 is the intended outcome of the programme and strand 2 is an unintended outcome. This was not explicit in policy documentation, but there were references to dietary improvements, which were thought to be achieved by enabling low-income women to access healthier foods and encouraging positive nutritional choices.[2] The included studies provided support for both outcome strands. The next section provides an overview of the available evidence on women's outcomes (intended and unintended) and highlights the relative contribution of evidence from Healthy Start and WIC studies. It also helps to illustrate how we worked backwards from outcomes to identify generative mechanisms and related aspects of context.

A total of 25 studies reported women's nutritional outcomes: three studies on Healthy Start and 22 studies on WIC. The Healthy Start studies reported perceived outcomes only; some women said they consumed more cow's milk, fruits and vegetables after receiving Healthy Start vouchers,[11, 12, 14] whereas other women said the vouchers "freed up money to do other things" and "helped them to manage better financially" (page 59).[12] The WIC studies were published between 1981 and 2015, but the most useful data was extracted from two studies comparing women's diets before and after the 2009 WIC revisions, when the 'cash value vouchers' for fruits and vegetables were introduced (there was no allowance for fresh fruits and vegetables before 2009). A longitudinal study of African American and Hispanic women from WIC clinics in Chicago (n=273) found significant dietary improvements for Hispanic mothers, who reported consuming more fruit, more reduced fat milk less whole milk and less saturated fat (all $p<0.05$).[39] African American mothers reported consuming less whole milk ($p=0.02$) but no other changes were statistically significant.[39] There were no sustained dietary improvements in either group compared to baseline at 18 months.[36] A

For peer review only - http://bmjopen.bmj.com/site/about/guidelines.xhtml

cross-sectional study comparing two random samples of WIC participants in California (both 80% Hispanic) found that women assessed six months after the changes (n=2996) reported consuming significantly more whole grains, reduced-fat milk and vegetables, and less whole milk, compared to women assessed before the changes (n=3004) (all p<0.001).[47]

Five studies reported electronic sales data from WIC retailers (one large supermarket chain) in New England, which showed that women's purchasing patterns shifted towards items provided in the WIC package after the 2009 revisions – fruits and vegetables, reduced-fat milk, brown rice, whole grain cereals and bread replaced less nutritious options.[16-20] One study (n=2137) showed that, while total spending on fruits and vegetables increased between 2009 and 2010 (p<0.001), up to 13% fewer purchases were made using non-WIC funds.[20] This implies that some women 'substituted' the method of payment, rather than using WIC to increase the amount of fruits and vegetables purchased. None of these studies reported sample characteristics such as ethnicity. Finally, a mixed methods study of Hispanic and African American pregnant women (n=313) found that two thirds of women reported using WIC vouchers to reduce food expenditure.[31] The money they saved was used to purchase items for the unborn baby, other foods, and for bills and emergencies.

These findings suggest that food vouchers may lead to dietary improvements for some, but not all women. This may be because some women use the vouchers to pay for foods they would previously have bought using cash. The WIC studies described above were not representative of ethnic groups in the UK, and the samples included mothers as well as pregnant women. However, these studies provide much needed evidence on the potential impact of food vouchers for low-income women, which was not available from the Healthy Start literature alone.

Evidence-informed programme theories

This section presents three evidence-informed programme theories, which help to explain why different women receive the same Healthy Start vouchers and yet experience different outcomes, due to variations in context and mechanisms. Figure 1 illustrates the key aspects of context, mechanisms and outcomes identified and the proposed causal pathways linking them together. These causal pathways are explained as CMO configurations and illustrated using quotations from included studies under each theory. Although low income is clearly an important aspect of context, we have not included it in our programme theories because it

For peer review only

applies to all women who receive Healthy Start vouchers (apart from under 18s). As the programme theories explain, some women may achieve dietary improvements despite low income, and other women may not – this divergence depends on other aspects of context.

1. Prioritisation of resources

Women living on low incomes must constantly prioritise how they spend their money. Food vouchers may be considered as one part of the household resources and decisions must be made about how best to use the vouchers. A key aspect of individual level context is the 'relative value' of healthy eating (compared to other things women value), which can lead women to prioritise in different ways. Context is not static and women's values may change over time, so some women may fluctuate between the mechanisms (ways of prioritising) outlined in these two contrasting CMO configurations.

Women who value healthy eating and aspire to eat well during pregnancy [context][45] are more likely to perceive Healthy Start vouchers as an opportunity to achieve health benefits for themselves and their unborn baby [mechanism].[11] The vouchers alleviate the financial barrier associated with healthy eating and make healthy foods seem more affordable [mechanism].[11] Therefore, women who value healthy eating are more likely to prioritise healthy eating [mechanism] and use Healthy Start vouchers to increase consumption of target foods – fruits and vegetables or cow's milk [outcome].[11]

> "I have them at Asda when I do my shop, and I think how many vouchers I've got and I buy the veg that I have the vouchers for. I suppose if I didn't have the vouchers, I would just pick out the little things. I don't think if I didn't have the vouchers I'd buy half as much, no." (Mother, UK; page 50)[11]

Alternatively, women may value healthy eating less than other things they want or need to spend money on, which are considered more important or urgent [context].[11, 12] They are more likely to perceive Healthy Start vouchers as a way to save money, which can be redirected and prioritised in other ways [mechanism].[11, 12] These women are more likely to use Healthy Start vouchers to deduct money from the shopping bill, with no increase in consumption of target foods [outcome].[11, 12, 31]

> "Women are often in a dilemma about whether they should or shouldn't eat healthy foods because something else is needed more. Their own health and maybe the health

For peer review only

> of their younger children are on the back burner because something else is more pressing.” (Midwife, UK; page 35)[11]

2. Bending the rules

The Healthy Start voucher exchange system relies on registered retailers to verify (visually) at the checkout that women have selected appropriate amounts of appropriate items – fruits and vegetables, plain cow’s milk or infant formula. The vouchers are processed by swiping a barcode that subtracts the voucher value (£3.10) from the total. They are not electronically matched to specific food items. There is a reminder printed on each voucher about which foods may be purchased, along with a warning about prosecution, but the evidence suggests that some customers and retailers appear to disregard this information.

Retailers who are registered to accept Healthy Start vouchers have some discretion over how vigilantly they check what vouchers are spent on [context].[11, 13] Women may put pressure on retailers to ‘bend the rules’ or make exceptions [mechanism].[11, 13-15, 30] Some retailers may decide to ‘turn a blind eye’ because they feel duty bound to help families in whatever ways they can[mechanism][11] or because they prefer to avoid conflict [mechanisms].[14] This enables women to exchange the vouchers for alternative food or non-food items [outcomes].[11, 14]

> “But you have to realise that I get people coming in here, they are buying £1 pound of electricity every day. £1. That must run out after an hour. How do they live? And in the winter, it really does get very cold and they come in and ask me if they can use the voucher for electricity. What can I do? I can’t see them living in the flat with young children, with no heating, it’s so cold. So I do let them do that. They come in and show me their empty wallet and I have to believe them and I do sell gas and electricity for the voucher. You can report that back. I don’t care, what can I do?” (Retailer, UK; page 69)[11]

3. Disempowerment

Pregnant women (and later their young children) are the intended beneficiaries of Healthy Start, and the vouchers are addressed to them, but some women may not be empowered to use the vouchers themselves. The name of the beneficiary is not printed on the vouchers and there is nothing to stop other people from using them. Regardless of what is bought with the

For peer review only

vouchers, and who benefits, this would surely be considered an unintended outcome of the programme.

Women may not be empowered to make decisions about household resources or food shopping, such as pregnant teenagers who live with their parents [context],[11, 45] or women who live in large, multi-generational households [context].[41] Women who are disempowered are more likely to hand over their Healthy Start vouchers to other family members [mechanism][41] who then decide how they are used [outcome].[41]

> "She (mom) makes most of the decisions. We get the same thing every time we go shopping." (African-American mother living in multi-generational household, US; page 5)[41]

DISCUSSION

Statement of principal findings

Low-income pregnant women may use food vouchers from the Healthy Start programme in different ways, for different reasons, leading to different outcomes. The two main outcome strands identified in this realist review were dietary improvements (intended) and financial assistance (unintended). Three evidence-informed programme theories were developed and refined to explain how aspects of context (and mechanisms) may generate these outcomes: the 'relative value' of healthy eating (prioritisation of resources); retailer discretion (pressure to 'bend the rules'); the influence of other family members (disempowerment).

Strengths of this review

This was the first study to use a realist, theory-driven approach to investigate how low-income pregnant women use Healthy Start vouchers, in what circumstances, and why. The inclusion of relevant studies from a similar programme in the US (WIC) provided insights and explanations beyond what was available from the Healthy Start literature. This was the first time that researchers have attempted to articulate, develop and test programme theories about how Healthy Start works. Our findings suggest that Healthy Start vouchers are not always used to achieve dietary improvements. Some pregnant women may need to receive more support to increase the perceived value/importance of healthy eating. Modifications to the voucher verification system would help to prevent the vouchers being used by other

For peer review only

people and exchanged for alternative items. We hope this review will stimulate discussions about future evaluation needs and programme development.

Limitations of this review

We were aware from the outset that some of the evidence from WIC studies would not be transferable to Healthy Start due to population and programme differences. Only theories that were adequately supported by the existing evidence were presented. Further evidence from the UK is needed to develop some of the other candidate theories we identified. This review focused on women's outcomes from the programme, and the aspects of context explored in our theories were individual (women's values and perceptions) and interpersonal (interactions with retailers and other family members).[4] We did not find sufficient evidence to link our CMO configurations with sociodemographic and cultural characteristics, such as which groups of women are more/less likely to value healthy eating. Future reviews in this area could include wider evidence on voucher programmes to provide insights about how the mechanisms we have identified might operate in different contexts and different programmes. This was beyond the scope of this review due to time and resource limitations. Finally, we did not explore theories relating to Healthy Start vitamins, women's decisions about infant feeding or children's outcomes. These would all be worthy areas for realist investigations.

Comparisons with existing literature and theories

Healthy Start may have been conceived as (or assumed to be) a financial incentive for dietary improvements. Our programme theories suggest that the vouchers may provide this kind of incentive for some women but not for others. Previous systematic reviews have shown that financial incentives (including taxes, subsidies, vouchers and cash payments) are associated with short term dietary improvements.[49-52] However, no studies of Healthy Start met the inclusion criteria for these reviews and the impact of the programme on women's nutritional outcomes remains unclear. There is also some uncertainty around the definition and function of an incentive in relation to behaviour change, and whether Healthy Start fits into this category of interventions. Incentive Theory states that behaviour is motivated by a desire for rewards or positive reinforcements.[53] Skinner's Operant Conditioning Theory states that behaviours that are reinforced tend to be repeated.[54] A recent taxonomy of behaviour change techniques defined 'incentives' as rewarding and contingent on behaviour change.[55] Healthy Start does not fit this definition, since the vouchers are provided to all eligible women regardless of how they are subsequently used. However, the dictionary

For peer review only

definition of an incentive is broader: "a thing that motivates or encourages someone to do something",[56] which encompasses opportunities for behavior change as well as rewards for behavior change.

Our programme theories suggest that individually held values may determine whether the vouchers are perceived as an opportunity to achieve health benefits or a way to save money. According to the Behaviour Change Wheel, 'opportunity' is one of the key ingredients for behaviour change.[57] Food vouchers provide a physical opportunity (increased monetary resources) for women to buy more of the target foods, but they are only likely to do this if they value healthy eating. The concept of 'value' is also important in Incentive Theory in terms of how effective an incentive might be for different individuals in different situations.[53] Our programme theories also suggest that 'competing values' may determine whether women perceive, and take advantage of, the opportunity to improve their diets. Hoddinott et al. used the constructs of 'idealism' and 'realism' to explain how competing values influence women's infant feeding decisions and practices.[58] They found that immediate concerns (such as pain, anxiety and lack of sleep) often outweighed the long term health benefits associated with breastfeeding. These competing values were said to be more complex for disadvantaged families who may be dealing with greater adversity. Similarly, low-income women may use their Healthy Start vouchers to save money because they consider other things to be more urgent or important than healthy eating. For these women, it might be unrealistic to anticipate that Healthy Start vouchers alone would lead to dietary improvements.

CONCLUSION

This realist review suggests that some low-income pregnant women use Healthy Start vouchers to increase their consumption of fruits and vegetables and plain cow's milk (intended outcome: dietary improvements) whereas other women use them to reduce food expenditure and save money for other things (unintended outcome: financial assistance). We have identified some aspects of context (the 'relative value' of healthy eating, retailer discretion and the influence of other family members) and mechanisms (prioritisation of resources, pressure to 'bend the rules' and disempowerment) that are likely to be important in determining these outcomes. Further evidence is needed to understand which women are most (and least) likely to experience the intended outcome of dietary improvements. Targeted

For peer review only

support may be needed to help some low-income pregnant women to use Healthy Start vouchers to improve their diets during pregnancy.

For peer review only

STATEMENTS

Acknowledgements: The authors would like to thank the stakeholder group for their contributions to theory development, and Eleanor Kotas (Information Specialist) for her assistance with the electronic database searches.

Contributors: HO was the lead reviewer, while other authors completed the second reviewer tasks (as indicated in the Methods). All authors contributed to the development of programme theories through regular discussions. HO drafted the initial manuscript and all authors contributed to the final manuscript.

Funding: This work was supported by a PhD Studentship (2014-2017) from the Collaboration for Leadership in Applied Health Research and Care North West Coast (CLAHRC NWC).

Declaration: The views expressed are those of the authors and not necessarily those of the DWP, NHS, NIHR or the Department of Health.

Competing interests: None declared.

Data sharing statement: No additional data are available.

For peer review only

REFERENCES

1 McFadden A, Green JM, McLeish J, et al. Healthy Start vitamins – a missed opportunity: findings of a multimethod study, *BMJ Open* 2015;5.

2 Department of Health. Healthy Start: Equality impact assessment. London, 2010.

3 Dyson L, Renfrew MJ, Jenkins R, et al. Approaches to evaluating Healthy Start – a scoping review. Public Health Research Consortium, 2007.

4 Pawson R. Evidence-based policy: a realist perspective. London: Sage 2006:196.

5 Institute of Medicine of the National Academies. WIC Food Packages: Time for a Change. Institute of Medicine, 2005.

6 United States Department of Agriculture: Food and Nutrition Service. WIC Studies: http://www.fns.usda.gov/ops/wic-studies. Date accessed: February 2015.

7 The RAMESES Project. Quality Standards for Realist Synthesis (for researchers and peer-reviewers): http://www.ramesesproject.org/media/RS_qual_standards_researchers.pdf. Date accessed: November 2014.

8 Wong G. Special Invited Editorial: Getting Started With Realist Research, *International Journal of Qualitative Methods* 2015;14.

9 Thomas J, Harden A. Methods for the thematic synthesis of qualitative research in systematic reviews, *BMC Medical Research Methodology* 2008;8:45.

10 Ford JA, Wong G, Jones AP, et al. Access to primary care for socioeconomically disadvantaged older people in rural areas: a realist review, *BMJ Open* 2016;6.

11 Lucas PJ, Jessiman T, Cameron A, et al. Healthy Start Vouchers Study: The Views and Experiences of Parents, Professionals and Small Retailers in England. Bristol: University of Bristol, 2013.

12 McFadden A, Fox-Rushby J, Green JM, et al. Healthy Start: Understanding the use of vouchers and vitamins. Dundee: University of Dundee, 2013.

13 Department of Health. Healthy Start: Retailer research summary. London, 2012.

14 Hills D, Child C, Junge K, Wilkinson E, Sullivan F. Healthy Start: Rapid evaluation of early impact on beneficiaries, health professionals, retailers and contractors. London: Symbia/Tavistock Institute, 2006.

15 Andreyeva T, Middleton AE, Long MW, et al. Food retailer practices, attitudes and beliefs about the supply of healthy foods, *Public Health Nutr* 2011;14:1024-31.

16 Andreyeva T, Luedicke J, Henderson KE, et al. Grocery store beverage choices by participants in federal food assistance and nutrition programs, *Am J Prev Med* 2012;43:411-8.

For peer review only

17 Andreyeva T, Luedicke J, Tripp AS, et al. Effects of reduced juice allowances in food packages for the women, infants, and children program, *Pediatrics* 2013;131:919-27.

18 Andreyeva T, Luedicke J. Federal food package revisions: effects on purchases of whole-grain products, *Am J Prev Med* 2013;45:422-9.

19 Andreyeva T, Luedicke J, Henderson KE, et al. The positive effects of the revised milk and cheese allowances in the special supplemental nutrition program for women, infants, and children, *Journal of the Academy of Nutrition & Dietetics* 2014;114:622-30.

20 Andreyeva T, Luedicke J. Incentivizing fruit and vegetable purchases among participants in the Special Supplemental Nutrition Program for Women, Infants, and Children, *Public Health Nutr* 2015;18:33-41.

21 Ayala GX, Laska MN, Zenk SN, et al. Stocking characteristics and perceived increases in sales among small food store managers/owners associated with the introduction of new food products approved by the Special Supplemental Nutrition Program for Women, Infants, and Children, *Public Health Nutr* 2012;15:1771-9.

22 Bailey LB, O'Farrell-Ray B, Mahan CS, et al. Vitamin B6, iron and folacin status of pregnant women, *Nutr Res* 1983;3:783-93.

23 Bertmann FM, Barroso C, Ohri-Vachaspati P, et al. Women, infants, and children cash value voucher (CVV) use in Arizona: a qualitative exploration of barriers and strategies related to fruit and vegetable purchases, *Journal of Nutrition Education & Behavior* 2014;46:S53-8.

24 Black MM, Hurley KM, Oberlander SE, et al. Participants' comments on changes in the revised special supplemental nutrition program for women, infants, and children food packages: the Maryland food preference study, *J Am Diet Assoc* 2009;109:116-23.

25 Chen DY, Gazmararian JA. Impact of personal preference and motivation on fruit and vegetable consumption of WIC-participating mothers and children in Atlanta, GA, *Journal of Nutrition Education & Behavior* 2014;46:62-7.

26 Endres J, Dunning S, Poon SW, et al. Older pregnant women and adolescents: nutrition data after enrollment in WIC, *J Am Diet Assoc* 1987;87:1011.

27 Endres JM, Sawicki M, Casper JA. Dietary assessment of pregnant women in a supplemental food program. *J Am Diet Assoc* 1981;79:121-6.

28 Ettienne-Gittens R, McKyer ELJ, Odum M, et al. Rural versus Urban Texas WIC participants' fruit and vegetable consumption, *Am J Health Behav* 2013;37:130-40.

29 Farrior ES, Ruwe CH. Women, infants and children program. Prenatal participation and dietary intakes, *Nutr Res* 1987;7:451-549.

30 Gittelsohn J, Laska MN, Andreyeva T, et al. Small retailer perspectives of the 2009 women, infants and children program food package changes, *Am J Health Behav* 2012;36:655-65.

For peer review only

31 Herman DR, Harrison GG, Afifi AA, et al. The Effect of the WIC Program on Food Security Status of Pregnant, First-Time Participants, *Family Economics & Nutrition Review* 2004;16:21.

32 Herman DR, Harrison GG, Jenks E. Choices made by low-income women provided with an economic supplement for fresh fruit and vegetable purchase, *J Am Diet Assoc* 2006;106:740-4.

33 Herman DR, Harrison GG, Afifi AA, et al. Effect of a targeted subsidy on intake of fruits and vegetables among low-income women in the Special Supplemental Nutrition Program for Women, Infants, and Children, *Am J Public Health* 2008;98:98-105.

34 Horswill LJ, Yap C. Consumption of foods from the WIC food packages of Chinese prenatal patients on the US west coast, *J Am Diet Assoc* 1999;99:1549-53.

35 Kong A, Odoms-Young A, Schiffer LA, et al. Racial/ethnic differences in dietary intake among WIC families prior to food package revisions, *Journal of Nutrition Education & Behavior* 2013;45:39-46.

36 Kong A, Odoms-Young A, Schiffer LA, et al. The 18-month impact of special supplemental nutrition program for women, infants, and children food package revisions on diets of recipient families, *Am J Prev Med* 2014;46:543-51.

37 Meiqari L, Torre L, Gazmararian JA. Exploring the Impact of the New WIC Food Package on Low-Fat Milk Consumption Among WIC Recipients: A Pilot Study, *Journal of Health Care for the Poor & Underserved* 2015;26:712-25.

38 Nestor B, McKenzie J, Hasan N, et al. Client satisfaction with the nutrition education component of the California WIC program, *J Nutr Educ* 2001;33:83-94.

39 Odoms-Young A, Kong A, Schiffer LA, et al. Evaluating the initial impact of the revised Special Supplemental Nutrition Program for Women, Infants, and Children (WIC) food packages on dietary intake and home food availability in African-American and Hispanic families, *Public Health Nutr* 2014;17:83-93.

40 Pehrsson PR, Moser-Veillon PB, Sims LS, et al. Postpartum iron status in nonlactating participants and nonparticipants in the Special Supplemental Nutrition Program for Women, Infants, and Children, *Am J Clin Nutr* 2001;73:86-92.

41 Reyes NR, Klotz AA, Herring SJ. A qualitative study of motivators and barriers to healthy eating in pregnancy for low-income, overweight, African-American mothers, *Journal of the Academy of Nutrition & Dietetics* 2013;113:1175-81.

42 Rush D, Sloan NL, Leighton J, et al. The National WIC Evaluation: evaluation of the Special Supplemental Food Program for Women, Infants, and Children. V. Longitudinal study of pregnant women, *Am J Clin Nutr* 1988;48:439-83.

43 Rush D, Kurzon MR, Seaver WB, et al. The National WIC Evaluation: evaluation of the Special Supplemental Food Program for Women, Infants, and Children. VII. Study of food expenditures, *Am J Clin Nutr* 1988;48:512-9.

For peer review only

44 Swensen AR, Harnack LJ, Ross JA. Nutritional assessment of pregnant women enrolled in the Special Supplemental Program for Women, Infants, and Children (WIC), *J Am Diet Assoc* 2001;101:903-8.

45 Treiman K, Freimuth V, Damron D, et al. Attitudes and behaviors related to fruits and vegetables among low-income women in the WIC program, *J Nutr Educ Behav* 1996;28:149-56.

46 Watts V, Rockett H, Baer H, et al. Assessing diet quality in a population of low-income pregnant women: a comparison between Native Americans and whites, *Maternal & Child Health Journal* 2007;11:127-36.

47 Whaley SE, Ritchie LD, Spector P, et al. Revised WIC food package improves diets of WIC families, *Journal of Nutrition Education & Behavior* 2012;44:204-9.

48 Wunderlich SM, Hongu NK, Courter A, et al. Nutrient intake and nutritional status of low-income pregnant women, *Topics in Clinical Nutrition* 1996;12:66-74.

49 Thow AM, Downs S, Jan S. A systematic review of the effectiveness of food taxes and subsidies to improve diets: Understanding the recent evidence, *Nutr Rev* 2014;72:551-65.

50 Purnell JQ, Gernes R, Stein R, et al. A systematic review of financial incentives for dietary behavior change, *Journal of the Academy of Nutrition and Dietetics* 2014;114:1023-35.

51 An R. Effectiveness of subsidies in promoting healthy food purchases and consumption: a review of field experiments, *Public Health Nutr* 2013;16:1215-28.

52 Black AP, Brimblecombe J, Eyles H, et al. Food subsidy programs and the health and nutritional status of disadvantaged families in high income countries: a systematic review, *BMC Public Health* 2012;12:1099-122.

53 Bernstein DA. Essentials of psychology. Belmont, CA: Wadsworth 2011.

54 McLeod SA. Skinner - Operant Conditioning: www.simplypsychology.org/operant-conditioning.html. Date accessed: June 2016.

55 Abraham C, Michie S. A Taxonomy of Behavior Change Techniques Used in Interventions, *Health Psychol* 2008;27:379-87.

56 Oxford Dictionaries: http://www.oxforddictionaries.com/. Date accessed: June 2016.

57 UCL Centre for Behaviour Change. The Behaviour Change Wheel: http://www.behaviourchangewheel.com/. Date accessed: June 2016.

58 Hoddinott P, Craig LCA, Britten J, et al. A serial qualitative interview study of infant feeding experiences: Idealism meets realism, *BMJ Open* 2012;2.

For peer review only

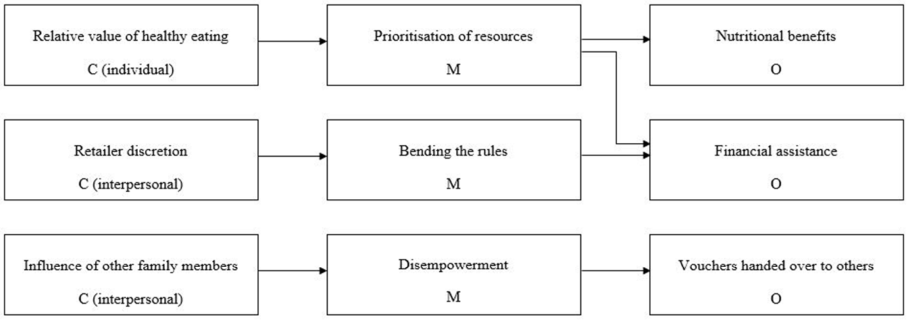

Summary of programme theories about how low-income pregnant women use Healthy Start vouchers

275x107mm (96 x 96 DPI)

For peer review only - http://bmjopen.bmj.com/site/about/guidelines.xhtml

Supplementary file 1. Characteristics of studies included in review (n=38)

Full citation	Intervention (country)	Study design and data collection methods	Contribution to data extraction (C/M/O) and synthesis (programme theories 1-3)					
			C	M	O	1	2	3
Department of Health. Healthy Start: Retailer research summary. London, 2012.	Healthy Start (UK)	Methods unclear	✓	✓			✓	
Hills D, Child C, Junge K, Wilkinson E, Sullivan F. Healthy Start: Rapid evaluation of early impact on beneficiaries, health professionals, retailers and contractors. London: Symbia/Tavistock Institute, 2006.	Healthy Start (UK)	Rapid evaluation; quantitative surveys and qualitative feedback		✓	✓		✓	
Lucas PJ, Jessiman T, Cameron A, et al. Healthy Start Vouchers Study: The Views and Experiences of Parents, Professionals and Small Retailers in England. Bristol: University of Bristol, 2013.	Healthy Start (UK)	Qualitative study; in-depth interviews	✓	✓	✓	✓	✓	✓
McFadden A, Fox-Rushby J, Green JM, et al. Healthy Start: Understanding the use of vouchers and vitamins. Dundee: University of Dundee, 2013.	Healthy Start (UK)	Focus groups; online consultations; workshops and telephone interviews	✓	✓	✓	✓		
Andreyeva T, Middleton AE, Long MW, et al. Food retailer practices, attitudes and beliefs about the supply of healthy foods, *Public Health Nutr* 2011;14:1024-31.	WIC (US)	Before/after study; structured interviews to assess perceived customer demand for healthy foods (mostly quantitative)		✓	✓		✓	
Andreyeva T, Luedicke J, Henderson KE, et al. Grocery store beverage choices by participants in federal food assistance and nutrition programs, *Am J Prev Med* 2012;43:411-8.	WIC (US)	Cross-sectional study; electronic sales data from WIC retailers			✓			
Andreyeva T, Luedicke J, Tripp AS, et al. Effects of reduced juice allowances in food packages for the women, infants, and children program, *Pediatrics* 2013;131:919-27.	WIC (US)	Before/after study; electronic sales data from WIC retailers			✓			

For peer review only

Full citation	Intervention (country)	Study design and data collection methods	Contribution to data extraction (C/M/O) and synthesis (programme theories 1-3)					
			C	M	O	1	2	3
Andreyeva T, Luedicke J. Federal food package revisions: effects on purchases of whole-grain products, *Am J Prev Med* 2013;45:422-9.	WIC (US)	Before/after study; electronic sales data from WIC retailers			✓			
Andreyeva T, Luedicke J, Henderson KE, et al. The positive effects of the revised milk and cheese allowances in the special supplemental nutrition program for women, infants, and children, *Journal of the Academy of Nutrition & Dietetics* 2014;114:622-30.	WIC (US)	Before/after study; electronic sales data from WIC retailers			✓			
Andreyeva T, Luedicke J. Incentivizing fruit and vegetable purchases among participants in the Special Supplemental Nutrition Program for Women, Infants, and Children, *Public Health Nutr* 2015;18:33-41.	WIC (US)	Before/after study; electronic sales data from WIC retailers			✓			
Ayala GX, Laska MN, Zenk SN, et al. Stocking characteristics and perceived increases in sales among small food store managers/owners associated with the introduction of new food products approved by the Special Supplemental Nutrition Program for Women, Infants, and Children, *Public Health Nutr* 2012;15:1771-9.	WIC (US)	Cross-sectional study; in-depth interviews to assess perceived changes in sales of WIC foods (quantitative data from closed questions)			✓			
Bailey LB, O'Farrell-Ray B, Mahan CS, et al. Vitamin B6, iron and folacin status of pregnant women, *Nutr Res* 1983;3:783-93.	WIC (US)	Cross-sectional study; dietary assessment using 24 hour recalls; blood tests for nutrient status			✓			
Bertmann FM, Barroso C, Ohri-Vachaspati P, et al. Women, infants, and children cash value voucher (CVV) use in Arizona: a qualitative exploration of barriers and strategies related to fruit and vegetable purchases, *Journal of Nutrition Education & Behavior* 2014;46:S53-8.	WIC (US)	Qualitative study; focus groups to explore attitudes and behaviours related to FV voucher use	✓		✓			

For peer review only

**Full citation**	**Intervention (country)**	**Study design and data collection methods**	**Contribution to data extraction (C/M/O) and synthesis (programme theories 1-3)**					
			**C**	**M**	**O**	**1**	**2**	**3**
Black MM, Hurley KM, Oberlander SE, et al. Participants' comments on changes in the revised special supplemental nutrition program for women, infants, and children food packages: the Maryland food preference study, *J Am Diet Assoc* 2009;109:116-23.	WIC (US)	Cross-sectional study; questionnaire to assess current and anticipated consumption; focus groups to explore reactions to proposed changes to WIC package			✓			
Chen DY, Gazmararian JA. Impact of personal preference and motivation on fruit and vegetable consumption of WIC-participating mothers and children in Atlanta, GA, *Journal of Nutrition Education & Behavior* 2014;46:62-7.	WIC (US)	Cross-sectional study; questionnaire to assess FV consumption			✓			
Endres J, Dunning S, Poon SW, et al. Older pregnant women and adolescents: nutrition data after enrollment in WIC, *J Am Diet Assoc* 1987;87:1011.	WIC (US)	Cross-sectional study; dietary assessment using 24 hour recalls			✓			
Endres JM, Sawicki M, Casper JA. Dietary assessment of pregnant women in a supplemental food program. *J Am Diet Assoc* 1981;79:121-6.	WIC (US)	Cross-sectional study; dietary assessment using 24 hour recalls			✓			
Ettienne-Gittens R, McKyer ELJ, Odum M, et al. Rural versus Urban Texas WIC participants' fruit and vegetable consumption, *Am J Health Behav* 2013;37:130-40.	WIC (US)	Cross-sectional study; questionnaire to assess FV consumption and variety			✓			
Farrior ES, Ruwe CH. Women, infants and children program. Prenatal participation and dietary intakes, *Nutr Res* 1987;7:451-549.	WIC (US)	Before/after study; dietary assessment using 24 hour recalls			✓			
Gittelsohn J, Laska MN, Andreyeva T, et al. Small retailer perspectives of the 2009 women, infants and children program food package changes, *Am J Health Behav* 2012;36:655-65.	WIC (US)	Qualitative study; in-depth interviews with small store owners and managers	✓	✓	✓		✓	

For peer review only

Full citation	Intervention (country)	Study design and data collection methods	Contribution to data extraction (C/M/O) and synthesis (programme theories 1-3)					
			C	M	O	1	2	3
Herman DR, Harrison GG, Afifi AA, et al. The Effect of the WIC Program on Food Security Status of Pregnant, First-Time Participants, *Family Economics & Nutrition Review* 2004;16:21.	WIC (US)	Longitudinal study; US Food Security Survey Module; qualitative interviews on use of WIC food package as income transfer (presented as quantitative data)			✓	✓		
Herman DR, Harrison GG, Jenks E. Choices made by low-income women provided with an economic supplement for fresh fruit and vegetable purchase, *J Am Diet Assoc* 2006;106:740-4.	WIC (US)	Cross-sectional study; data from retailers on FV purchases			✓			
Herman DR, Harrison GG, Afifi AA, et al. Effect of a targeted subsidy on intake of fruits and vegetables among low-income women in the Special Supplemental Nutrition Program for Women, Infants, and Children, *Am J Public Health* 2008;98:98-105.	WIC (US)	Longitudinal study; dietary assessment using 24 hour recalls			✓			
Horswill LJ, Yap C. Consumption of foods from the WIC food packages of Chinese prenatal patients on the US west coast, *J Am Diet Assoc* 1999;99:1549-53.	WIC (US)	Cross-sectional study; dietary assessment using FFQ	✓		✓			
Kong A, Odoms-Young A, Schiffer LA, et al. Racial/ethnic differences in dietary intake among WIC families prior to food package revisions, *Journal of Nutrition Education & Behavior* 2013;45:39-46.	WIC (US)	Cross-sectional study; dietary assessment using 24 hour recalls	✓		✓			
Kong A, Odoms-Young A, Schiffer LA, et al. The 18-month impact of special supplemental nutrition program for women, infants, and children food package revisions on diets of recipient families, *Am J Prev Med* 2014;46:543-51.	WIC (US)	Longitudinal study; dietary assessment using 24 hour recalls (baseline data from Kong et al. 2013)	✓		✓			

For peer review only

Full citation	Intervention (country)	Study design and data collection methods	Contribution to data extraction (C/M/O) and synthesis (programme theories 1-3)					
			C	M	O	1	2	3
Meiqari L, Torre L, Gazmararian JA. Exploring the Impact of the New WIC Food Package on Low-Fat Milk Consumption Among WIC Recipients: A Pilot Study, *Journal of Health Care for the Poor & Underserved* 2015;26:712-25.	WIC (US)	Before/after study; questionnaire to assess primary consumption of low-fat milk (yes/no)			✓			
Nestor B, McKenzie J, Hasan N, et al. Client satisfaction with the nutrition education component of the California WIC program, *J Nutr Educ* 2001;33:83-94.	WIC (US)	Cross-sectional study; client satisfaction survey; focus groups to assess client experiences and satisfaction	✓	✓	✓			
Odoms-Young A, Kong A, Schiffer LA, et al. Evaluating the initial impact of the revised Special Supplemental Nutrition Program for Women, Infants, and Children (WIC) food packages on dietary intake and home food availability in African-American and Hispanic families, *Public Health Nutr* 2014;17:83-93.	WIC (US)	Longitudinal study; dietary assessment using 24 hour recalls (baseline data from Kong et al. 2013) and questionnaire to assess home food availability			✓			
Pehrsson PR, Moser-Veillon PB, Sims LS, et al. Postpartum iron status in nonlactating participants and nonparticipants in the Special Supplemental Nutrition Program for Women, Infants, and Children, *Am J Clin Nutr* 2001;73:86-92.	WIC (US)	Longitudinal study; iron status using FFQ and blood tests			✓			
Reyes NR, Klotz AA, Herring SJ. A qualitative study of motivators and barriers to healthy eating in pregnancy for low-income, overweight, African-American mothers, *Journal of the Academy of Nutrition & Dietetics* 2013;113:1175-81.	WIC (US)	Qualitative study; semi-structured interviews to explore motivators and barriers to healthy eating	✓	✓	✓			✓

For peer review only

Full citation	Intervention (country)	Study design and data collection methods	Contribution to data extraction (C/M/O) and synthesis (programme theories 1-3)					
			C	M	O	1	2	3
Rush D, Sloan NL, Leighton J, et al. The National WIC Evaluation: evaluation of the Special Supplemental Food Program for Women, Infants, and Children. V. Longitudinal study of pregnant women, *Am J Clin Nutr* 1988;48:439-83.	WIC (US)	Before/after study; food expenditure assessed by recall (and diary)			✓			
Rush D, Kurzon MR, Seaver WB, et al. The National WIC Evaluation: evaluation of the Special Supplemental Food Program for Women, Infants, and Children. VII. Study of food expenditures, *Am J Clin Nutr* 1988;48:512-9.	WIC (US)	Longitudinal study; dietary assessment using 24 hour recalls			✓			
Swensen AR, Harnack LJ, Ross JA. Nutritional assessment of pregnant women enrolled in the Special Supplemental Program for Women, Infants, and Children (WIC), *J Am Diet Assoc* 2001;101:903-8.	WIC (US)	Cross-sectional study; dietary assessment using FFQ; blood tests for iron status			✓			
Treiman K, Freimuth V, Damron D, et al. Attitudes and behaviors related to fruits and vegetables among low-income women in the WIC program, *J Nutr Educ Behav* 1996;28:149-56.	WIC (US)	Qualitative study; focus groups to explore attitudes and behaviours related to FV; central location intercept interviews (questionnaires)	✓	✓	✓	✓		✓
Watts V, Rockett H, Baer H, et al. Assessing diet quality in a population of low-income pregnant women: a comparison between Native Americans and whites, *Maternal & Child Health Journal* 2007;11:127-36.	WIC (US)	Cross-sectional study; FFQ to assess diet quality			✓			
Whaley SE, Ritchie LD, Spector P, et al. Revised WIC food package improves diets of WIC families, *Journal of Nutrition Education & Behavior* 2012;44:204-9.	WIC (US)	Cross-sectional study; questionnaire to assess usual dietary habits and changes compared to 6 months earlier			✓			

For peer review only

Full citation	Intervention (country)	Study design and data collection methods	Contribution to data extraction (C/M/O) and synthesis (programme theories 1-3)					
			C	M	O	1	2	3
Wunderlich SM, Hongu NK, Courter A, et al. Nutrient intake and nutritional status of low-income pregnant women, *Topics in Clinical Nutrition* 1996;12:66-74.	WIC (US)	Cross-sectional study; dietary assessment using 24 hour recalls			✓			

WIC = Special Supplemental Program for Women, Infants, and Children; FV = fruits and vegetables; Programme theories: 1. Prioritisation of resources; 2. Bending the rules; 3. Disempowerment

For peer review only

List of items to be included when reporting a realist synthesis

**TITLE**			**Page**
1		In the title, identify the document as a realist synthesis or review	1
ABSTRACT			
2		While acknowledging publication requirements and house style, abstracts should ideally contain brief details of: the study's background, review question or objectives; search strategy; methods of selection, appraisal, analysis and synthesis of sources; main results; and implications for practice.	2
INTRODUCTION			
3	Rationale for review	Explain why the review is needed and what it is likely to contribute to existing understanding of the topic area.	4
4	Objectives and focus of review	State the objective(s) of the review and/or the review question(s). Define and provide a rationale for the focus of the review.	4-5
METHODS			
5	Changes in the review process	Any changes made to the review process that was initially planned should be briefly described and justified.	NA
6	Rationale for using realist synthesis	Explain why realist synthesis was considered the most appropriate method to use.	4
7	Scoping the literature	Describe and justify the initial process of exploratory scoping of the literature.	5-6
8	Searching processes	While considering specific requirements of the journal or other publication outlet, state and provide a rationale for how the iterative searching was done. Provide details on all the sources accessed for information in the review. Where searching in electronic databases has taken place, the details should include, for example, name of database, search terms, dates of coverage and date last searched. If individuals familiar with the relevant literature and/or topic area were contacted, indicate how they were identified and selected.	6
9	Selection and appraisal of documents	Explain how judgements were made about including and excluding data from documents, and justify these.	7-8
10	Data extraction	Describe and explain which data or information were extracted from the included documents and justify this selection.	8
11	Analysis and synthesis processes	Describe the analysis and synthesis processes in detail. This section should include information on the constructs analyzed and describe the analytic process.	9
RESULTS			
12	Document flow diagram	Provide details on the number of documents assessed for eligibility and included in the review with reasons for exclusion at each stage as well as an indication of their source of origin (for example, from searching databases, reference lists and so on).	9 and 21

For peer review only

TITLE			Page
		You may consider using the example templates (which are likely to need modification to suit the data) that are provided.	
13	Document characteristics	Provide information on the characteristics of the documents included in the review.	9-11
14	Main findings	Present the key findings with a specific focus on theory building and testing.	11-13
DISCUSSION			
15	Summary of findings	Summarize the main findings, taking into account the review's objective(s), research question(s), focus and intended audience(s).	13
16	Strengths, limitations and future research directions	Discuss both the strengths of the review and its limitations. These should include (but need not be restricted to) (a) consideration of all the steps in the review process and (b) comment on the overall strength of evidence supporting the explanatory insights which emerged.<br><br>The limitations identified may point to areas where further work is needed.	13-14
17	Comparison with existing literature	Where applicable, compare and contrast the review's findings with the existing literature (for example, other reviews) on the same topic.	14-15
18	Conclusion and recommendations	List the main implications of the findings and place these in the context of other relevant literature. If appropriate, offer recommendations for policy and practice.	15
19	Funding	Provide details of funding source (if any) for the review, the role played by the funder (if any) and any conflicts of interests of the reviewers.	16

Source of this checklist:

http://www.equator-network.org/reporting-guidelines/rameses-publication-standards-realist-syntheses/

http://www.ramesesproject.org/Standards_and_Training_materials.php

http://bmcmedicine.biomedcentral.com/articles/10.1186/1741-7015-11-21#Tab1

Page numbers refer to pdf document created during submission process.

For peer review only

PRISMA 2009 Flow Diagram

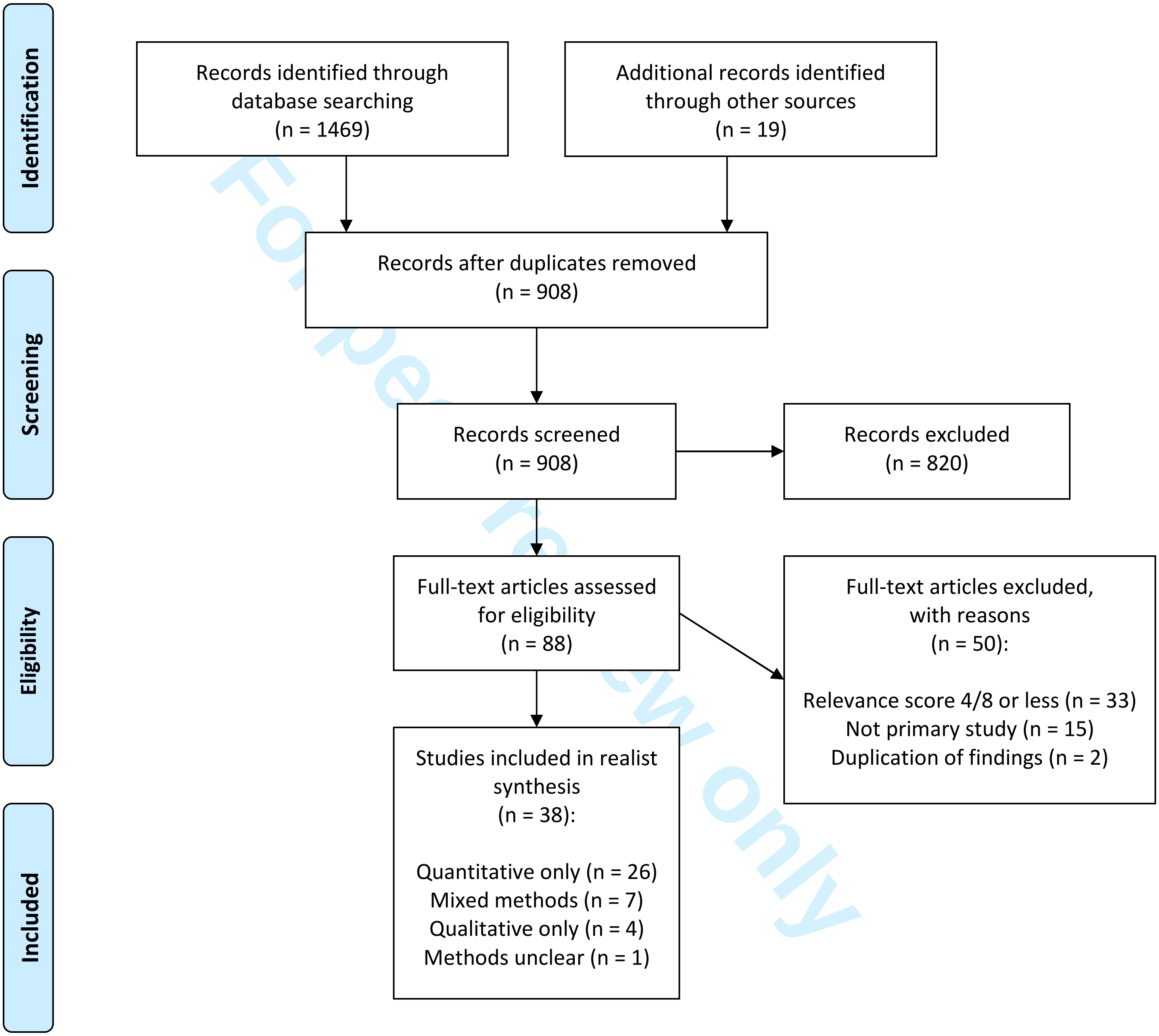

From: Moher D, Liberati A, Tetzlaff J, Altman DG, The PRISMA Group (2009). *P*referred *R*eporting *I*tems for *S*ystematic Reviews and *M*eta-*A*nalyses: The PRISMA Statement. PLoS Med 6(6): e1000097. doi:10.1371/journal.pmed1000097

For more information, visit www.prisma-statement.org.

For peer review only

For peer review only - http://bmjopen.bmj.com/site/about/guidelines.xhtml

A realist review to explore how low-income pregnant women use food vouchers from the UK's Healthy Start programme

Journal:	*BMJ Open*
Manuscript ID	bmjopen-2016-013731.R2
Article Type:	Research
Date Submitted by the Author:	22-Nov-2016
Complete List of Authors:	Ohly, Heather; University of Central Lancashire, College of Health and Wellbeing<br>Crossland, Nicola; University of Central Lancashire, College of Health and Wellbeing<br>Lowe, Nicola; University of Central Lancashire, College of Health and Wellbeing<br>Dykes, Fiona; University of Central Lancashire, College of Health and Wellbeing<br>Hall-Moran, Victoria; University of Central Lancashire, College of Health and Wellbeing
**Primary Subject Heading**:	Public health
Secondary Subject Heading:	Nutrition and metabolism
Keywords:	pregnant, women, food, vouchers, realist, review

For peer review only - http://bmjopen.bmj.com/site/about/guidelines.xhtml

Title of the article

A realist review to explore how low-income pregnant women use food vouchers from the UK's Healthy Start programme

Full name, postal address, email and telephone number of corresponding author:

Heather Ohly, Brook Building 215, College of Health and Wellbeing, University of Central Lancashire, Preston, UK

HOhly@uclan.ac.uk

07866 318014

Full name, department, institution, city and country of all co-authors:

Heather Ohly, College of Health and Wellbeing, University of Central Lancashire, Preston, UK

Nicola Crossland, College of Health and Wellbeing, University of Central Lancashire, Preston, UK

Nicola Lowe, College of Health and Wellbeing, University of Central Lancashire, Preston, UK

Fiona Dykes, College of Health and Wellbeing, University of Central Lancashire, Preston, UK

Victoria Hall Moran, College of Health and Wellbeing, University of Central Lancashire, Preston, UK

Word count, excluding title page, abstract, references, figures and tables:

5041 words

For peer review only

ABSTRACT

Objectives: To explore how low-income pregnant women use Healthy Start food vouchers, the potential impacts of the programme, which women might experience these impacts and why.

Design: A realist review.

Eligibility criteria for selecting studies: Primary or empirical studies (of any design) were included if they contributed relevant evidence or insights about how low-income women use food vouchers from the Healthy Start (UK) or the Special Supplemental Nutrition Program for Women, Infants and Children (US) programmes. The assessment of 'relevance' was deliberately broad to ensure that reviewers remained open to new ideas from a variety of sources of evidence.

Analysis: A combination of evidence synthesis and realist analysis techniques was used to modify, refine and substantiate programme theories, which were constructed as explanatory 'context – mechanism – outcome' (CMO) configurations.

Results: 38 primary studies were included in this review: four studies on Healthy Start and 34 studies on WIC. Two main outcome strands were identified: dietary improvements (intended) and financial assistance (unintended). Three evidence-informed programme theories were proposed to explain how aspects of context (and mechanisms) may generate these outcomes: the 'relative value' of healthy eating (prioritisation of resources); retailer discretion (pressure to 'bend the rules'); the influence of other family members (disempowerment).

Conclusions: This realist review suggests that some low-income pregnant women may use Healthy Start vouchers to increase their consumption of fruits and vegetables and plain cow's milk, whereas others may use them to reduce food expenditure and save money for other things.

Key words: pregnant, women, food, vouchers, realist, review

For peer review only

Strengths and limitations of this study:

- First study to articulate, develop and test programme theories about Healthy Start
- Inclusion of relevant studies from a similar programme in the US (WIC)
- Some evidence not transferable due to population and programme differences
- Insufficient evidence to link CMO configurations to sociodemographic and cultural characteristics

For peer review only

BACKGROUND

Healthy Start is the United Kingdom (UK) government's food voucher programme for low-income pregnant women and young children. It was introduced in 2006, after the Acheson Review drew attention to income as one of the major determinants of health (and nutrition) inequalities, and highlighted the importance of nutrition for women of childbearing age and their children.[1] The Committee on Medical Aspects of Food and Nutrition Policy (COMA) was asked by the government to review the longstanding Welfare Food Scheme, which was subsequently replaced by Healthy Start.[2]

Women are eligible for Healthy Start if they receive income-related benefits or child tax credit and an annual household income of £16,190 or less. Pregnant women aged under 18 are eligible regardless of their income. The weekly voucher values are: one voucher per week during pregnancy (£3.10); two vouchers per week for each baby under one year (£6.20) and one voucher per week for each child aged 1-4 years (£3.10). The vouchers can be exchanged for fruits and vegetables, plain cow's milk or infant formula. Retailers must be registered with the scheme to accept and claim payment for the vouchers. Healthy Start also offers free vitamins for eligible women and children, but there have been problems with uptake of vitamins.[3] Some areas offer free vitamins to all pregnant women and young children and the option of universalising Healthy Start vitamins remains under review (at the time of writing) by the Chief Medical Officer. Therefore, this review focused on the food voucher component of the programme and low-income pregnant women as the first beneficiaries.

Healthy Start aims to provide a "basic nutritional safety net" and to encourage "women and families to make positive nutritional choices affecting their longer term health" (page 4).[4] Therein lies an implicit programme assumption that women will use the vouchers to purchase target foods (ideally in greater quantities than they did before) leading to dietary improvements. The vouchers may have been conceived as a financial incentive for dietary improvements, although this was not explicit in policy documentation. Since the introduction of Healthy Start in 2006, there has been no robust evaluation of its impact on nutritional outcomes – despite recommendations published in an early scoping review[5] However, qualitative studies have indicated a range of experiences, motivations and perceived outcomes, with not all low-income women using the vouchers to improve their diets.[6, 7] This review was undertaken to explore how low-income pregnant women use Healthy Start

For peer review only

food vouchers, the potential impacts of the programme, which women might experience these impacts and why.

The realist approach was adopted because it is based on the understanding that different individuals or groups of individuals are likely to respond to any programme (or intervention) in different ways. It is a theory-driven approach that seeks to explore outcome patterns (or demi-regularities) and offer plausible explanations for how and why they occur. The purpose of realist synthesis is to "articulate underlying programme theories and then to interrogate the existing evidence to find out whether and where these theories are pertinent and productive" (page 74).[8] The stages of conducting a realist review tend to be iterative and overlapping – a gradual process of developing, testing and refining programme theories. Evidence may be obtained from studies of the programme itself, or more widely from similar programmes that are thought to work in similar ways. Reviewers may adapt and modify existing theoretical frameworks or 'middle-range' theories to help develop their own explanations. The unit of analysis is the programme theory (rather than the specific programme) and this can be considered at different levels of abstraction.

Programme theories are often constructed as 'context – mechanism – outcome' (CMO) configurations, and evidence is sought to substantiate the causal linkages. The logic of realist explanation is that outcomes (O) are caused by mechanisms (M), and mechanisms may (or may not) be 'triggered' in certain contexts (C).[8] Context refers to the pre-existing conditions into which the programme is introduced, and there are four layers of context: individual, interpersonal, institutional and infrastructural. Mechanisms are defined as the reasoning and reactions of individuals in response to the resources offered by the programme. A core principle of realism is that mechanisms generate outcomes – they are not a direct result of the programme.

This realist review aimed to explore the following questions:

1. How do low-income pregnant women use Healthy Start vouchers?
2. What are the intended and unintended outcomes of the programme?
3. What are the underlying mechanisms and how do variations in context influence (enable or constrain) these mechanisms?

For peer review only

A preliminary search confirmed the paucity of empirical studies on Healthy Start and we felt that additional sources of empirical evidence would be needed to explore these research questions. The most obvious source of potentially relevant evidence was the Special Supplemental Nutrition Program for Women, Infants and Children (WIC) in the United States (US). WIC is the only other national food subsidy programme for low-income women of childbearing age, including pregnant and postpartum women, and young children. It was first introduced in 1972 and revised in 2009 to reflect current dietary guidelines.[9] The WIC food package for pregnant women is different to Healthy Start, with 'maximum monthly allowances' for a range of foods (reduced-fat milk, whole grain cereals, eggs and pulses) and 'cash value vouchers' for fruits and vegetables. The other main programme difference is mandatory WIC nutrition education for all beneficiaries. There are also many contextual differences between the US and the UK, such as socio-demographic, cultural, geographical and political characteristics. Despite these differences, there are also likely to be similarities in terms of how low-income women respond to the programme (i.e. mechanisms).

METHODS

Study protocol and ethical approval

The protocol for this realist review was registered with the International Prospective Register of Systematic Reviews (PROSPERO 2014: CRD42014015050). There were no changes to the review process since this protocol was published. Ethical approval was obtained from the University of Central Lancashire Science, Technology, Engineering, Medicine and Health (STEMH) Ethics Committee in May 2015.

Programme theory development

This review is about a programme that already exists, with implicit theories and assumptions about how it works and what effects it may have on beneficiaries.[8] Therefore, we used a 'bottom up' approach to theory development in this review, as described by Shearn and Allmark (Realist Research Seminar Series, Sheffield Hallam University, 2016). In other words, we developed theories using information about the Healthy Start food voucher programme, rather than theorising at a more abstract level. Candidate theories (or initial, untested theories) were identified and prioritised using information derived from academic and grey literature on Healthy Start, an intervention mapping exercise, existing knowledge, creative thinking, consultations with stakeholders (in person and by email) and discussions

For peer review only

among the review team. The stakeholder group included six midwives, two academics and two public health practitioners, who all shared first hand experiences and insights about how the Healthy Start programme works in practice.

We developed candidate theories about how low-income pregnant women might experience Healthy Start, and what contextual factors might influence their reasoning and reactions (mechanisms) at each of the following stages: access to the programme starting at the first trimester antenatal appointment (e.g. issues around clear communication and understanding of eligibility criteria and entitlement); the application process (e.g. barriers and facilitators to successfully receiving the vouchers); whether and where women use the food vouchers (e.g. issues around convenience and stigmatisation); and how women use the food vouchers (e.g. to buy more of the target foods or to save money). We decided to prioritise the last stage of the programme pathway: once an eligible pregnant woman has received the food vouchers, how does she use them and why? This decision reflected the research priorities identified from the literature, the interests of the research team, and the time and resources available to conduct this review. The theories we tested were proposed explanations for why some pregnant women might use Healthy Start vouchers to improve their diets, and other women might use them to save money.

Search strategy

Separate searches were conducted for Healthy Start and WIC:

Healthy Start – Studies were identified through manual, purposive, snowball and citation searches (January to March 2015). The search terms used initially were ‘Healthy Start’ and ‘UK’ because there is another programme called Healthy Start in the US, which aims to prevent infant mortality. More precise search terms were not needed due to the paucity of empirical studies and familiarity with the literature.

WIC – A broad search strategy was devised in collaboration with an Information Specialist in the Collaboration for Leadership in Applied Health Research and Care (CLAHRC) North West Coast. This strategy was adapted and run in six electronic databases in September 2015: MEDLINE, EMBASE, CINAHL, Open Grey, ETHOS and PubMed. Table 1 shows the search terms used in MEDLINE. No date or language restrictions were used. Reference lists of included studies were checked for additional studies. An online list of WIC studies was also checked for additional studies.[10]

For peer review only

For peer review only

Table 1. Search strategy used in MEDLINE to identify WIC studies

#	Search terms	Results
1	WIC.tw.	1008
2	(nutrition or food or voucher or "nutrition program").tw.	377002
3	1 and 2	599
4	(Special* adj4 Supplement* adj4 Nutrit* adj4 Program* adj4 Women* adj4 Infant* adj4 Child*).tw.	415
5	3 or 4	688

Inclusion criteria

Primary or empirical studies (of any design) were included if they contributed relevant evidence or insights about how low-income women use food vouchers from the Healthy Start (UK) or WIC (US) programmes. An assessment of 'relevance' is essential in realist synthesis, to ensure that all included studies contribute to theory development, refinement and testing.[8, 11] In this review, the interpretation of 'relevance' was deliberately broad to ensure that reviewers remained open to new ideas from a variety of sources of evidence. A bespoke system was used to maintain a consistent and transparent approach. Table 2 shows the questions used to assess relevance. These questions were developed by the review team and finalised towards the end of the theory development stage, to ensure they reflected the candidate theories we wanted to test. Studies that scored 5/8 or more (based on the total number of yes answers) were included.

For peer review only

Table 2. Questions used to assess the relevance of primary studies

#	Question:
1	Do the research questions or study aims refer to Healthy Start or WIC?
2	Does the study focus on the food voucher (CVV or food package for WIC) component of the programme?
3	Does the study focus on beneficiaries (women who were receiving the vouchers) rather than eligibility status (women who were eligible to receive the vouchers)?
4	Does the sample include pregnant women?
5	If the sample does not include pregnant women, could some of the findings be generalizable to pregnant women?
6	Does the study report women's food or nutrient intakes (measured or perceived)?
7	Does the study provide any insights about how food vouchers are used?
8	Does the study provide any insights about which women may benefit most/least and why?

Study selection

Results from the WIC database searches were uploaded into RefWorks (web version; ProQuest; Michigan, US) and screened using titles and abstracts. Studies that appeared to meet the inclusion criteria were obtained as full text articles. Studies for which insufficient information was available to determine relevance were also obtained as full text articles. The full text screening process was fully documented using Microsoft Excel 2013 (version 15.0.4815.1001; Microsoft Corporation), including assessments of relevance and reasons for exclusions. The same criteria were applied to studies of Healthy Start. Study selection was completed by the lead reviewer (HO) and double checked by a second reviewer (VM). Any disagreements were resolved by discussion.

Data extraction

Quantitative data on women’s nutritional outcomes were extracted using bespoke tables in Microsoft Word 2013 (version 15.0.4815.1001; Microsoft Corporation). Other non-relevant data were not extracted. Qualitative data, textual descriptions of findings, and author interpretations were extracted using MAXQDA 11 (version 11; VERBI Software GmbH; Berlin). A coding system was created with three main headings: context, mechanisms and outcomes. Subheadings were added deductively (based on candidate theories) and inductively

For peer review only

(as new themes emerged from the data). Data extraction was completed by the lead reviewer (HO) and a sample was double checked by a second reviewer (NC).

Quality appraisal

Studies were not formally appraised at the data extraction stage, as would be the case in traditional systematic reviews. Instead, an assessment of 'rigour' was used to judge the credibility and trustworthiness of the evidence as it was integrated into the analysis and synthesis.[8, 11] This assessment was not scored because weaker studies were still included, but it meant that methodological limitations were acknowledged and study findings were not over-interpreted. Table 3 shows the questions used to assess rigour. Quality appraisal was completed by the lead reviewer (HO) and double checked by second reviewers (NL/VM).

Table 3. Questions used to assess the rigour of primary studies

#	Question:
1	Are the study methods clearly reported (including study design, recruitment, data collection and analysis)?
2	Are the study methods appropriate to answer the research questions?
3	Are the sample characteristics reported to enable judgements about generalisability?
4	Are the study findings and conclusions supported by raw data?
5	Are the study limitations acknowledged and clearly reported?

Analysis and synthesis

This process involved gradual and iterative theory development, whereby evidence from primary studies was used to modify, refine and substantiate programme theories about how low-income pregnant women use Healthy Start vouchers, in what circumstances, and why. Theories were constructed as explanatory 'context – mechanism – outcome' (CMO) configurations, usually by starting with the outcome and working backwards to determine "what caused it (the mechanism) and under what contexts was the mechanism triggered".[12] The main focus of the analysis was searching for evidence to support and refute the proposed causal linkages between context, mechanisms and outcomes. A combination of evidence synthesis and realist analysis techniques was used:

For peer review only

1. Narrative synthesis of quantitative data on women's nutritional outcomes; meta-analysis was not appropriate due to heterogeneity of study designs and data collection methods (and was beyond the scope of this review).
2. Thematic synthesis of qualitative data, by creating codes and themes (as described under data extraction) and then 'going beyond' the interpretations of the original studies to generate new understandings or hypotheses.[13]
3. Creative theorising or 'retroduction' by the lead reviewer (HO) in collaboration with the review team and the stakeholder group. This involved in-depth reflection and discussions (throughout the review) about the underlying causes of outcome patterns, at the level of generative mechanisms and explanatory context. The data from included studies did not always provide such in-depth insights and explanations. Where individual extracts of data only supported part of the CMO configuration, it was necessary to make logical inferences about the complete causal pathways and explanations.[14]

RESULTS

Search results and study characteristics

A total of 908 records were identified through the two separate searches. After screening titles and abstracts, 88 records were obtained in full text format. Fifty full text articles were excluded based on the assessment of relevance (n=33) or because they were not primary studies (n=15) or the findings were duplicated (n=2). Therefore, 38 primary studies were included in this review: four UK studies on Healthy Start[6, 7, 15, 16] and 34 US studies on WIC[17-50] (see PRISMA Flow Diagram and online supplementary file 1).

Identification of outcomes and supporting evidence

Two main outcome strands emerged during the theory development stage and were further substantiated using evidence from primary studies:

1. Women use vouchers to increase consumption of target foods – dietary improvements.
2. Women use vouchers to reduce food expenditure – financial assistance.

For the purposes of this review, we have assumed that strand 1 is the intended outcome of the programme and strand 2 is an unintended outcome. This was not explicit in policy documentation, but there were references to dietary improvements, which were thought to be

For peer review only

achieved by enabling low-income women to access healthier foods and encouraging positive nutritional choices.[4] The included studies provided support for both outcome strands. The next section provides an overview of the available evidence on women's outcomes (intended and unintended) and highlights the relative contribution of evidence from Healthy Start and WIC studies. It also helps to illustrate how we worked backwards from outcomes to identify generative mechanisms and related aspects of context.

A total of 25 studies reported women's nutritional outcomes: three studies on Healthy Start and 22 studies on WIC. The Healthy Start studies reported perceived outcomes only; some women said they consumed more cow's milk, fruits and vegetables after receiving Healthy Start vouchers,[6, 7, 16] whereas other women said the vouchers "freed up money to do other things" and "helped them to manage better financially" (page 59).[7] The WIC studies were published between 1981 and 2015, but the most useful data was extracted from two studies comparing women's diets before and after the 2009 WIC revisions, when the 'cash value vouchers' for fruits and vegetables were introduced (there was no allowance for fresh fruits and vegetables before 2009). A longitudinal study of African American and Hispanic women from WIC clinics in Chicago (n=273) found significant dietary improvements for Hispanic mothers, who reported consuming more fruit, more reduced fat milk less whole milk and less saturated fat (all $p<0.05$).[39] African American mothers reported consuming less whole milk ($p=0.02$) but no other changes were statistically significant.[41] There were no sustained dietary improvements in either group compared to baseline at 18 months.[38] A cross-sectional study comparing two random samples of WIC participants in California (both 80% Hispanic) found that women assessed six months after the changes (n=2996) reported consuming significantly more whole grains, reduced-fat milk and vegetables, and less whole milk, compared to women assessed before the changes (n=3004) (all $p<0.001$).[49]

Five studies reported electronic sales data from WIC retailers (one large supermarket chain) in New England, which showed that women's purchasing patterns shifted towards items provided in the WIC package after the 2009 revisions – fruits and vegetables, reduced-fat milk, brown rice, whole grain cereals and bread replaced less nutritious options.[18-22] One study (n=2137) showed that, while total spending on fruits and vegetables increased between 2009 and 2010 ($p<0.001$), up to 13% fewer purchases were made using non-WIC funds.[22] This implies that some women 'substituted' the method of payment, rather than using WIC to increase the amount of fruits and vegetables purchased. None of these studies reported

For peer review only

sample characteristics such as ethnicity. Finally, a mixed methods study of Hispanic and African American pregnant women (n=313) found that two thirds of women reported using WIC vouchers to reduce food expenditure.[33] The money they saved was used to purchase items for the unborn baby, other foods, and for bills and emergencies.

These findings suggest that food vouchers may lead to dietary improvements for some, but not all women. This may be because some women use the vouchers to pay for foods they would previously have bought using cash. The WIC studies described above were not representative of ethnic groups in the UK, and the samples included mothers as well as pregnant women. However, these studies provide much needed evidence on the potential impact of food vouchers for low-income women, which was not available from the Healthy Start literature alone.

Evidence-informed programme theories

This section presents three evidence-informed programme theories, which help to explain why different women receive the same Healthy Start vouchers and yet experience different outcomes, due to variations in context and mechanisms. Figure 1 illustrates the key aspects of context, mechanisms and outcomes identified and the proposed causal pathways linking them together. These causal pathways are explained as CMO configurations and illustrated using quotations from included studies under each theory. Although low income is clearly an important aspect of context, we have not included it in our programme theories because it applies to all women who receive Healthy Start vouchers (apart from under 18s). As the programme theories explain, some women may achieve dietary improvements despite low income, and other women may not – this divergence depends on other aspects of context.

1. Prioritisation of resources

Women living on low incomes must constantly prioritise how they spend their money. Food vouchers may be considered as one part of the household resources and decisions must be made about how best to use the vouchers. A key aspect of individual level context is the ‘relative value’ of healthy eating (compared to other things women value), which can lead women to prioritise in different ways. Context is not static and women’s values may change over time, so we propose that some women may fluctuate between the mechanisms (ways of prioritising) outlined in these two contrasting CMO configurations.

For peer review only

Women who value healthy eating and aspire to eat well during pregnancy [context][47] are more likely to perceive Healthy Start vouchers as an opportunity to achieve health benefits for themselves and their unborn baby [mechanism].[6] The vouchers alleviate the financial barrier associated with healthy eating and make healthy foods seem more affordable [mechanism].[6] Therefore, women who value healthy eating are more likely to prioritise healthy eating [mechanism] and use Healthy Start vouchers to increase consumption of target foods – fruits and vegetables or cow's milk [outcome].[6]

> "I have them at Asda when I do my shop, and I think how many vouchers I've got and I buy the veg that I have the vouchers for. I suppose if I didn't have the vouchers, I would just pick out the little things. I don't think if I didn't have the vouchers I'd buy half as much, no." (Mother, UK; page 50)[6]

Alternatively, women may value healthy eating less than other things they want or need to spend money on, which are considered more important or urgent [context].[6, 7] They are more likely to perceive Healthy Start vouchers as a way to save money, which can be redirected and prioritised in other ways [mechanism].[6, 7] These women are more likely to use Healthy Start vouchers to deduct money from the shopping bill, with no increase in consumption of target foods [outcome].[6, 7, 33]

> "Women are often in a dilemma about whether they should or shouldn't eat healthy foods because something else is needed more. Their own health and maybe the health of their younger children are on the back burner because something else is more pressing." (Midwife, UK; page 35)[6]

2. Bending the rules

The Healthy Start voucher exchange system relies on registered retailers to verify (visually) at the checkout that women have selected appropriate amounts of appropriate items – fruits and vegetables, plain cow's milk or infant formula. The vouchers are processed by swiping a barcode that subtracts the voucher value (£3.10) from the total. They are not electronically matched to specific food items. There is a reminder printed on each voucher about which foods may be purchased, along with a warning about prosecution, but the evidence suggests that some customers and retailers appear to disregard this information.

For peer review only

Retailers who are registered to accept Healthy Start vouchers have some discretion over how vigilantly they check what vouchers are spent on [context].[6, 15] Women may put pressure on retailers to ‘bend the rules’ or make exceptions [mechanism].[6, 15-17, 32] Some retailers may decide to ‘turn a blind eye’ because they feel duty bound to help families in whatever ways they can[mechanism][6] or because they prefer to avoid conflict [mechanisms].[16] This enables women to exchange the vouchers for alternative food or non-food items [outcomes].[6, 16]

> “But you have to realise that I get people coming in here, they are buying £1 pound of electricity every day. £1. That must run out after an hour. How do they live? And in the winter, it really does get very cold and they come in and ask me if they can use the voucher for electricity. What can I do? I can’t see them living in the flat with young children, with no heating, it’s so cold. So I do let them do that. They come in and show me their empty wallet and I have to believe them and I do sell gas and electricity for the voucher. You can report that back. I don’t care, what can I do?” (Retailer, UK; page 69)[6]

3. Disempowerment

Pregnant women (and later their young children) are the intended beneficiaries of Healthy Start, but some women may not be empowered to make decisions about how to use the vouchers themselves. The vouchers are posted to women at their home address, but there is no name printed on the actual vouchers and no identification is required at the checkout, so there is nothing to stop other people from using them. Regardless of what is bought with the vouchers, and who benefits, this would surely be considered an unintended outcome of the programme.

Women may not be empowered to make decisions about household resources or food shopping, such as pregnant teenagers who live with their parents [context],[6, 47] or women who live in large, multi-generational households [context].[43] Women who are disempowered are more likely to hand over their Healthy Start vouchers to other family members [mechanism][43] who then decide how they are used [outcome].[43]

For peer review only

"She (mom) makes most of the decisions. We get the same thing every time we go shopping." (African-American mother living in multi-generational household, US; page 5)[43]

DISCUSSION

Statement of principal findings

This realist review identified two main outcome strands for low-income pregnant women using food vouchers from the Healthy Start programme: dietary improvements (intended) and financial assistance (unintended). Three evidence-informed programme theories were developed and refined to explain how aspects of context (and mechanisms) may generate these outcomes: the 'relative value' of healthy eating (prioritisation of resources); retailer discretion (pressure to 'bend the rules'); the influence of other family members (disempowerment).

Strengths of this review

This was the first study to use a realist, theory-driven approach to investigate how low-income pregnant women use Healthy Start vouchers, in what circumstances, and why. The inclusion of relevant studies from a similar programme in the US (WIC) provided insights and explanations beyond what was available from the Healthy Start literature. This was the first time that researchers have attempted to articulate, develop and test programme theories about how Healthy Start works. Our findings suggest that Healthy Start vouchers are not always used to achieve dietary improvements. Some low-income pregnant women may need to receive more support to increase the perceived value of healthy eating. Modifications to the voucher verification system would help to prevent the vouchers being used by other people and exchanged for alternative items. We hope this review will stimulate discussions about future evaluation needs and programme development.

Limitations of this review

We were aware from the outset that some of the evidence from WIC studies would not be transferable to Healthy Start due to population and programme differences. Further evidence from the UK is needed to develop some of the other candidate theories we identified. This review focused on women's outcomes from the programme, and the aspects of context explored in our theories were individual (women's values and perceptions) and interpersonal

For peer review only - http://bmjopen.bmj.com/site/about/guidelines.xhtml

(interactions with retailers and other family members).[8] We did not find sufficient evidence to link our CMO configurations with sociodemographic and cultural characteristics, such as which groups of women are more/less likely to value healthy eating. Future reviews in this area could include wider evidence on voucher programmes to provide insights about how the mechanisms we have identified might operate in different contexts and different programmes. This was beyond the scope of this review due to time and resource limitations. Finally, we did not explore theories relating to Healthy Start vitamins, women's decisions about infant feeding or children's outcomes. These would all be worthy areas for realist investigations.

Comparisons with existing literature and theories

Healthy Start may have been conceived as (or assumed to be) a financial incentive for dietary improvements. Previous systematic reviews have shown that financial incentives (including taxes, subsidies, vouchers and cash payments) are associated with short term dietary improvements.[51-54] However, no studies of Healthy Start met the inclusion criteria for these reviews and the impact of the programme on women's nutritional outcomes remains unclear. There is also some uncertainty around the definition and function of an incentive in relation to behaviour change, and whether Healthy Start fits into this category of interventions. A recent taxonomy of behaviour change techniques defined 'incentives' as rewarding and contingent on behaviour change.[55] Healthy Start does not fit this definition, since the vouchers are provided to all eligible women regardless of how they are subsequently used. A broader definition of incentive is "a thing that motivates or encourages someone to do something".[56] This review found that Healthy Start vouchers may encourage some low-income pregnant women to improve their diets, by providing the resources to buy additional healthy foods at no extra cost. It also highlighted other possible responses to the same resources, which may generate unintended outcomes. A recent paper by the Centre for Diet and Activity Research (CEDAR) considered the role of individual agency in public health interventions, concluding that 'low agency' interventions are more likely to be effective and equitable by reducing the need for individual decisions.[57] Food vouchers for free fruits and vegetables were positioned in the middle of a continuum of the degree of agency required to benefit from the intervention. Agency is synonymous with realist mechanisms (the reasoning and reactions of individuals in response to the resources offered by the programme) and this review illustrates the contribution of realist methodology to understanding the differential impacts of public health interventions or programmes.

For peer review only

CONCLUSION

This realist review suggests that some low-income pregnant women use Healthy Start vouchers to increase their consumption of fruits and vegetables and plain cow's milk (intended outcome: dietary improvements) whereas other women use them to reduce food expenditure and save money for other things (unintended outcome: financial assistance). We have identified some aspects of context (the 'relative value' of healthy eating, retailer discretion and the influence of other family members) and mechanisms (prioritisation of resources, pressure to 'bend the rules' and disempowerment) that are likely to be important in determining these outcomes. Further evidence is needed to understand how low-income pregnant women could be better supported to prioritise healthy eating and use Healthy Start vouchers to improve their diets during pregnancy – in particular to buy more fruits and vegetables.

For peer review only

STATEMENTS

Acknowledgements: The authors would like to thank the stakeholder group for their contributions to theory development, and Eleanor Kotas (Information Specialist) for her assistance with the electronic database searches.

Contributors: HO was the lead reviewer, while other authors completed the second reviewer tasks (as indicated in the Methods). All authors contributed to the development of programme theories through regular discussions. HO drafted the initial manuscript and all authors contributed to the final manuscript.

Funding: This work was supported by a PhD Studentship (2014-2017) from the Collaboration for Leadership in Applied Health Research and Care North West Coast (CLAHRC NWC).

Declaration: The views expressed are those of the authors and not necessarily those of the DWP, NHS, NIHR or the Department of Health.

Competing interests: None declared.

Data sharing statement: No additional data are available.

For peer review only

REFERENCES

1 Acheson D. Independent Inquiry into Inequalities in Health Report. London: The Stationery Office 1998.

2 Committee on Medical Aspects of Food and Nutrition Policy. Scientific review of the Welfare Food Scheme. London, 2002.

3 McFadden A, Green JM, McLeish J, et al. Healthy Start vitamins – a missed opportunity: findings of a multimethod study, *BMJ Open* 2015;5.

4 Department of Health. Healthy Start: Equality impact assessment. London, 2010.

5 Dyson L, Renfrew MJ, Jenkins R, et al. Approaches to evaluating Healthy Start – a scoping review. Public Health Research Consortium, 2007.

6 Lucas PJ, Jessiman T, Cameron A, et al. Healthy Start Vouchers Study: The Views and Experiences of Parents, Professionals and Small Retailers in England. Bristol: University of Bristol, 2013.

7 McFadden A, Fox-Rushby J, Green JM, et al. Healthy Start: Understanding the use of vouchers and vitamins. Dundee: University of Dundee, 2013.

8 Pawson R. Evidence-based policy: a realist perspective. London: Sage 2006:196.

9 Institute of Medicine of the National Academies. WIC Food Packages: Time for a Change. Institute of Medicine, 2005.

10 United States Department of Agriculture: Food and Nutrition Service. WIC Studies: http://www.fns.usda.gov/ops/wic-studies. Date accessed: February 2015.

11 The RAMESES Project. Quality Standards for Realist Synthesis (for researchers and peer-reviewers): http://www.ramesesproject.org/media/RS_qual_standards_researchers.pdf. Date accessed: November 2014.

12 Wong G. Special Invited Editorial: Getting Started With Realist Research, *International Journal of Qualitative Methods* 2015;14.

13 Thomas J, Harden A. Methods for the thematic synthesis of qualitative research in systematic reviews, *BMC Medical Research Methodology* 2008;8:45.

14 Ford JA, Wong G, Jones AP, et al. Access to primary care for socioeconomically disadvantaged older people in rural areas: a realist review, *BMJ Open* 2016;6.

15 Department of Health. Healthy Start: Retailer research summary. London, 2012.

16 Hills D, Child C, Junge K, Wilkinson E, Sullivan F. Healthy Start: Rapid evaluation of early impact on beneficiaries, health professionals, retailers and contractors. London: Symbia/Tavistock Institute, 2006.

For peer review only

17 Andreyeva T, Middleton AE, Long MW, et al. Food retailer practices, attitudes and beliefs about the supply of healthy foods, *Public Health Nutr* 2011;14:1024-31.

18 Andreyeva T, Luedicke J, Henderson KE, et al. Grocery store beverage choices by participants in federal food assistance and nutrition programs, *Am J Prev Med* 2012;43:411-8.

19 Andreyeva T, Luedicke J, Tripp AS, et al. Effects of reduced juice allowances in food packages for the women, infants, and children program, *Pediatrics* 2013;131:919-27.

20 Andreyeva T, Luedicke J. Federal food package revisions: effects on purchases of whole-grain products, *Am J Prev Med* 2013;45:422-9.

21 Andreyeva T, Luedicke J, Henderson KE, et al. The positive effects of the revised milk and cheese allowances in the special supplemental nutrition program for women, infants, and children, *Journal of the Academy of Nutrition & Dietetics* 2014;114:622-30.

22 Andreyeva T, Luedicke J. Incentivizing fruit and vegetable purchases among participants in the Special Supplemental Nutrition Program for Women, Infants, and Children, *Public Health Nutr* 2015;18:33-41.

23 Ayala GX, Laska MN, Zenk SN, et al. Stocking characteristics and perceived increases in sales among small food store managers/owners associated with the introduction of new food products approved by the Special Supplemental Nutrition Program for Women, Infants, and Children, *Public Health Nutr* 2012;15:1771-9.

24 Bailey LB, O'Farrell-Ray B, Mahan CS, et al. Vitamin B6, iron and folacin status of pregnant women, *Nutr Res* 1983;3:783-93.

25 Bertmann FM, Barroso C, Ohri-Vachaspati P, et al. Women, infants, and children cash value voucher (CVV) use in Arizona: a qualitative exploration of barriers and strategies related to fruit and vegetable purchases, *Journal of Nutrition Education & Behavior* 2014;46:S53-8.

26 Black MM, Hurley KM, Oberlander SE, et al. Participants' comments on changes in the revised special supplemental nutrition program for women, infants, and children food packages: the Maryland food preference study, *J Am Diet Assoc* 2009;109:116-23.

27 Chen DY, Gazmararian JA. Impact of personal preference and motivation on fruit and vegetable consumption of WIC-participating mothers and children in Atlanta, GA, *Journal of Nutrition Education & Behavior* 2014;46:62-7.

28 Endres J, Dunning S, Poon SW, et al. Older pregnant women and adolescents: nutrition data after enrollment in WIC, *J Am Diet Assoc* 1987;87:1011.

29 Endres JM, Sawicki M, Casper JA. Dietary assessment of pregnant women in a supplemental food program. *J Am Diet Assoc* 1981;79:121-6.

30 Ettienne-Gittens R, McKyer ELJ, Odum M, et al. Rural versus Urban Texas WIC participants' fruit and vegetable consumption, *Am J Health Behav* 2013;37:130-40.

For peer review only

31 Farrior ES, Ruwe CH. Women, infants and children program. Prenatal participation and dietary intakes, *Nutr Res* 1987;7:451-549.

32 Gittelsohn J, Laska MN, Andreyeva T, et al. Small retailer perspectives of the 2009 women, infants and children program food package changes, *Am J Health Behav* 2012;36:655-65.

33 Herman DR, Harrison GG, Afifi AA, et al. The Effect of the WIC Program on Food Security Status of Pregnant, First-Time Participants, *Family Economics & Nutrition Review* 2004;16:21.

34 Herman DR, Harrison GG, Jenks E. Choices made by low-income women provided with an economic supplement for fresh fruit and vegetable purchase, *J Am Diet Assoc* 2006;106:740-4.

35 Herman DR, Harrison GG, Afifi AA, et al. Effect of a targeted subsidy on intake of fruits and vegetables among low-income women in the Special Supplemental Nutrition Program for Women, Infants, and Children, *Am J Public Health* 2008;98:98-105.

36 Horswill LJ, Yap C. Consumption of foods from the WIC food packages of Chinese prenatal patients on the US west coast, *J Am Diet Assoc* 1999;99:1549-53.

37 Kong A, Odoms-Young A, Schiffer LA, et al. Racial/ethnic differences in dietary intake among WIC families prior to food package revisions, *Journal of Nutrition Education & Behavior* 2013;45:39-46.

38 Kong A, Odoms-Young A, Schiffer LA, et al. The 18-month impact of special supplemental nutrition program for women, infants, and children food package revisions on diets of recipient families, *Am J Prev Med* 2014;46:543-51.

39 Meiqari L, Torre L, Gazmararian JA. Exploring the Impact of the New WIC Food Package on Low-Fat Milk Consumption Among WIC Recipients: A Pilot Study, *Journal of Health Care for the Poor & Underserved* 2015;26:712-25.

40 Nestor B, McKenzie J, Hasan N, et al. Client satisfaction with the nutrition education component of the California WIC program, *J Nutr Educ* 2001;33:83-94.

41 Odoms-Young A, Kong A, Schiffer LA, et al. Evaluating the initial impact of the revised Special Supplemental Nutrition Program for Women, Infants, and Children (WIC) food packages on dietary intake and home food availability in African-American and Hispanic families, *Public Health Nutr* 2014;17:83-93.

42 Pehrsson PR, Moser-Veillon PB, Sims LS, et al. Postpartum iron status in nonlactating participants and nonparticipants in the Special Supplemental Nutrition Program for Women, Infants, and Children, *Am J Clin Nutr* 2001;73:86-92.

43 Reyes NR, Klotz AA, Herring SJ. A qualitative study of motivators and barriers to healthy eating in pregnancy for low-income, overweight, African-American mothers, *Journal of the Academy of Nutrition & Dietetics* 2013;113:1175-81.

For peer review only

44 Rush D, Sloan NL, Leighton J, et al. The National WIC Evaluation: evaluation of the Special Supplemental Food Program for Women, Infants, and Children. V. Longitudinal study of pregnant women, *Am J Clin Nutr* 1988;48:439-83.

45 Rush D, Kurzon MR, Seaver WB, et al. The National WIC Evaluation: evaluation of the Special Supplemental Food Program for Women, Infants, and Children. VII. Study of food expenditures, *Am J Clin Nutr* 1988;48:512-9.

46 Swensen AR, Harnack LJ, Ross JA. Nutritional assessment of pregnant women enrolled in the Special Supplemental Program for Women, Infants, and Children (WIC), *J Am Diet Assoc* 2001;101:903-8.

47 Treiman K, Freimuth V, Damron D, et al. Attitudes and behaviors related to fruits and vegetables among low-income women in the WIC program, *J Nutr Educ Behav* 1996;28:149-56.

48 Watts V, Rockett H, Baer H, et al. Assessing diet quality in a population of low-income pregnant women: a comparison between Native Americans and whites, *Maternal & Child Health Journal* 2007;11:127-36.

49 Whaley SE, Ritchie LD, Spector P, et al. Revised WIC food package improves diets of WIC families, *Journal of Nutrition Education & Behavior* 2012;44:204-9.

50 Wunderlich SM, Hongu NK, Courter A, et al. Nutrient intake and nutritional status of low-income pregnant women, *Topics in Clinical Nutrition* 1996;12:66-74.

51 Thow AM, Downs S, Jan S. A systematic review of the effectiveness of food taxes and subsidies to improve diets: Understanding the recent evidence, *Nutr Rev* 2014;72:551-65.

52 Purnell JQ, Gernes R, Stein R, et al. A systematic review of financial incentives for dietary behavior change, *Journal of the Academy of Nutrition and Dietetics* 2014;114:1023-35.

53 An R. Effectiveness of subsidies in promoting healthy food purchases and consumption: a review of field experiments, *Public Health Nutr* 2013;16:1215-28.

54 Black AP, Brimblecombe J, Eyles H, et al. Food subsidy programs and the health and nutritional status of disadvantaged families in high income countries: a systematic review, *BMC Public Health* 2012;12:1099-122.

55 Abraham C, Michie S. A Taxonomy of Behavior Change Techniques Used in Interventions, *Health Psychol* 2008;27:379-87.

56 Oxford Dictionaries: http://www.oxforddictionaries.com/. Date accessed: June 2016.

57 Adams J, Mytton O, White M, et al. Why Are Some Population Interventions for Diet and Obesity More Equitable and Effective Than Others? The Role of Individual Agency, *PLoS Med* 2016;13:e1001990.

For peer review only

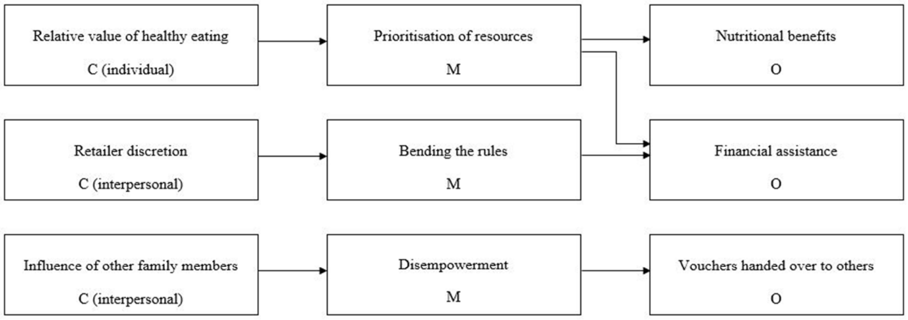

Summary of programme theories about how low-income pregnant women use Healthy Start vouchers

275x107mm (96 x 96 DPI)

For peer review only

Supplementary file 1. Characteristics of studies included in review (n=38)

**Full citation**	**Intervention (country)**	**Study design and data collection methods**	**Contribution to data extraction (C/M/O) and synthesis (programme theories 1-3)**					
			**C**	**M**	**O**	**1**	**2**	**3**
Department of Health. Healthy Start: Retailer research summary. London, 2012.	Healthy Start (UK)	Methods unclear	✓	✓			✓	
Hills D, Child C, Junge K, Wilkinson E, Sullivan F. Healthy Start: Rapid evaluation of early impact on beneficiaries, health professionals, retailers and contractors. London: Symbia/Tavistock Institute, 2006.	Healthy Start (UK)	Rapid evaluation; quantitative surveys and qualitative feedback		✓	✓		✓	
Lucas PJ, Jessiman T, Cameron A, et al. Healthy Start Vouchers Study: The Views and Experiences of Parents, Professionals and Small Retailers in England. Bristol: University of Bristol, 2013.	Healthy Start (UK)	Qualitative study; in-depth interviews	✓	✓	✓	✓	✓	✓
McFadden A, Fox-Rushby J, Green JM, et al. Healthy Start: Understanding the use of vouchers and vitamins. Dundee: University of Dundee, 2013.	Healthy Start (UK)	Focus groups; online consultations; workshops and telephone interviews	✓	✓	✓	✓		
Andreyeva T, Middleton AE, Long MW, et al. Food retailer practices, attitudes and beliefs about the supply of healthy foods, *Public Health Nutr* 2011;14:1024-31.	WIC (US)	Before/after study; structured interviews to assess perceived customer demand for healthy foods (mostly quantitative)		✓	✓		✓	
Andreyeva T, Luedicke J, Henderson KE, et al. Grocery store beverage choices by participants in federal food assistance and nutrition programs, *Am J Prev Med* 2012;43:411-8.	WIC (US)	Cross-sectional study; electronic sales data from WIC retailers			✓			
Andreyeva T, Luedicke J, Tripp AS, et al. Effects of reduced juice allowances in food packages for the women, infants, and children program, *Pediatrics* 2013;131:919-27.	WIC (US)	Before/after study; electronic sales data from WIC retailers			✓			

For peer review only

Full citation	Intervention (country)	Study design and data collection methods	Contribution to data extraction (C/M/O) and synthesis (programme theories 1-3)					
			C	M	O	1	2	3
Andreyeva T, Luedicke J. Federal food package revisions: effects on purchases of whole-grain products, *Am J Prev Med* 2013;45:422-9.	WIC (US)	Before/after study; electronic sales data from WIC retailers			✓			
Andreyeva T, Luedicke J, Henderson KE, et al. The positive effects of the revised milk and cheese allowances in the special supplemental nutrition program for women, infants, and children, *Journal of the Academy of Nutrition & Dietetics* 2014;114:622-30.	WIC (US)	Before/after study; electronic sales data from WIC retailers			✓			
Andreyeva T, Luedicke J. Incentivizing fruit and vegetable purchases among participants in the Special Supplemental Nutrition Program for Women, Infants, and Children, *Public Health Nutr* 2015;18:33-41.	WIC (US)	Before/after study; electronic sales data from WIC retailers			✓			
Ayala GX, Laska MN, Zenk SN, et al. Stocking characteristics and perceived increases in sales among small food store managers/owners associated with the introduction of new food products approved by the Special Supplemental Nutrition Program for Women, Infants, and Children, *Public Health Nutr* 2012;15:1771-9.	WIC (US)	Cross-sectional study; in-depth interviews to assess perceived changes in sales of WIC foods (quantitative data from closed questions)			✓			
Bailey LB, O'Farrell-Ray B, Mahan CS, et al. Vitamin B6, iron and folacin status of pregnant women, *Nutr Res* 1983;3:783-93.	WIC (US)	Cross-sectional study; dietary assessment using 24 hour recalls; blood tests for nutrient status			✓			
Bertmann FM, Barroso C, Ohri-Vachaspati P, et al. Women, infants, and children cash value voucher (CVV) use in Arizona: a qualitative exploration of barriers and strategies related to fruit and vegetable purchases, *Journal of Nutrition Education & Behavior* 2014;46:S53-8.	WIC (US)	Qualitative study; focus groups to explore attitudes and behaviours related to FV voucher use	✓		✓			

For peer review only

Full citation	Intervention (country)	Study design and data collection methods	Contribution to data extraction (C/M/O) and synthesis (programme theories 1-3)					
			C	M	O	1	2	3
Black MM, Hurley KM, Oberlander SE, et al. Participants' comments on changes in the revised special supplemental nutrition program for women, infants, and children food packages: the Maryland food preference study, *J Am Diet Assoc* 2009;109:116-23.	WIC (US)	Cross-sectional study; questionnaire to assess current and anticipated consumption; focus groups to explore reactions to proposed changes to WIC package			✓			
Chen DY, Gazmararian JA. Impact of personal preference and motivation on fruit and vegetable consumption of WIC-participating mothers and children in Atlanta, GA, *Journal of Nutrition Education & Behavior* 2014;46:62-7.	WIC (US)	Cross-sectional study; questionnaire to assess FV consumption			✓			
Endres J, Dunning S, Poon SW, et al. Older pregnant women and adolescents: nutrition data after enrollment in WIC, *J Am Diet Assoc* 1987;87:1011.	WIC (US)	Cross-sectional study; dietary assessment using 24 hour recalls			✓			
Endres JM, Sawicki M, Casper JA. Dietary assessment of pregnant women in a supplemental food program. *J Am Diet Assoc* 1981;79:121-6.	WIC (US)	Cross-sectional study; dietary assessment using 24 hour recalls			✓			
Ettienne-Gittens R, McKyer ELJ, Odum M, et al. Rural versus Urban Texas WIC participants' fruit and vegetable consumption, *Am J Health Behav* 2013;37:130-40.	WIC (US)	Cross-sectional study; questionnaire to assess FV consumption and variety			✓			
Farrior ES, Ruwe CH. Women, infants and children program. Prenatal participation and dietary intakes, *Nutr Res* 1987;7:451-549.	WIC (US)	Before/after study; dietary assessment using 24 hour recalls			✓			
Gittelsohn J, Laska MN, Andreyeva T, et al. Small retailer perspectives of the 2009 women, infants and children program food package changes, *Am J Health Behav* 2012;36:655-65.	WIC (US)	Qualitative study; in-depth interviews with small store owners and managers	✓	✓	✓		✓	

For peer review only

**Full citation**	**Intervention (country)**	**Study design and data collection methods**	**Contribution to data extraction (C/M/O) and synthesis (programme theories 1-3)**					
			**C**	**M**	**O**	**1**	**2**	**3**
Herman DR, Harrison GG, Afifi AA, et al. The Effect of the WIC Program on Food Security Status of Pregnant, First-Time Participants, *Family Economics & Nutrition Review* 2004;16:21.	WIC (US)	Longitudinal study; US Food Security Survey Module; qualitative interviews on use of WIC food package as income transfer (presented as quantitative data)			✓	✓		
Herman DR, Harrison GG, Jenks E. Choices made by low-income women provided with an economic supplement for fresh fruit and vegetable purchase, *J Am Diet Assoc* 2006;106:740-4.	WIC (US)	Cross-sectional study; data from retailers on FV purchases			✓			
Herman DR, Harrison GG, Afifi AA, et al. Effect of a targeted subsidy on intake of fruits and vegetables among low-income women in the Special Supplemental Nutrition Program for Women, Infants, and Children, *Am J Public Health* 2008;98:98-105.	WIC (US)	Longitudinal study; dietary assessment using 24 hour recalls			✓			
Horswill LJ, Yap C. Consumption of foods from the WIC food packages of Chinese prenatal patients on the US west coast, *J Am Diet Assoc* 1999;99:1549-53.	WIC (US)	Cross-sectional study; dietary assessment using FFQ	✓		✓			
Kong A, Odoms-Young A, Schiffer LA, et al. Racial/ethnic differences in dietary intake among WIC families prior to food package revisions, *Journal of Nutrition Education & Behavior* 2013;45:39-46.	WIC (US)	Cross-sectional study; dietary assessment using 24 hour recalls	✓		✓			
Kong A, Odoms-Young A, Schiffer LA, et al. The 18-month impact of special supplemental nutrition program for women, infants, and children food package revisions on diets of recipient families, *Am J Prev Med* 2014;46:543-51.	WIC (US)	Longitudinal study; dietary assessment using 24 hour recalls (baseline data from Kong et al. 2013)	✓		✓			

For peer review only

Full citation	Intervention (country)	Study design and data collection methods	Contribution to data extraction (C/M/O) and synthesis (programme theories 1-3)					
			C	M	O	1	2	3
Meiqari L, Torre L, Gazmararian JA. Exploring the Impact of the New WIC Food Package on Low-Fat Milk Consumption Among WIC Recipients: A Pilot Study, *Journal of Health Care for the Poor & Underserved* 2015;26:712-25.	WIC (US)	Before/after study; questionnaire to assess primary consumption of low-fat milk (yes/no)			✓			
Nestor B, McKenzie J, Hasan N, et al. Client satisfaction with the nutrition education component of the California WIC program, *J Nutr Educ* 2001;33:83-94.	WIC (US)	Cross-sectional study; client satisfaction survey; focus groups to assess client experiences and satisfaction	✓	✓	✓			
Odoms-Young A, Kong A, Schiffer LA, et al. Evaluating the initial impact of the revised Special Supplemental Nutrition Program for Women, Infants, and Children (WIC) food packages on dietary intake and home food availability in African-American and Hispanic families, *Public Health Nutr* 2014;17:83-93.	WIC (US)	Longitudinal study; dietary assessment using 24 hour recalls (baseline data from Kong et al. 2013) and questionnaire to assess home food availability			✓			
Pehrsson PR, Moser-Veillon PB, Sims LS, et al. Postpartum iron status in nonlactating participants and nonparticipants in the Special Supplemental Nutrition Program for Women, Infants, and Children, *Am J Clin Nutr* 2001;73:86-92.	WIC (US)	Longitudinal study; iron status using FFQ and blood tests			✓			
Reyes NR, Klotz AA, Herring SJ. A qualitative study of motivators and barriers to healthy eating in pregnancy for low-income, overweight, African-American mothers, *Journal of the Academy of Nutrition & Dietetics* 2013;113:1175-81.	WIC (US)	Qualitative study; semi-structured interviews to explore motivators and barriers to healthy eating	✓	✓	✓			✓

For peer review only

**Full citation**	**Intervention (country)**	**Study design and data collection methods**	**Contribution to data extraction (C/M/O) and synthesis (programme theories 1-3)**					
			**C**	**M**	**O**	**1**	**2**	**3**
Rush D, Sloan NL, Leighton J, et al. The National WIC Evaluation: evaluation of the Special Supplemental Food Program for Women, Infants, and Children. V. Longitudinal study of pregnant women, *Am J Clin Nutr* 1988;48:439-83.	WIC (US)	Before/after study; food expenditure assessed by recall (and diary)			✓			
Rush D, Kurzon MR, Seaver WB, et al. The National WIC Evaluation: evaluation of the Special Supplemental Food Program for Women, Infants, and Children. VII. Study of food expenditures, *Am J Clin Nutr* 1988;48:512-9.	WIC (US)	Longitudinal study; dietary assessment using 24 hour recalls			✓			
Swensen AR, Harnack LJ, Ross JA. Nutritional assessment of pregnant women enrolled in the Special Supplemental Program for Women, Infants, and Children (WIC), *J Am Diet Assoc* 2001;101:903-8.	WIC (US)	Cross-sectional study; dietary assessment using FFQ; blood tests for iron status			✓			
Treiman K, Freimuth V, Damron D, et al. Attitudes and behaviors related to fruits and vegetables among low-income women in the WIC program, *J Nutr Educ Behav* 1996;28:149-56.	WIC (US)	Qualitative study; focus groups to explore attitudes and behaviours related to FV; central location intercept interviews (questionnaires)	✓	✓	✓	✓		✓
Watts V, Rockett H, Baer H, et al. Assessing diet quality in a population of low-income pregnant women: a comparison between Native Americans and whites, *Maternal & Child Health Journal* 2007;11:127-36.	WIC (US)	Cross-sectional study; FFQ to assess diet quality			✓			
Whaley SE, Ritchie LD, Spector P, et al. Revised WIC food package improves diets of WIC families, *Journal of Nutrition Education & Behavior* 2012;44:204-9.	WIC (US)	Cross-sectional study; questionnaire to assess usual dietary habits and changes compared to 6 months earlier			✓			

For peer review only

**Full citation**	**Intervention (country)**	**Study design and data collection methods**	**Contribution to data extraction (C/M/O) and synthesis (programme theories 1-3)**					
			**C**	**M**	**O**	**1**	**2**	**3**
Wunderlich SM, Hongu NK, Courter A, et al. Nutrient intake and nutritional status of low-income pregnant women, *Topics in Clinical Nutrition* 1996;12:66-74.	WIC (US)	Cross-sectional study; dietary assessment using 24 hour recalls			✓			

WIC = Special Supplemental Program for Women, Infants, and Children; FV = fruits and vegetables; Programme theories: 1. Prioritisation of resources; 2. Bending the rules; 3. Disempowerment

For peer review only

List of items to be included when reporting a realist synthesis

TITLE			Page
1		In the title, identify the document as a realist synthesis or review	1
ABSTRACT			
2		While acknowledging publication requirements and house style, abstracts should ideally contain brief details of: the study's background, review question or objectives; search strategy; methods of selection, appraisal, analysis and synthesis of sources; main results; and implications for practice.	2
INTRODUCTION			
3	Rationale for review	Explain why the review is needed and what it is likely to contribute to existing understanding of the topic area.	4
4	Objectives and focus of review	State the objective(s) of the review and/or the review question(s). Define and provide a rationale for the focus of the review.	4-5
METHODS			
5	Changes in the review process	Any changes made to the review process that was initially planned should be briefly described and justified.	NA
6	Rationale for using realist synthesis	Explain why realist synthesis was considered the most appropriate method to use.	4
7	Scoping the literature	Describe and justify the initial process of exploratory scoping of the literature.	5-6
8	Searching processes	While considering specific requirements of the journal or other publication outlet, state and provide a rationale for how the iterative searching was done. Provide details on all the sources accessed for information in the review. Where searching in electronic databases has taken place, the details should include, for example, name of database, search terms, dates of coverage and date last searched. If individuals familiar with the relevant literature and/or topic area were contacted, indicate how they were identified and selected.	6
9	Selection and appraisal of documents	Explain how judgements were made about including and excluding data from documents, and justify these.	7-8
10	Data extraction	Describe and explain which data or information were extracted from the included documents and justify this selection.	8
11	Analysis and synthesis processes	Describe the analysis and synthesis processes in detail. This section should include information on the constructs analyzed and describe the analytic process.	9
RESULTS			
12	Document flow diagram	Provide details on the number of documents assessed for eligibility and included in the review with reasons for exclusion at each stage as well as an indication of their source of origin (for example, from searching databases, reference lists and so on).	9 and 21

For peer review only

TITLE			Page
		You may consider using the example templates (which are likely to need modification to suit the data) that are provided.	
13	Document characteristics	Provide information on the characteristics of the documents included in the review.	9-11
14	Main findings	Present the key findings with a specific focus on theory building and testing.	11-13
DISCUSSION			
15	Summary of findings	Summarize the main findings, taking into account the review's objective(s), research question(s), focus and intended audience(s).	13
16	Strengths, limitations and future research directions	Discuss both the strengths of the review and its limitations. These should include (but need not be restricted to) (a) consideration of all the steps in the review process and (b) comment on the overall strength of evidence supporting the explanatory insights which emerged.<br><br>The limitations identified may point to areas where further work is needed.	13-14
17	Comparison with existing literature	Where applicable, compare and contrast the review's findings with the existing literature (for example, other reviews) on the same topic.	14-15
18	Conclusion and recommendations	List the main implications of the findings and place these in the context of other relevant literature. If appropriate, offer recommendations for policy and practice.	15
19	Funding	Provide details of funding source (if any) for the review, the role played by the funder (if any) and any conflicts of interests of the reviewers.	16

Source of this checklist:

http://www.equator-network.org/reporting-guidelines/rameses-publication-standards-realist-syntheses/

http://www.ramesesproject.org/Standards_and_Training_materials.php

http://bmcmedicine.biomedcentral.com/articles/10.1186/1741-7015-11-21#Tab1

Page numbers refer to pdf document created during submission process.

For peer review only

PRISMA 2009 Flow Diagram

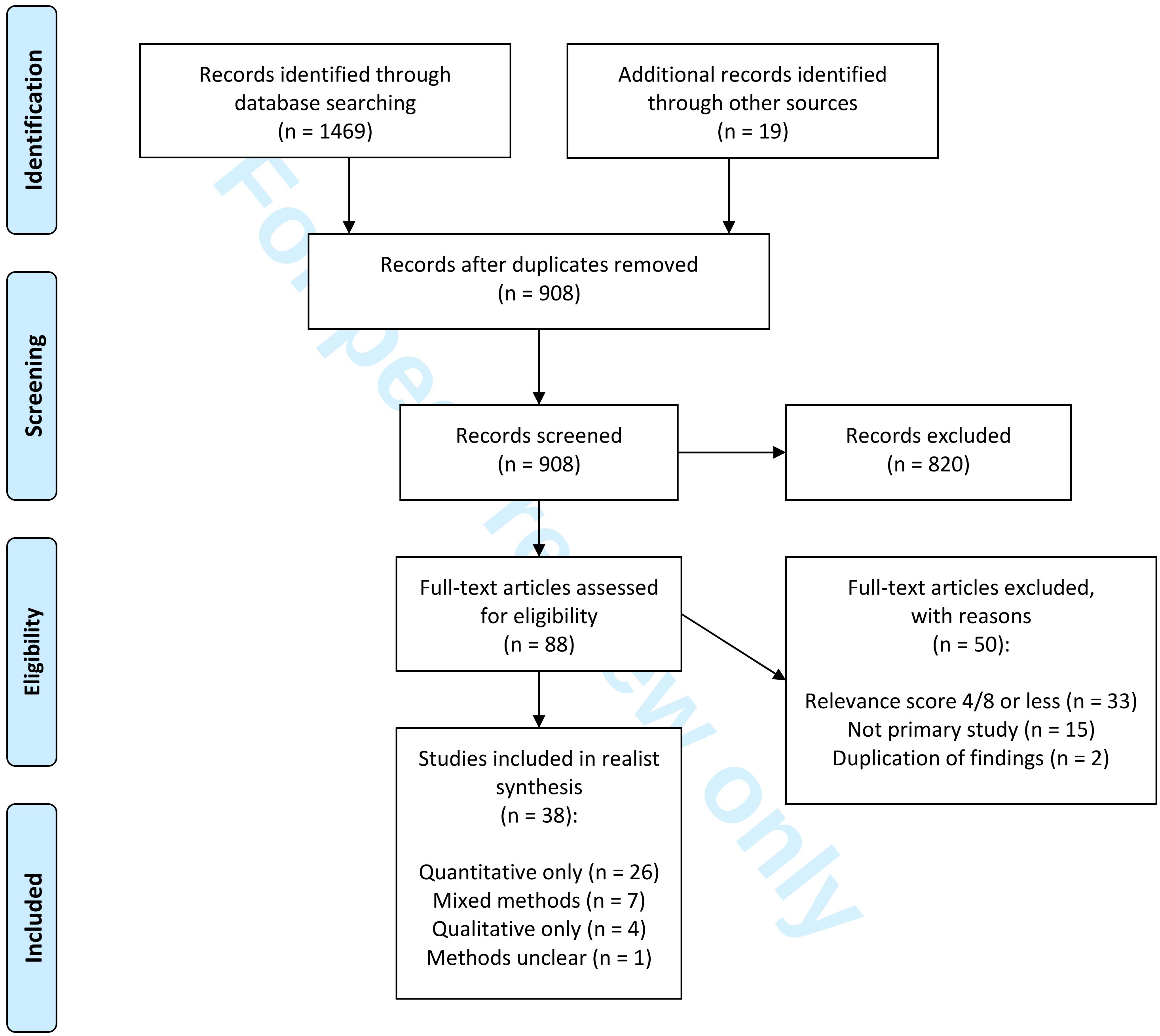

From: Moher D, Liberati A, Tetzlaff J, Altman DG, The PRISMA Group (2009). *P*referred *R*eporting *I*tems for *S*ystematic Reviews and *M*eta-*A*nalyses: The PRISMA Statement. PLoS Med 6(6): e1000097. doi:10.1371/journal.pmed1000097

For more information, visit www.prisma-statement.org.

For peer review only - http://bmjopen.bmj.com/site/about/guidelines.xhtml

BMJ Open

A realist review to explore how low-income pregnant women use food vouchers from the UK's Healthy Start programme

Journal:	*BMJ Open*
Manuscript ID	bmjopen-2016-013731.R3
Article Type:	Research
Date Submitted by the Author:	15-Dec-2016
Complete List of Authors:	Ohly, Heather; University of Central Lancashire, College of Health and Wellbeing<br>Crossland, Nicola; University of Central Lancashire, College of Health and Wellbeing<br>Dykes, Fiona; University of Central Lancashire, College of Health and Wellbeing<br>Lowe, Nicola; University of Central Lancashire, College of Health and Wellbeing<br>Hall-Moran, Victoria; University of Central Lancashire, College of Health and Wellbeing
**Primary Subject Heading**:	Public health
Secondary Subject Heading:	Nutrition and metabolism
Keywords:	pregnant, women, food, vouchers, realist, review

SCHOLARONE™ Manuscripts

For peer review only

Title of the article

A realist review to explore how low-income pregnant women use food vouchers from the UK's Healthy Start programme

Full name, postal address, email and telephone number of corresponding author:

Heather Ohly, Brook Building 215, College of Health and Wellbeing, University of Central Lancashire, Preston, UK

HOhly@uclan.ac.uk

07866 318014

Full name, department, institution, city and country of all co-authors:

Heather Ohly, College of Health and Wellbeing, University of Central Lancashire, Preston, UK

Nicola Crossland, College of Health and Wellbeing, University of Central Lancashire, Preston, UK

Fiona Dykes, College of Health and Wellbeing, University of Central Lancashire, Preston, UK

Nicola Lowe, College of Health and Wellbeing, University of Central Lancashire, Preston, UK

Victoria Hall Moran, College of Health and Wellbeing, University of Central Lancashire, Preston, UK

Word count, excluding title page, abstract, references, figures and tables:

5688 words

For peer review only

ABSTRACT

Objectives: To explore how low-income pregnant women use Healthy Start food vouchers, the potential impacts of the programme, which women might experience these impacts and why.

Design: A realist review.

Eligibility criteria for selecting studies: Primary or empirical studies (of any design) were included if they contributed relevant evidence or insights about how low-income women use food vouchers from the Healthy Start (UK) or the Special Supplemental Nutrition Program for Women, Infants and Children (US) programmes. The assessment of 'relevance' was deliberately broad to ensure that reviewers remained open to new ideas from a variety of sources of evidence.

Analysis: A combination of evidence synthesis and realist analysis techniques was used to modify, refine and substantiate programme theories, which were constructed as explanatory 'context – mechanism – outcome' (CMO) configurations.

Results: 38 primary studies were included in this review: four studies on Healthy Start and 34 studies on WIC. Two main outcome strands were identified: dietary improvements (intended) and financial assistance (unintended). Three evidence-informed programme theories were proposed to explain how aspects of context (and mechanisms) may generate these outcomes: the 'relative value' of healthy eating (prioritisation of resources); retailer discretion (pressure to 'bend the rules'); the influence of other family members (disempowerment).

Conclusions: This realist review suggests that some low-income pregnant women may use Healthy Start vouchers to increase their consumption of fruits and vegetables and plain cow's milk, whereas others may use them to reduce food expenditure and save money for other things.

Key words: pregnant, women, food, vouchers, realist, review

For peer review only

Strengths and limitations of this study:

- First study to articulate, develop and test programme theories about Healthy Start
- Inclusion of relevant studies from a similar programme in the US (WIC)
- Some evidence not transferable due to population and programme differences
- Insufficient evidence to link CMO configurations to sociodemographic and cultural characteristics

For peer review only

BACKGROUND

Healthy Start is the United Kingdom (UK) government's food voucher programme for low-income pregnant women and young children. It was introduced in 2006, after the Acheson Review drew attention to income as one of the major determinants of health (and nutrition) inequalities, and highlighted the importance of nutrition for women of childbearing age and their children.[1] The Committee on Medical Aspects of Food and Nutrition Policy (COMA) was asked by the government to review the longstanding Welfare Food Scheme, which was subsequently replaced by Healthy Start.[2]

Women are eligible for Healthy Start if they receive income-related benefits or child tax credit and an annual household income of £16,190 or less. Pregnant women aged under 18 are eligible regardless of their income. The weekly voucher values are: one voucher per week during pregnancy (£3.10); two vouchers per week for each baby under one year (£6.20) and one voucher per week for each child aged 1-4 years (£3.10). The vouchers can be exchanged for fruits and vegetables, plain cow's milk or infant formula. Retailers must be registered with the scheme to accept and claim payment for the vouchers. Healthy Start also offers free vitamins for eligible women and children, but there have been problems with uptake of vitamins.[3] Some areas offer free vitamins to all pregnant women and young children and the option of universalising Healthy Start vitamins remains under review (at the time of writing) by the Chief Medical Officer. Therefore, this review focused on the food voucher component of the programme and low-income pregnant women as the first beneficiaries.

Healthy Start aims to provide a "basic nutritional safety net" and to encourage "women and families to make positive nutritional choices affecting their longer term health" (page 4).[4] Therein lies an implicit programme assumption that women will use the vouchers to purchase target foods (ideally in greater quantities than they did before) leading to dietary improvements. The vouchers may have been conceived as a financial incentive for dietary improvements, although this was not explicit in policy documentation. Since the introduction of Healthy Start in 2006, there has been no robust evaluation of its impact on nutritional outcomes – despite recommendations published in an early scoping review[5] However, qualitative studies have indicated a range of experiences, motivations and perceived outcomes, with not all low-income women using the vouchers to improve their diets.[6, 7] This review was undertaken to explore how low-income pregnant women use Healthy Start

For peer review only

food vouchers, the potential impacts of the programme, which women might experience these impacts and why.

The realist approach was adopted because it is based on the understanding that different individuals or groups of individuals are likely to respond to any programme (or intervention) in different ways. It is a theory-driven approach that seeks to explore outcome patterns (or demi-regularities) and offer plausible explanations for how and why they occur. The purpose of realist synthesis is to "articulate underlying programme theories and then to interrogate the existing evidence to find out whether and where these theories are pertinent and productive" (page 74).[8] The stages of conducting a realist review tend to be iterative and overlapping – a gradual process of developing, testing and refining programme theories. Evidence may be obtained from studies of the programme itself, or more widely from similar programmes that are thought to work in similar ways. Reviewers may adapt and modify existing theoretical frameworks or 'middle-range' theories to help develop their own explanations. The unit of analysis is the programme theory (rather than the specific programme) and this can be considered at different levels of abstraction.

Programme theories are often constructed as 'context – mechanism – outcome' (CMO) configurations, and evidence is sought to substantiate the causal linkages. The logic of realist explanation is that outcomes (O) are caused by mechanisms (M), and mechanisms may (or may not) be 'triggered' in certain contexts (C).[8] Context refers to the pre-existing conditions into which the programme is introduced, and there are four layers of context: individual, interpersonal, institutional and infrastructural. Mechanisms are defined as the reasoning and reactions of individuals in response to the resources offered by the programme. A core principle of realism is that mechanisms generate outcomes – they are not a direct result of the programme.

This realist review aimed to explore the following questions:

1. How do low-income pregnant women use Healthy Start vouchers?
2. What are the intended and unintended outcomes of the programme?
3. What are the underlying mechanisms and how do variations in context influence (enable or constrain) these mechanisms?

For peer review only

A preliminary search confirmed the paucity of empirical studies on Healthy Start and we felt that additional sources of empirical evidence would be needed to explore these research questions. The most obvious source of potentially relevant evidence was the Special Supplemental Nutrition Program for Women, Infants and Children (WIC) in the United States (US). WIC is the only other national food subsidy programme for low-income women of childbearing age, including pregnant and postpartum women, and young children. It was first introduced in 1972 and revised in 2009 to reflect current dietary guidelines.[9] The WIC food package for pregnant women is different to Healthy Start, with 'maximum monthly allowances' for a range of foods (reduced-fat milk, whole grain cereals, eggs and pulses) and 'cash value vouchers' for fruits and vegetables. The other main programme difference is mandatory WIC nutrition education for all beneficiaries. There are also many contextual differences between the US and the UK, such as socio-demographic, cultural, geographical and political characteristics. Despite these differences, there are also likely to be similarities in terms of how low-income women respond to the programme (i.e. mechanisms).

METHODS

Study protocol and ethical approval

The protocol for this realist review was registered with the International Prospective Register of Systematic Reviews (PROSPERO 2014: CRD42014015050). There were no changes to the review process since this protocol was published. Ethical approval was obtained from the University of Central Lancashire Science, Technology, Engineering, Medicine and Health (STEMH) Ethics Committee in May 2015.

Programme theory development

This review is about a programme that already exists, with implicit theories and assumptions about how it works and what effects it may have on beneficiaries.[8] Therefore, we used a 'bottom up' approach to theory development in this review, as described by Shearn and Allmark (Realist Research Seminar Series, Sheffield Hallam University, 2016). In other words, we developed theories using information about the Healthy Start food voucher programme, rather than theorising at a more abstract level. Candidate theories (or initial, untested theories) were identified and prioritised using information derived from academic and grey literature on Healthy Start, an intervention mapping exercise, existing knowledge, creative thinking, consultations with stakeholders (in person and by email) and discussions

For peer review only

among the review team. The stakeholder group included six midwives, two academics and two public health practitioners, who all shared first hand experiences and insights about how the Healthy Start programme works in practice.

We developed candidate theories about how low-income pregnant women might experience Healthy Start, and what contextual factors might influence their reasoning and reactions (mechanisms) at each of the following stages: access to the programme starting at the first trimester antenatal appointment (e.g. issues around clear communication and understanding of eligibility criteria and entitlement); the application process (e.g. barriers and facilitators to successfully receiving the vouchers); whether and where women use the food vouchers (e.g. issues around convenience and stigmatisation); and how women use the food vouchers (e.g. to buy more of the target foods or to save money). We decided to prioritise the last stage of the programme pathway: once an eligible pregnant woman has received the food vouchers, how does she use them and why? This decision reflected the research priorities identified from the literature, the interests of the research team, and the time and resources available to conduct this review. The candidate theories we tested were proposed explanations for why women might use Healthy Start to improve their diets during pregnancy, such as motivations and values relating to health benefits. We also considered reasons why women might use their vouchers in alternative ways, including prioritisation of resources, pressure to bend the rules, and disempowerment.

Search strategy

Separate searches were conducted for Healthy Start and WIC:

Healthy Start – Studies were identified through manual, purposive, snowball and citation searches (January to March 2015). The search terms used initially were 'Healthy Start' and 'UK' because there is another programme called Healthy Start in the US, which aims to prevent infant mortality. More precise search terms were not needed due to the paucity of empirical studies and familiarity with the literature.

WIC – A broad search strategy was devised in collaboration with an Information Specialist in the Collaboration for Leadership in Applied Health Research and Care (CLAHRC) North West Coast. This strategy was adapted and run in six electronic databases in September 2015: MEDLINE, EMBASE, CINAHL, Open Grey, ETHOS and PubMed. Table 1 shows the search terms used in MEDLINE. No date or language restrictions were used. Reference lists

For peer review only

of included studies were checked for additional studies. An online list of WIC studies was also checked for additional studies.[10]

Table 1. Search strategy used in MEDLINE to identify WIC studies

#	Search terms	Results
1	WIC.tw.	1008
2	(nutrition or food or voucher or "nutrition program").tw.	377002
3	1 and 2	599
4	(Special* adj4 Supplement* adj4 Nutrit* adj4 Program* adj4 Women* adj4 Infant* adj4 Child*).tw.	415
5	3 or 4	688

Inclusion criteria

Primary or empirical studies (of any design) were included if they contributed relevant evidence or insights about how low-income women use food vouchers from the Healthy Start (UK) or WIC (US) programmes. An assessment of 'relevance' is essential in realist synthesis, to ensure that all included studies contribute to theory development, refinement and testing.[8, 11] In this review, the interpretation of 'relevance' was deliberately broad to ensure that reviewers remained open to new ideas from a variety of sources of evidence. A bespoke system was used to maintain a consistent and transparent approach. Table 2 shows the questions used to assess relevance. These questions were developed by the review team and finalised towards the end of the theory development stage, to ensure they reflected the candidate theories we wanted to test. Studies that scored 5/8 or more (based on the total number of yes answers) were included.

For peer review only

Table 2. Questions used to assess the relevance of primary studies

#	Question:
1	Do the research questions or study aims refer to Healthy Start or WIC?
2	Does the study focus on the food voucher (CVV or food package for WIC) component of the programme?
3	Does the study focus on beneficiaries (women who were receiving the vouchers) rather than eligibility status (women who were eligible to receive the vouchers)?
4	Does the sample include pregnant women?
5	If the sample does not include pregnant women, could some of the findings be generalizable to pregnant women?
6	Does the study report women's food or nutrient intakes (measured or perceived)?
7	Does the study provide any insights about how food vouchers are used?
8	Does the study provide any insights about which women may benefit most/least and why?

Study selection

Results from the WIC database searches were uploaded into RefWorks (web version; ProQuest; Michigan, US) and screened using titles and abstracts. Studies that appeared to meet the inclusion criteria were obtained as full text articles. Studies for which insufficient information was available to determine relevance were also obtained as full text articles. The full text screening process was fully documented using Microsoft Excel 2013 (version 15.0.4815.1001; Microsoft Corporation), including assessments of relevance and reasons for exclusions. The same criteria were applied to studies of Healthy Start. Study selection was completed by the lead reviewer (HO) and double checked by a second reviewer (VM). Any disagreements were resolved by discussion.

Data extraction

Quantitative data on women's nutritional outcomes were extracted using bespoke tables in Microsoft Word 2013 (version 15.0.4815.1001; Microsoft Corporation). Other non-relevant data were not extracted. Qualitative data, textual descriptions of findings, and author interpretations were extracted using MAXQDA 11 (version 11; VERBI Software GmbH; Berlin). A coding system was created with three main headings: context, mechanisms and outcomes. Subheadings were added deductively (based on candidate theories) and inductively

For peer review only

(as new themes emerged from the data). Data extraction was completed by the lead reviewer (HO) and a sample was double checked by a second reviewer (NC).

Quality appraisal

Studies were not formally appraised at the data extraction stage, as would be the case in traditional systematic reviews. Instead, an assessment of 'rigour' was used to judge the credibility and trustworthiness of the evidence as it was integrated into the analysis and synthesis.[8, 11] This assessment was not scored because weaker studies were still included, but it meant that methodological limitations were acknowledged and study findings were not over-interpreted. Table 3 shows the questions used to assess rigour. Quality appraisal was completed by the lead reviewer (HO) and double checked by second reviewers (NL/VM).

Table 3. Questions used to assess the rigour of primary studies

#	Question:
1	Are the study methods clearly reported (including study design, recruitment, data collection and analysis)?
2	Are the study methods appropriate to answer the research questions?
3	Are the sample characteristics reported to enable judgements about generalisability?
4	Are the study findings and conclusions supported by raw data?
5	Are the study limitations acknowledged and clearly reported?

Analysis and synthesis

This process involved gradual and iterative theory development, whereby evidence from primary studies was used to modify, refine and substantiate programme theories about how low-income pregnant women use Healthy Start vouchers, in what circumstances, and why. Theories were constructed as explanatory 'context – mechanism – outcome' (CMO) configurations, usually by starting with the outcome and working backwards to determine "what caused it (the mechanism) and under what contexts was the mechanism triggered".[12] The main focus of the analysis was searching for evidence to support and refute the proposed causal linkages between context, mechanisms and outcomes. A combination of evidence synthesis and realist analysis techniques was used:

For peer review only

1. Narrative synthesis of quantitative data on women's nutritional outcomes; meta-analysis was not appropriate due to heterogeneity of study designs and data collection methods (and was beyond the scope of this review).
2. Thematic synthesis of qualitative data, by creating codes and themes (as described under data extraction) and then 'going beyond' the interpretations of the original studies to generate new understandings or hypotheses.[13]
3. Creative theorising or 'retroduction' by the lead reviewer (HO) in collaboration with the review team and the stakeholder group. This involved in-depth reflection and discussions (throughout the review) about the underlying causes of outcome patterns, at the level of generative mechanisms and explanatory context. The data from included studies did not always provide such in-depth insights and explanations. Where individual extracts of data only supported part of the CMO configuration, it was necessary to make logical inferences about the complete causal pathways and explanations.[14]

RESULTS

Search results and study characteristics

A total of 908 records were identified through the two separate searches. After screening titles and abstracts, 88 records were obtained in full text format. Fifty full text articles were excluded based on the assessment of relevance (n=33) or because they were not primary studies (n=15) or the findings were duplicated (n=2). Therefore, 38 primary studies were included in this review: four UK studies on Healthy Start[6, 7, 15, 16] and 34 US studies on WIC[17-50] (see PRISMA Flow Diagram and online supplementary file 1).

Identification of outcomes and supporting evidence

Two main outcome strands emerged during the theory development stage and were further substantiated using evidence from primary studies:

1. Women use vouchers to increase consumption of target foods – dietary improvements.
2. Women use vouchers to reduce food expenditure – financial assistance.

For the purposes of this review, we have assumed that strand 1 is the intended outcome of the programme and strand 2 is an unintended outcome. This was not explicit in policy documentation, but there were references to dietary improvements, which were thought to be

For peer review only

achieved by enabling low-income women to access healthier foods and encouraging positive nutritional choices.[4] The included studies provided support for both outcome strands. The next section provides an overview of the available evidence on women's outcomes (intended and unintended) and highlights the relative contribution of evidence from Healthy Start and WIC studies. It also helps to illustrate how we worked backwards from outcomes to identify generative mechanisms and related aspects of context.

A total of 25 studies reported women's nutritional outcomes: three studies on Healthy Start and 22 studies on WIC. The Healthy Start studies reported perceived outcomes only; some women said they consumed more cow's milk, fruits and vegetables after receiving Healthy Start vouchers,[6, 7, 16] whereas other women said the vouchers "freed up money to do other things" and "helped them to manage better financially" (page 59).[7] The WIC studies were published between 1981 and 2015, but the most useful data was extracted from two studies comparing women's diets before and after the 2009 WIC revisions, when the 'cash value vouchers' for fruits and vegetables were introduced (there was no allowance for fresh fruits and vegetables before 2009). A longitudinal study of African American and Hispanic women from WIC clinics in Chicago (n=273) found significant dietary improvements for Hispanic mothers, who reported consuming more fruit, more reduced fat milk less whole milk and less saturated fat (all $p<0.05$).[39] African American mothers reported consuming less whole milk ($p=0.02$) but no other changes were statistically significant.[41] There were no sustained dietary improvements in either group compared to baseline at 18 months.[38] A cross-sectional study comparing two random samples of WIC participants in California (both 80% Hispanic) found that women assessed six months after the changes (n=2996) reported consuming significantly more whole grains, reduced-fat milk and vegetables, and less whole milk, compared to women assessed before the changes (n=3004) (all $p<0.001$).[49]

Five studies reported electronic sales data from WIC retailers (one large supermarket chain) in New England, which showed that women's purchasing patterns shifted towards items provided in the WIC package after the 2009 revisions – fruits and vegetables, reduced-fat milk, brown rice, whole grain cereals and bread replaced less nutritious options.[18-22] One study (n=2137) showed that, while total spending on fruits and vegetables increased between 2009 and 2010 ($p<0.001$), up to 13% fewer purchases were made using non-WIC funds.[22] This implies that some women 'substituted' the method of payment, rather than using WIC to increase the amount of fruits and vegetables purchased. None of these studies reported

For peer review only - http://bmjopen.bmj.com/site/about/guidelines.xhtml

sample characteristics such as ethnicity. Finally, a mixed methods study of Hispanic and African American pregnant women (n=313) found that two thirds of women reported using WIC vouchers to reduce food expenditure.[33] The money they saved was used to purchase items for the unborn baby, other foods, and for bills and emergencies.

These findings suggest that food vouchers may lead to dietary improvements for some, but not all women. This may be because some women use the vouchers to pay for foods they would previously have bought using cash. The WIC studies described above were not representative of ethnic groups in the UK, and the samples included mothers as well as pregnant women. However, these studies provide much needed evidence on the potential impact of food vouchers for low-income women, which was not available from the Healthy Start literature alone.

Evidence-informed programme theories

This section presents three evidence-informed programme theories, which help to explain why different women receive the same Healthy Start vouchers and yet experience different outcomes, due to variations in context and mechanisms. Figure 1 illustrates the key aspects of context, mechanisms and outcomes identified and the proposed causal pathways linking them together. These causal pathways are explained as CMO configurations and illustrated using quotations from included studies under each theory. Although low income is clearly an important aspect of context, we have not included it in our programme theories because it applies to all women who receive Healthy Start vouchers (apart from under 18s). As the programme theories explain, some women may achieve dietary improvements despite low income, and other women may not – this divergence depends on other aspects of context.

1. Prioritisation of resources

Women living on low incomes must constantly prioritise how they spend their money. Food vouchers may be considered as one part of the household resources and decisions must be made about how best to use the vouchers. A key aspect of individual level context is the ‘relative value’ of healthy eating (compared to other things women value), which can lead women to prioritise in different ways. Context is not static and women’s values may change over time, so we propose that some women may fluctuate between the mechanisms (ways of prioritising) outlined in these two contrasting CMO configurations.

For peer review only

Women who value healthy eating and aspire to eat well during pregnancy [context][47] are more likely to perceive Healthy Start vouchers as an opportunity to achieve health benefits for themselves and their unborn baby [mechanism].[6] The vouchers alleviate the financial barrier associated with healthy eating and make healthy foods seem more affordable [mechanism].[6] Therefore, women who value healthy eating are more likely to prioritise healthy eating [mechanism] and use Healthy Start vouchers to increase consumption of target foods – fruits and vegetables or cow's milk [outcome].[6]

> "I have them at Asda when I do my shop, and I think how many vouchers I've got and I buy the veg that I have the vouchers for. I suppose if I didn't have the vouchers, I would just pick out the little things. I don't think if I didn't have the vouchers I'd buy half as much, no." (Mother, UK; page 50)[6]

Alternatively, women may value healthy eating less than other things they want or need to spend money on, which are considered more important or urgent [context].[6, 7] They are more likely to perceive Healthy Start vouchers as a way to save money, which can be redirected and prioritised in other ways [mechanism].[6, 7] These women are more likely to use Healthy Start vouchers to deduct money from the shopping bill, with no increase in consumption of target foods [outcome].[6, 7, 33]

> "Women are often in a dilemma about whether they should or shouldn't eat healthy foods because something else is needed more. Their own health and maybe the health of their younger children are on the back burner because something else is more pressing." (Midwife, UK; page 35)[6]

2. Bending the rules

The Healthy Start voucher exchange system relies on registered retailers to verify (visually) at the checkout that women have selected appropriate amounts of appropriate items – fruits and vegetables, plain cow's milk or infant formula. The vouchers are processed by swiping a barcode that subtracts the voucher value (£3.10) from the total. They are not electronically matched to specific food items. There is a reminder printed on each voucher about which foods may be purchased, along with a warning about prosecution, but the evidence suggests that some customers and retailers appear to disregard this information.

For peer review only

Retailers who are registered to accept Healthy Start vouchers have some discretion over how vigilantly they check what vouchers are spent on [context].[6, 15] Women may put pressure on retailers to 'bend the rules' or make exceptions [mechanism].[6, 15-17, 32] Some retailers may decide to 'turn a blind eye' because they feel duty bound to help families in whatever ways they can[mechanism][6] or because they prefer to avoid conflict [mechanisms].[16] This enables women to exchange the vouchers for alternative food or non-food items [outcomes].[6, 16]

> "But you have to realise that I get people coming in here, they are buying £1 pound of electricity every day. £1. That must run out after an hour. How do they live? And in the winter, it really does get very cold and they come in and ask me if they can use the voucher for electricity. What can I do? I can't see them living in the flat with young children, with no heating, it's so cold. So I do let them do that. They come in and show me their empty wallet and I have to believe them and I do sell gas and electricity for the voucher. You can report that back. I don't care, what can I do?" (Retailer, UK; page 69)[6]

3. Disempowerment

Pregnant women (and later their young children) are the intended beneficiaries of Healthy Start, but some women may not be empowered to make decisions about how to use the vouchers themselves. The vouchers are posted to women at their home address, but there is no name printed on the actual vouchers and no identification is required at the checkout, so there is nothing to stop other people from using them. Regardless of what is bought with the vouchers, and who benefits, this would surely be considered an unintended outcome of the programme.

Women may not be empowered to make decisions about household resources or food shopping, such as pregnant teenagers who live with their parents [context],[6, 47] or women who live in large, multi-generational households [context].[43] Women who are disempowered are more likely to hand over their Healthy Start vouchers to other family members [mechanism][43] who then decide how they are used [outcome].[43]

For peer review only

> "She (mom) makes most of the decisions. We get the same thing every time we go shopping." (African-American mother living in multi-generational household, US; page 5)[43]

DISCUSSION

Statement of principal findings

This realist review identified two main outcome strands for low-income pregnant women using food vouchers from the Healthy Start programme: dietary improvements (intended) and financial assistance (unintended). Three evidence-informed programme theories were developed and refined to explain how aspects of context (and mechanisms) may generate these outcomes: the 'relative value' of healthy eating (prioritisation of resources); retailer discretion (pressure to 'bend the rules'); the influence of other family members (disempowerment).

Strengths of this review

This was the first study to use a realist, theory-driven approach to investigate how low-income pregnant women use Healthy Start vouchers, in what circumstances, and why. The inclusion of relevant studies from a similar programme in the US (WIC) provided insights and explanations beyond what was available from the Healthy Start literature. This was the first time that researchers have attempted to articulate, develop and test programme theories about how Healthy Start works. Our findings suggest that Healthy Start vouchers are not always used to achieve dietary improvements. Some low-income pregnant women may need to receive more support to increase the perceived value of healthy eating. Modifications to the voucher verification system would help to prevent the vouchers being used by other people and exchanged for alternative items. We hope this review will stimulate discussions about future evaluation needs and programme development.

Limitations of this review

We were aware from the outset that some of the evidence from WIC studies would not be transferable to Healthy Start due to population and programme differences. Further evidence from the UK is needed to develop some of the other candidate theories we identified. This review focused on women's outcomes from the programme, and the aspects of context explored in our theories were individual (women's values and perceptions) and interpersonal

For peer review only

(interactions with retailers and other family members).[8] We did not find sufficient evidence to link our CMO configurations with sociodemographic and cultural characteristics, such as which groups of women are more/less likely to value healthy eating. Future reviews in this area could include wider evidence on voucher programmes to provide insights about how the mechanisms we have identified might operate in different contexts and different programmes. This was beyond the scope of this review due to time and resource limitations. Finally, we did not explore theories relating to Healthy Start vitamins, women's decisions about infant feeding or children's outcomes. These would all be worthy areas for realist investigations.

Comparisons with existing literature and theories

This study builds on two previous evaluations of Healthy Start, which highlighted different ways of using the food vouchers: some women used the vouchers to access healthy foods that they otherwise could not afford, whereas other women used them to save money on foods they had already planned to buy and reallocated the money for other things.[6, 7] Our realist review has shown how 'substitution effects' (i.e. using the vouchers as an alternative method of payment) may reduce the potential impact on women's nutritional outcomes and some women may experience no dietary improvements at all. In addition, we have identified aspects of context and causal mechanisms that are likely to be important in determining outcome patterns for low-income pregnant women.

Our first programme theory relates to the economics of decision making. If women value healthy eating and want to do everything they can to give their baby the best possible start in life, these beliefs and motivations will influence the decision making process when it comes to using the vouchers. However, other factors will also influence the decision making process and women must consider whether additional fruits and vegetables (or cow's milk) are the most important things they need. Frick considered the everyday economic analyses that take place at family level in relation to infant and young child feeding, whereby mothers and other family members must decide how to allocate financial resources, weighing up food choices and nutrition against a range of other considerations.[51] He described how societal and individual values influence these trade-offs between nutrition and other priorities. Decisions about how to use Healthy Start vouchers are subject to similar trade-offs through the mechanism of prioritisation. Attree found that low-income women 'strategically adjust' to poverty by prioritising or 'juggling' what they spend money on.[52] Food may be ranked below other basic needs such as rent and household bills, with more flexibility to cut back on

For peer review only

healthy items like fruits and vegetables. Therefore, Healthy Start may be seen as a way to manage financially by reducing food expenditure. The programme provides additional resources to (ideally) enable low-income pregnant women to improve their diets, but only women who value healthy eating (and the associated health benefits for mother and child) are likely to use the vouchers in this intended way. A recent taxonomy of behavior change techniques defined 'incentives' as rewarding and contingent on behavior change.[53] Healthy Start does not fit this definition and should not be assumed to encourage healthy choices for all beneficiaries.

Our second programme theory relates to retailers who misuse the Healthy Start programme by allowing women to exchange vouchers for alternative items. It is presented under the context of retailer discretion, which highlights weaknesses in the system, but this theory also relates to the context of women who value other things above healthy eating. The evidence suggests that some retailers may bend the rules because they feel they are acting in the best interests of the customer. This is similar to 'responsible subversion' identified among health professionals who admitted to bending or breaking the rules for what they perceived to be patient benefits, despite contravening evidence-based practice guidance.[54] However, there may be other (unscrupulous) reasons why retailers bend the rules and further evidence is needed to develop and explore this programme theory more fully.

Finally, our third programme theory relates to women who may not be empowered to decide how to use their Healthy Start vouchers. Their choices may be heavily influenced (or constrained) by significant others, for example a partner, mother or mother-in-law, who may take charge of food shopping and allocation of household resources. Similar issues have been identified in relation to decisions about infant feeding: women are surrounded by networks of people who participate in decision making, so they may be unable to exercise their 'right to choose' despite knowing what the options are and possessing their own opinions.[55] This may be particularly the case in communities where there are high levels of interdependence within the extended family network.

Healthy Start is dependent on individual agency to achieve dietary improvements, in contrast with other types of nutrition interventions that can be said to be less dependent on individual agency (such as food fortification). Our evidence-informed programme theories illustrate how aspects of context may enable or constrain women's agency. A recent paper by the

For peer review only

Centre for Diet and Activity Research (CEDAR) considered the role of individual agency in public health interventions, concluding that 'low agency' interventions are more likely to be effective and equitable by reducing the need for individual decisions.[56] Food vouchers for free fruits and vegetables were positioned in the middle of a continuum of the degree of agency required to benefit from the intervention. This review highlights some ways in which the level of agency required could be reduced in the Healthy Start programme, such as by 'tightening up' the system for verifying who uses the vouchers and what they are exchanged for. However, agency cannot be eliminated from food voucher programmes and therefore this review contributes to ongoing debates about how public health interventions should be designed to maximize outcome effectiveness.

Agency is synonymous with realist mechanisms (the reasoning and reactions of individuals in response to the resources offered by the programme) and this review illustrates the contribution of realist methodology to understanding the differential impacts of public health interventions or programmes.

CONCLUSION

This realist review suggests that some low-income pregnant women use Healthy Start vouchers to increase their consumption of fruits and vegetables and plain cow's milk (intended outcome: dietary improvements) whereas other women use them to reduce food expenditure and save money for other things (unintended outcome: financial assistance). We have identified some aspects of context (the 'relative value' of healthy eating, retailer discretion and the influence of other family members) and mechanisms (prioritisation of resources, pressure to 'bend the rules' and disempowerment) that are likely to be important in determining these outcomes. Further evidence is needed to understand how low-income pregnant women could be better supported to prioritise healthy eating and use Healthy Start vouchers to improve their diets during pregnancy – in particular to buy more fruits and vegetables. This may include ways of 'tightening up' the programme to reduce the amount of agency required but also considering ways in which women may be supported to become more empowered to choose.

For peer review only

STATEMENTS

Acknowledgements: The authors would like to thank the stakeholder group for their contributions to theory development, and Eleanor Kotas (Information Specialist) for her assistance with the electronic database searches.

Contributors: HO was the lead reviewer, while other authors completed the second reviewer tasks (as indicated in the Methods). All authors contributed to the development of programme theories through regular discussions. HO drafted the initial manuscript and all authors contributed to the final manuscript.

Funding: This work was supported by a PhD Studentship (2014-2017) from the Collaboration for Leadership in Applied Health Research and Care North West Coast (CLAHRC NWC).

Declaration: The views expressed are those of the authors and not necessarily those of the DWP, NHS, NIHR or the Department of Health.

Competing interests: None declared.

Data sharing statement: No additional data are available.

For peer review only

REFERENCES

1 Acheson D. Independent Inquiry into Inequalities in Health Report. London: The Stationery Office 1998.

2 Committee on Medical Aspects of Food and Nutrition Policy. Scientific review of the Welfare Food Scheme. London, 2002.

3 McFadden A, Green JM, McLeish J, et al. Healthy Start vitamins – a missed opportunity: findings of a multimethod study, *BMJ Open* 2015;5.

4 Department of Health. Healthy Start: Equality impact assessment. London, 2010.

5 Dyson L, Renfrew MJ, Jenkins R, et al. Approaches to evaluating Healthy Start – a scoping review. Public Health Research Consortium, 2007.

6 Lucas PJ, Jessiman T, Cameron A, et al. Healthy Start Vouchers Study: The Views and Experiences of Parents, Professionals and Small Retailers in England. Bristol: University of Bristol, 2013.

7 McFadden A, Fox-Rushby J, Green JM, et al. Healthy Start: Understanding the use of vouchers and vitamins. Dundee: University of Dundee, 2013.

8 Pawson R. Evidence-based policy: a realist perspective. London: Sage 2006:196.

9 Institute of Medicine of the National Academies. WIC Food Packages: Time for a Change. Institute of Medicine, 2005.

10 United States Department of Agriculture: Food and Nutrition Service. WIC Studies: http://www.fns.usda.gov/ops/wic-studies. Date accessed: February 2015.

11 The RAMESES Project. Quality Standards for Realist Synthesis (for researchers and peer-reviewers): http://www.ramesesproject.org/media/RS_qual_standards_researchers.pdf. Date accessed: November 2014.

12 Wong G. Special Invited Editorial: Getting Started With Realist Research, *International Journal of Qualitative Methods* 2015;14.

13 Thomas J, Harden A. Methods for the thematic synthesis of qualitative research in systematic reviews, *BMC Medical Research Methodology* 2008;8:45.

14 Ford JA, Wong G, Jones AP, et al. Access to primary care for socioeconomically disadvantaged older people in rural areas: a realist review, *BMJ Open* 2016;6.

15 Department of Health. Healthy Start: Retailer research summary. London, 2012.

16 Hills D, Child C, Junge K, Wilkinson E, Sullivan F. Healthy Start: Rapid evaluation of early impact on beneficiaries, health professionals, retailers and contractors. London: Symbia/Tavistock Institute, 2006.

For peer review only

17 Andreyeva T, Middleton AE, Long MW, et al. Food retailer practices, attitudes and beliefs about the supply of healthy foods, *Public Health Nutr* 2011;14:1024-31.

18 Andreyeva T, Luedicke J, Henderson KE, et al. Grocery store beverage choices by participants in federal food assistance and nutrition programs, *Am J Prev Med* 2012;43:411-8.

19 Andreyeva T, Luedicke J, Tripp AS, et al. Effects of reduced juice allowances in food packages for the women, infants, and children program, *Pediatrics* 2013;131:919-27.

20 Andreyeva T, Luedicke J. Federal food package revisions: effects on purchases of whole-grain products, *Am J Prev Med* 2013;45:422-9.

21 Andreyeva T, Luedicke J, Henderson KE, et al. The positive effects of the revised milk and cheese allowances in the special supplemental nutrition program for women, infants, and children, *Journal of the Academy of Nutrition & Dietetics* 2014;114:622-30.

22 Andreyeva T, Luedicke J. Incentivizing fruit and vegetable purchases among participants in the Special Supplemental Nutrition Program for Women, Infants, and Children, *Public Health Nutr* 2015;18:33-41.

23 Ayala GX, Laska MN, Zenk SN, et al. Stocking characteristics and perceived increases in sales among small food store managers/owners associated with the introduction of new food products approved by the Special Supplemental Nutrition Program for Women, Infants, and Children, *Public Health Nutr* 2012;15:1771-9.

24 Bailey LB, O'Farrell-Ray B, Mahan CS, et al. Vitamin B6, iron and folacin status of pregnant women, *Nutr Res* 1983;3:783-93.

25 Bertmann FM, Barroso C, Ohri-Vachaspati P, et al. Women, infants, and children cash value voucher (CVV) use in Arizona: a qualitative exploration of barriers and strategies related to fruit and vegetable purchases, *Journal of Nutrition Education & Behavior* 2014;46:S53-8.

26 Black MM, Hurley KM, Oberlander SE, et al. Participants' comments on changes in the revised special supplemental nutrition program for women, infants, and children food packages: the Maryland food preference study, *J Am Diet Assoc* 2009;109:116-23.

27 Chen DY, Gazmararian JA. Impact of personal preference and motivation on fruit and vegetable consumption of WIC-participating mothers and children in Atlanta, GA, *Journal of Nutrition Education & Behavior* 2014;46:62-7.

28 Endres J, Dunning S, Poon SW, et al. Older pregnant women and adolescents: nutrition data after enrollment in WIC, *J Am Diet Assoc* 1987;87:1011.

29 Endres JM, Sawicki M, Casper JA. Dietary assessment of pregnant women in a supplemental food program. *J Am Diet Assoc* 1981;79:121-6.

30 Ettienne-Gittens R, McKyer ELJ, Odum M, et al. Rural versus Urban Texas WIC participants' fruit and vegetable consumption, *Am J Health Behav* 2013;37:130-40.

For peer review only

31 Farrior ES, Ruwe CH. Women, infants and children program. Prenatal participation and dietary intakes, *Nutr Res* 1987;7:451-549.

32 Gittelsohn J, Laska MN, Andreyeva T, et al. Small retailer perspectives of the 2009 women, infants and children program food package changes, *Am J Health Behav* 2012;36:655-65.

33 Herman DR, Harrison GG, Afifi AA, et al. The Effect of the WIC Program on Food Security Status of Pregnant, First-Time Participants, *Family Economics & Nutrition Review* 2004;16:21.

34 Herman DR, Harrison GG, Jenks E. Choices made by low-income women provided with an economic supplement for fresh fruit and vegetable purchase, *J Am Diet Assoc* 2006;106:740-4.

35 Herman DR, Harrison GG, Afifi AA, et al. Effect of a targeted subsidy on intake of fruits and vegetables among low-income women in the Special Supplemental Nutrition Program for Women, Infants, and Children, *Am J Public Health* 2008;98:98-105.

36 Horswill LJ, Yap C. Consumption of foods from the WIC food packages of Chinese prenatal patients on the US west coast, *J Am Diet Assoc* 1999;99:1549-53.

37 Kong A, Odoms-Young A, Schiffer LA, et al. Racial/ethnic differences in dietary intake among WIC families prior to food package revisions, *Journal of Nutrition Education & Behavior* 2013;45:39-46.

38 Kong A, Odoms-Young A, Schiffer LA, et al. The 18-month impact of special supplemental nutrition program for women, infants, and children food package revisions on diets of recipient families, *Am J Prev Med* 2014;46:543-51.

39 Meiqari L, Torre L, Gazmararian JA. Exploring the Impact of the New WIC Food Package on Low-Fat Milk Consumption Among WIC Recipients: A Pilot Study, *Journal of Health Care for the Poor & Underserved* 2015;26:712-25.

40 Nestor B, McKenzie J, Hasan N, et al. Client satisfaction with the nutrition education component of the California WIC program, *J Nutr Educ* 2001;33:83-94.

41 Odoms-Young A, Kong A, Schiffer LA, et al. Evaluating the initial impact of the revised Special Supplemental Nutrition Program for Women, Infants, and Children (WIC) food packages on dietary intake and home food availability in African-American and Hispanic families, *Public Health Nutr* 2014;17:83-93.

42 Pehrsson PR, Moser-Veillon PB, Sims LS, et al. Postpartum iron status in nonlactating participants and nonparticipants in the Special Supplemental Nutrition Program for Women, Infants, and Children, *Am J Clin Nutr* 2001;73:86-92.

43 Reyes NR, Klotz AA, Herring SJ. A qualitative study of motivators and barriers to healthy eating in pregnancy for low-income, overweight, African-American mothers, *Journal of the Academy of Nutrition & Dietetics* 2013;113:1175-81.

For peer review only

44 Rush D, Sloan NL, Leighton J, et al. The National WIC Evaluation: evaluation of the Special Supplemental Food Program for Women, Infants, and Children. V. Longitudinal study of pregnant women, *Am J Clin Nutr* 1988;48:439-83.

45 Rush D, Kurzon MR, Seaver WB, et al. The National WIC Evaluation: evaluation of the Special Supplemental Food Program for Women, Infants, and Children. VII. Study of food expenditures, *Am J Clin Nutr* 1988;48:512-9.

46 Swensen AR, Harnack LJ, Ross JA. Nutritional assessment of pregnant women enrolled in the Special Supplemental Program for Women, Infants, and Children (WIC), *J Am Diet Assoc* 2001;101:903-8.

47 Treiman K, Freimuth V, Damron D, et al. Attitudes and behaviors related to fruits and vegetables among low-income women in the WIC program, *J Nutr Educ Behav* 1996;28:149-56.

48 Watts V, Rockett H, Baer H, et al. Assessing diet quality in a population of low-income pregnant women: a comparison between Native Americans and whites, *Maternal & Child Health Journal* 2007;11:127-36.

49 Whaley SE, Ritchie LD, Spector P, et al. Revised WIC food package improves diets of WIC families, *Journal of Nutrition Education & Behavior* 2012;44:204-9.

50 Wunderlich SM, Hongu NK, Courter A, et al. Nutrient intake and nutritional status of low-income pregnant women, *Topics in Clinical Nutrition* 1996;12:66-74.

51 Frick KD. Use of Economics to Analyse Policies to Promote Breastfeeding. In: Dykes F, Hall Moran V, eds. Infant and Young Child Feeding. Chichester: Wiley-Blackwell 2009:181-196.

52 Attree P. Low-income mothers, nutrition and health: A systematic review of qualitative evidence, *Matern Child Nutr* 2005;1:227-40.

53 Abraham C, Michie S. A Taxonomy of Behavior Change Techniques Used in Interventions, *Health Psychol* 2008;27:379-87.

54 Furber CM, Thomson AM. ‘Breaking the rules’ in baby-feeding practice in the UK: deviance and good practice? *Midwifery* 2006;22:365-76.

55 Blystad A, van Esterik P, de Paoli MM, et al. Reflections on global policy documents and the WHO's infant feeding guidelines: Lessons learnt, *Int Breastfeeding J* 2010;5.

56 Adams J, Mytton O, White M, et al. Why Are Some Population Interventions for Diet and Obesity More Equitable and Effective Than Others? The Role of Individual Agency, *PLoS Med* 2016;13:e1001990.

For peer review only

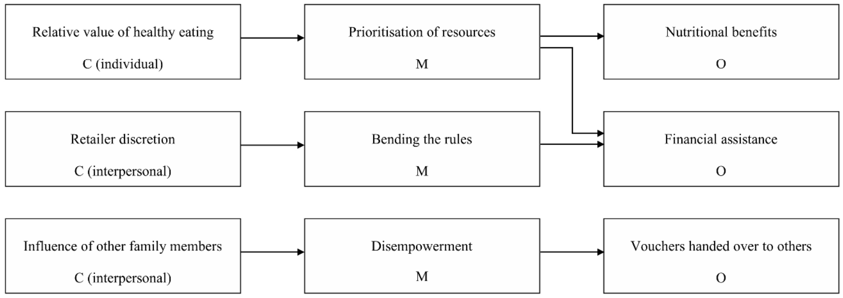

Summary of programme theories about how low-income pregnant women use Healthy Start vouchers

296x209mm (300 x 300 DPI)

review only

Supplementary file 1. Characteristics of studies included in review (n=38)

**Full citation**	**Intervention (country)**	**Study design and data collection methods**	**Contribution to data extraction (C/M/O) and synthesis (programme theories 1-3)**					
			**C**	**M**	**O**	**1**	**2**	**3**
Department of Health. Healthy Start: Retailer research summary. London, 2012.	Healthy Start (UK)	Methods unclear	✓	✓			✓	
Hills D, Child C, Junge K, Wilkinson E, Sullivan F. Healthy Start: Rapid evaluation of early impact on beneficiaries, health professionals, retailers and contractors. London: Symbia/Tavistock Institute, 2006.	Healthy Start (UK)	Rapid evaluation; quantitative surveys and qualitative feedback		✓	✓		✓	
Lucas PJ, Jessiman T, Cameron A, et al. Healthy Start Vouchers Study: The Views and Experiences of Parents, Professionals and Small Retailers in England. Bristol: University of Bristol, 2013.	Healthy Start (UK)	Qualitative study; in-depth interviews	✓	✓	✓	✓	✓	✓
McFadden A, Fox-Rushby J, Green JM, et al. Healthy Start: Understanding the use of vouchers and vitamins. Dundee: University of Dundee, 2013.	Healthy Start (UK)	Focus groups; online consultations; workshops and telephone interviews	✓	✓	✓	✓		
Andreyeva T, Middleton AE, Long MW, et al. Food retailer practices, attitudes and beliefs about the supply of healthy foods, *Public Health Nutr* 2011;14:1024-31.	WIC (US)	Before/after study; structured interviews to assess perceived customer demand for healthy foods (mostly quantitative)		✓	✓		✓	
Andreyeva T, Luedicke J, Henderson KE, et al. Grocery store beverage choices by participants in federal food assistance and nutrition programs, *Am J Prev Med* 2012;43:411-8.	WIC (US)	Cross-sectional study; electronic sales data from WIC retailers			✓			
Andreyeva T, Luedicke J, Tripp AS, et al. Effects of reduced juice allowances in food packages for the women, infants, and children program, *Pediatrics* 2013;131:919-27.	WIC (US)	Before/after study; electronic sales data from WIC retailers			✓			

For peer review only

Full citation	Intervention (country)	Study design and data collection methods	Contribution to data extraction (C/M/O) and synthesis (programme theories 1-3)					
			C	M	O	1	2	3
Andreyeva T, Luedicke J. Federal food package revisions: effects on purchases of whole-grain products, *Am J Prev Med* 2013;45:422-9.	WIC (US)	Before/after study; electronic sales data from WIC retailers			✓			
Andreyeva T, Luedicke J, Henderson KE, et al. The positive effects of the revised milk and cheese allowances in the special supplemental nutrition program for women, infants, and children, *Journal of the Academy of Nutrition & Dietetics* 2014;114:622-30.	WIC (US)	Before/after study; electronic sales data from WIC retailers			✓			
Andreyeva T, Luedicke J. Incentivizing fruit and vegetable purchases among participants in the Special Supplemental Nutrition Program for Women, Infants, and Children, *Public Health Nutr* 2015;18:33-41.	WIC (US)	Before/after study; electronic sales data from WIC retailers			✓			
Ayala GX, Laska MN, Zenk SN, et al. Stocking characteristics and perceived increases in sales among small food store managers/owners associated with the introduction of new food products approved by the Special Supplemental Nutrition Program for Women, Infants, and Children, *Public Health Nutr* 2012;15:1771-9.	WIC (US)	Cross-sectional study; in-depth interviews to assess perceived changes in sales of WIC foods (quantitative data from closed questions)			✓			
Bailey LB, O'Farrell-Ray B, Mahan CS, et al. Vitamin B6, iron and folacin status of pregnant women, *Nutr Res* 1983;3:783-93.	WIC (US)	Cross-sectional study; dietary assessment using 24 hour recalls; blood tests for nutrient status			✓			
Bertmann FM, Barroso C, Ohri-Vachaspati P, et al. Women, infants, and children cash value voucher (CVV) use in Arizona: a qualitative exploration of barriers and strategies related to fruit and vegetable purchases, *Journal of Nutrition Education & Behavior* 2014;46:S53-8.	WIC (US)	Qualitative study; focus groups to explore attitudes and behaviours related to FV voucher use	✓		✓			

For peer review only

Full citation	Intervention (country)	Study design and data collection methods	Contribution to data extraction (C/M/O) and synthesis (programme theories 1-3)					
			C	M	O	1	2	3
Black MM, Hurley KM, Oberlander SE, et al. Participants' comments on changes in the revised special supplemental nutrition program for women, infants, and children food packages: the Maryland food preference study, *J Am Diet Assoc* 2009;109:116-23.	WIC (US)	Cross-sectional study; questionnaire to assess current and anticipated consumption; focus groups to explore reactions to proposed changes to WIC package			✓			
Chen DY, Gazmararian JA. Impact of personal preference and motivation on fruit and vegetable consumption of WIC-participating mothers and children in Atlanta, GA, *Journal of Nutrition Education & Behavior* 2014;46:62-7.	WIC (US)	Cross-sectional study; questionnaire to assess FV consumption			✓			
Endres J, Dunning S, Poon SW, et al. Older pregnant women and adolescents: nutrition data after enrollment in WIC, *J Am Diet Assoc* 1987;87:1011.	WIC (US)	Cross-sectional study; dietary assessment using 24 hour recalls			✓			
Endres JM, Sawicki M, Casper JA. Dietary assessment of pregnant women in a supplemental food program. *J Am Diet Assoc* 1981;79:121-6.	WIC (US)	Cross-sectional study; dietary assessment using 24 hour recalls			✓			
Ettienne-Gittens R, McKyer ELJ, Odum M, et al. Rural versus Urban Texas WIC participants' fruit and vegetable consumption, *Am J Health Behav* 2013;37:130-40.	WIC (US)	Cross-sectional study; questionnaire to assess FV consumption and variety			✓			
Farrior ES, Ruwe CH. Women, infants and children program. Prenatal participation and dietary intakes, *Nutr Res* 1987;7:451-549.	WIC (US)	Before/after study; dietary assessment using 24 hour recalls			✓			
Gittelsohn J, Laska MN, Andreyeva T, et al. Small retailer perspectives of the 2009 women, infants and children program food package changes, *Am J Health Behav* 2012;36:655-65.	WIC (US)	Qualitative study; in-depth interviews with small store owners and managers	✓	✓	✓		✓	

For peer review only

Full citation	Intervention (country)	Study design and data collection methods	Contribution to data extraction (C/M/O) and synthesis (programme theories 1-3)					
			C	M	O	1	2	3
Herman DR, Harrison GG, Afifi AA, et al. The Effect of the WIC Program on Food Security Status of Pregnant, First-Time Participants, *Family Economics & Nutrition Review* 2004;16:21.	WIC (US)	Longitudinal study; US Food Security Survey Module; qualitative interviews on use of WIC food package as income transfer (presented as quantitative data)			✓	✓		
Herman DR, Harrison GG, Jenks E. Choices made by low-income women provided with an economic supplement for fresh fruit and vegetable purchase, *J Am Diet Assoc* 2006;106:740-4.	WIC (US)	Cross-sectional study; data from retailers on FV purchases			✓			
Herman DR, Harrison GG, Afifi AA, et al. Effect of a targeted subsidy on intake of fruits and vegetables among low-income women in the Special Supplemental Nutrition Program for Women, Infants, and Children, *Am J Public Health* 2008;98:98-105.	WIC (US)	Longitudinal study; dietary assessment using 24 hour recalls			✓			
Horswill LJ, Yap C. Consumption of foods from the WIC food packages of Chinese prenatal patients on the US west coast, *J Am Diet Assoc* 1999;99:1549-53.	WIC (US)	Cross-sectional study; dietary assessment using FFQ	✓		✓			
Kong A, Odoms-Young A, Schiffer LA, et al. Racial/ethnic differences in dietary intake among WIC families prior to food package revisions, *Journal of Nutrition Education & Behavior* 2013;45:39-46.	WIC (US)	Cross-sectional study; dietary assessment using 24 hour recalls	✓		✓			
Kong A, Odoms-Young A, Schiffer LA, et al. The 18-month impact of special supplemental nutrition program for women, infants, and children food package revisions on diets of recipient families, *Am J Prev Med* 2014;46:543-51.	WIC (US)	Longitudinal study; dietary assessment using 24 hour recalls (baseline data from Kong et al. 2013)	✓		✓			

For peer review only

Full citation	Intervention (country)	Study design and data collection methods	Contribution to data extraction (C/M/O) and synthesis (programme theories 1-3)					
			C	M	O	1	2	3
Meiqari L, Torre L, Gazmararian JA. Exploring the Impact of the New WIC Food Package on Low-Fat Milk Consumption Among WIC Recipients: A Pilot Study, *Journal of Health Care for the Poor & Underserved* 2015;26:712-25.	WIC (US)	Before/after study; questionnaire to assess primary consumption of low-fat milk (yes/no)			✓			
Nestor B, McKenzie J, Hasan N, et al. Client satisfaction with the nutrition education component of the California WIC program, *J Nutr Educ* 2001;33:83-94.	WIC (US)	Cross-sectional study; client satisfaction survey; focus groups to assess client experiences and satisfaction	✓	✓	✓			
Odoms-Young A, Kong A, Schiffer LA, et al. Evaluating the initial impact of the revised Special Supplemental Nutrition Program for Women, Infants, and Children (WIC) food packages on dietary intake and home food availability in African-American and Hispanic families, *Public Health Nutr* 2014;17:83-93.	WIC (US)	Longitudinal study; dietary assessment using 24 hour recalls (baseline data from Kong et al. 2013) and questionnaire to assess home food availability			✓			
Pehrsson PR, Moser-Veillon PB, Sims LS, et al. Postpartum iron status in nonlactating participants and nonparticipants in the Special Supplemental Nutrition Program for Women, Infants, and Children, *Am J Clin Nutr* 2001;73:86-92.	WIC (US)	Longitudinal study; iron status using FFQ and blood tests			✓			
Reyes NR, Klotz AA, Herring SJ. A qualitative study of motivators and barriers to healthy eating in pregnancy for low-income, overweight, African-American mothers, *Journal of the Academy of Nutrition & Dietetics* 2013;113:1175-81.	WIC (US)	Qualitative study; semi-structured interviews to explore motivators and barriers to healthy eating	✓	✓	✓			✓

For peer review only

Full citation	Intervention (country)	Study design and data collection methods	Contribution to data extraction (C/M/O) and synthesis (programme theories 1-3)					
			C	M	O	1	2	3
Rush D, Sloan NL, Leighton J, et al. The National WIC Evaluation: evaluation of the Special Supplemental Food Program for Women, Infants, and Children. V. Longitudinal study of pregnant women, *Am J Clin Nutr* 1988;48:439-83.	WIC (US)	Before/after study; food expenditure assessed by recall (and diary)			✓			
Rush D, Kurzon MR, Seaver WB, et al. The National WIC Evaluation: evaluation of the Special Supplemental Food Program for Women, Infants, and Children. VII. Study of food expenditures, *Am J Clin Nutr* 1988;48:512-9.	WIC (US)	Longitudinal study; dietary assessment using 24 hour recalls			✓			
Swensen AR, Harnack LJ, Ross JA. Nutritional assessment of pregnant women enrolled in the Special Supplemental Program for Women, Infants, and Children (WIC), *J Am Diet Assoc* 2001;101:903-8.	WIC (US)	Cross-sectional study; dietary assessment using FFQ; blood tests for iron status			✓			
Treiman K, Freimuth V, Damron D, et al. Attitudes and behaviors related to fruits and vegetables among low-income women in the WIC program, *J Nutr Educ Behav* 1996;28:149-56.	WIC (US)	Qualitative study; focus groups to explore attitudes and behaviours related to FV; central location intercept interviews (questionnaires)	✓	✓	✓	✓		✓
Watts V, Rockett H, Baer H, et al. Assessing diet quality in a population of low-income pregnant women: a comparison between Native Americans and whites, *Maternal & Child Health Journal* 2007;11:127-36.	WIC (US)	Cross-sectional study; FFQ to assess diet quality			✓			
Whaley SE, Ritchie LD, Spector P, et al. Revised WIC food package improves diets of WIC families, *Journal of Nutrition Education & Behavior* 2012;44:204-9.	WIC (US)	Cross-sectional study; questionnaire to assess usual dietary habits and changes compared to 6 months earlier			✓			

For peer review only

Full citation	Intervention (country)	Study design and data collection methods	Contribution to data extraction (C/M/O) and synthesis (programme theories 1-3)					
			C	M	O	1	2	3
Wunderlich SM, Hongu NK, Courter A, et al. Nutrient intake and nutritional status of low-income pregnant women, *Topics in Clinical Nutrition* 1996;12:66-74.	WIC (US)	Cross-sectional study; dietary assessment using 24 hour recalls			✓			

WIC = Special Supplemental Program for Women, Infants, and Children; FV = fruits and vegetables; Programme theories: 1. Prioritisation of resources; 2. Bending the rules; 3. Disempowerment

For peer review only

List of items to be included when reporting a realist synthesis

**TITLE**			**Page**
1		In the title, identify the document as a realist synthesis or review	1
ABSTRACT			
2		While acknowledging publication requirements and house style, abstracts should ideally contain brief details of: the study's background, review question or objectives; search strategy; methods of selection, appraisal, analysis and synthesis of sources; main results; and implications for practice.	2
INTRODUCTION			
3	Rationale for review	Explain why the review is needed and what it is likely to contribute to existing understanding of the topic area.	4
4	Objectives and focus of review	State the objective(s) of the review and/or the review question(s). Define and provide a rationale for the focus of the review.	4-5
METHODS			
5	Changes in the review process	Any changes made to the review process that was initially planned should be briefly described and justified.	NA
6	Rationale for using realist synthesis	Explain why realist synthesis was considered the most appropriate method to use.	4
7	Scoping the literature	Describe and justify the initial process of exploratory scoping of the literature.	5-6
8	Searching processes	While considering specific requirements of the journal or other publication outlet, state and provide a rationale for how the iterative searching was done. Provide details on all the sources accessed for information in the review. Where searching in electronic databases has taken place, the details should include, for example, name of database, search terms, dates of coverage and date last searched. If individuals familiar with the relevant literature and/or topic area were contacted, indicate how they were identified and selected.	6
9	Selection and appraisal of documents	Explain how judgements were made about including and excluding data from documents, and justify these.	7-8
10	Data extraction	Describe and explain which data or information were extracted from the included documents and justify this selection.	8
11	Analysis and synthesis processes	Describe the analysis and synthesis processes in detail. This section should include information on the constructs analyzed and describe the analytic process.	9
RESULTS			
12	Document flow diagram	Provide details on the number of documents assessed for eligibility and included in the review with reasons for exclusion at each stage as well as an indication of their source of origin (for example, from searching databases, reference lists and so on).	9 and 21

For peer review only

TITLE			Page
		You may consider using the example templates (which are likely to need modification to suit the data) that are provided.	
13	Document characteristics	Provide information on the characteristics of the documents included in the review.	9-11
14	Main findings	Present the key findings with a specific focus on theory building and testing.	11-13
DISCUSSION			
15	Summary of findings	Summarize the main findings, taking into account the review's objective(s), research question(s), focus and intended audience(s).	13
16	Strengths, limitations and future research directions	Discuss both the strengths of the review and its limitations. These should include (but need not be restricted to) (a) consideration of all the steps in the review process and (b) comment on the overall strength of evidence supporting the explanatory insights which emerged.<br><br>The limitations identified may point to areas where further work is needed.	13-14
17	Comparison with existing literature	Where applicable, compare and contrast the review's findings with the existing literature (for example, other reviews) on the same topic.	14-15
18	Conclusion and recommendations	List the main implications of the findings and place these in the context of other relevant literature. If appropriate, offer recommendations for policy and practice.	15
19	Funding	Provide details of funding source (if any) for the review, the role played by the funder (if any) and any conflicts of interests of the reviewers.	16

Source of this checklist:

http://www.equator-network.org/reporting-guidelines/rameses-publication-standards-realist-syntheses/

http://www.ramesesproject.org/Standards_and_Training_materials.php

http://bmcmedicine.biomedcentral.com/articles/10.1186/1741-7015-11-21#Tab1

Page numbers refer to pdf document created during submission process.

For peer review only

PRISMA 2009 Flow Diagram

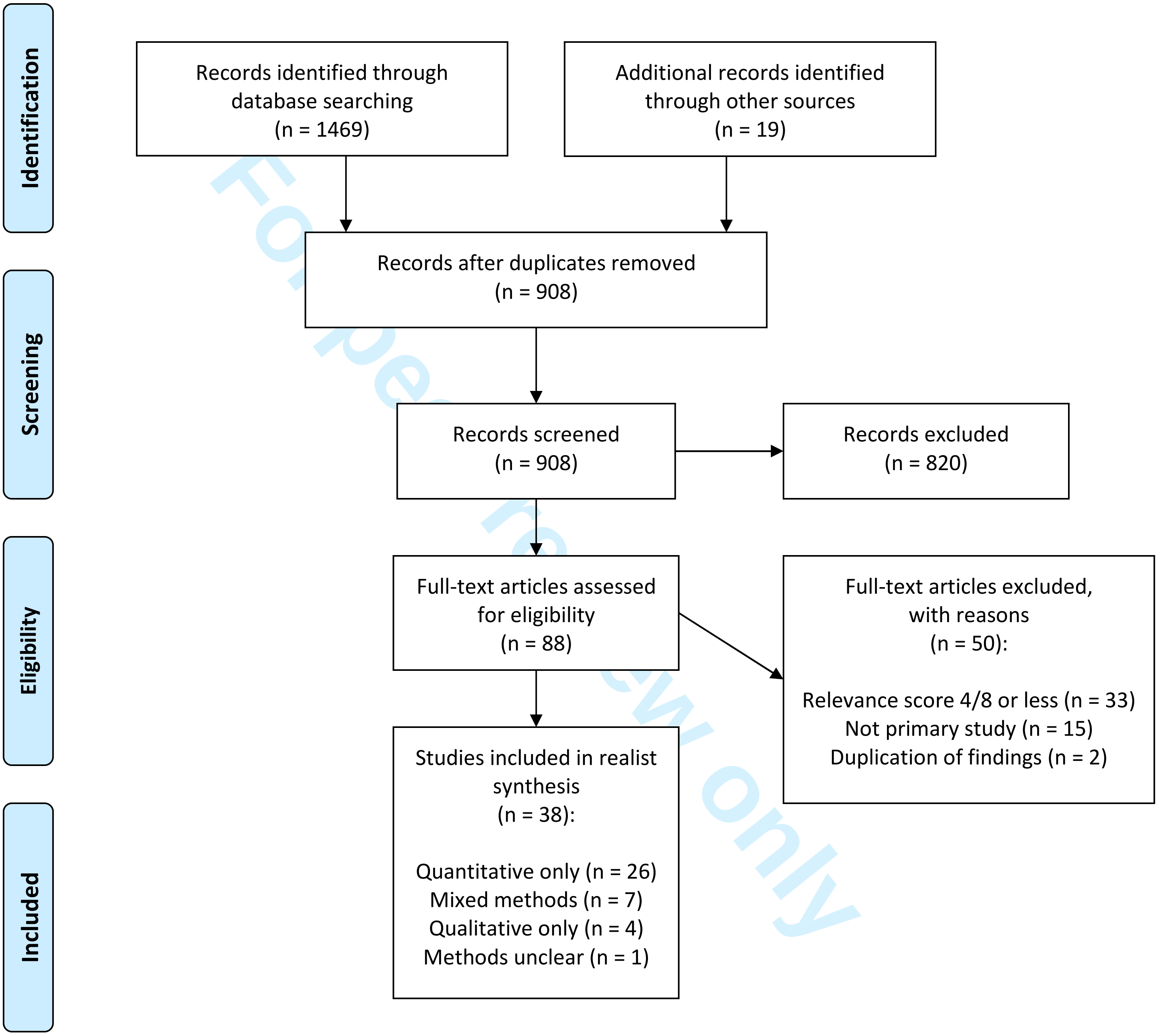

From: Moher D, Liberati A, Tetzlaff J, Altman DG, The PRISMA Group (2009). *P*referred *R*eporting *I*tems for *S*ystematic Reviews and *M*eta-*A*nalyses: The PRISMA Statement. PLoS Med 6(6): e1000097. doi:10.1371/journal.pmed1000097

For more information, visit www.prisma-statement.org.
